# Kinetic Model of Radical Ring-Opening Polymerization of Asymmetric Five-Membered Cyclic Ketene Acetals

**DOI:** 10.1021/acs.macromol.5c01438

**Published:** 2025-08-19

**Authors:** Shin-nosuke Nishimura, Marina Uryu, Tomoyuki Koga

**Affiliations:** Department of Molecular Chemistry and Biochemistry, Faculty of Science and Engineering, 12757 Doshisha University,1-3 Tatara Miyakodani, Kyotanabe, Kyoto 610-0321, Japan

## Abstract

Radical ring-opening polymerization RROP of cyclic ketene acetals (CKAs) provides a promising route to biodegradable polyesters. However, the mechanistic factors determining polymer structure are still not well understood, especially for CKAs with asymmetricly substituted rings. In this study, we investigate a series of five-membered CKAs bearing electron-donating alkoxymethyl groups at the 4-position, synthesized from bio-based precursors. Through detailed NMR analyses, DFT-calculated rate constants, and a comprehensive kinetic model, we clarify how 4-position substitution influences the balance between propagation, β-scission, and backbiting pathways. The model successfully reproduces the experimentally observed polymer structures across a wide range of temperatures and monomer concentrations, and its applicability extends to CKAs with varying alkoxy groups. The incorporation of ester linkages via ring-opening was confirmed by NMR and correlated with partial biodegradation in OECD 301F tests. These findings establish a predictive framework that links monomer structure for advancing the design of sustainable and biodegradable radical polymers.

## Graphical abstract

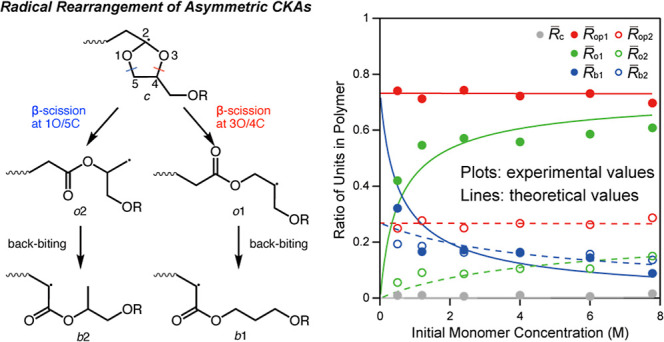

## Introduction

Biodegradable polymers have attracted significant attention in recent years due to their potential to address environmental concerns associated with persistent plastic waste.^1–3^ Among various classes of degradable polymers, those derived from cyclic ketene acetals (CKAs) have emerged as promising candidates for designing polyesters with tunable degradation rates and mechanical properties. CKAs undergo radical ring-opening polymerization (RROP), which introduces ester linkages into the polymer backbone while maintaining compatibility with conventional radical polymerization techniques.^4–9^ This unique combination of degradability and synthetic flexibility has led to growing interest in understanding and controlling the polymerization behavior of CKAs.^10–19^

However, the radical polymerization of CKAs involves complex reaction pathways that include reversible propagation, unimolecular β-scission, and intramolecular backbiting.^20–22^ These processes compete with each other during the polymerization, significantly affecting the microstructure and properties of the resulting polymers. Previous studies have shown that the size of the ring and the nature of substituents on the CKA monomer strongly influence the balance between these competing pathways.^8,9,23^ Notably, 5-membered CKAs such as 2-methylene-1,3-dioxolane (**C5**) exhibit particularly rich mechanistic behavior due to their multiple β-scission and rearrangement reactions.

To rationally design CKAs for specific material applications, a deeper understanding of how monomer structure affects polymerization kinetics and product distribution is essential.^16,24,25^ In this context, kinetic modeling supported by density functional theory (DFT) calculations has proven to be a powerful approach for elucidating the mechanistic details of RROP.^18,23,24,26–33^ Recent computational study has successfully estimated rate constants for key propagation and scission steps of CKAs, providing insights into the temperature and concentration dependences of polymer structure formation.^16^

In this study, we focused on a series of novel 5-membered CKAs bearing alkoxymethyl substituents at the 4-position (**C5-COR**, R = CH_3_ (**5a**), CH_2_CH_3_ (**5b**), CH_2_CH_2_OCH_3_ (**5c**), CH_2_Ph (**5d**)), synthesized from epichlorohydrin as a bioderived precursor.^34^ These monomers are therefore regarded as partially bioderived, and were subjected to polymerization (Scheme 1). We aim to investigate how these electron-donating substituents influence the radical polymerization behavior of CKAs by altering the reactivity of intermediate radicals. The polymerization of one representative monomer, **5a**, was studied in detail under various monomer concentrations and temperatures. Structural analyses of the resulting polymers were performed by NMR spectroscopy, and the experimental data were quantitatively compared with predictions from a kinetic model incorporating DFT-derived rate constants. Additionally, polymerization behavior of the parent unsubstituted monomer **C5** was examined to serve as a reference point. By integrating experimental and theoretical approaches, this work provides a comprehensive understanding of how 4-position substitution modulates the competition between propagation, β-scission, and backbiting pathways in RROP of 5-membered CKAs, and highlights the importance of substituent effects in the design of degradable radical polymers.

**Scheme 1:**
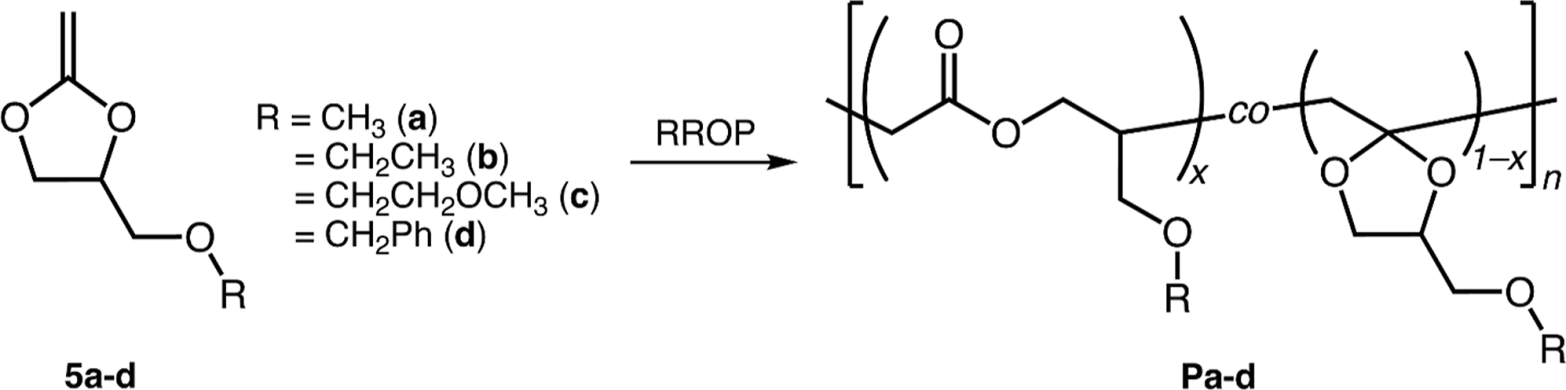
Radical Ring-Opening Polymerization (RROP) of 5-Membered Cyclic Ketene Acetal Bearing Alkoxymethyl Groups (**C5-COR**)

## Experimental Section

### Materials

Methanol, ethanol, hexane, diethyl ether, 2,2,2,-trifluoroacetic acid (TFA), sulfuric acid (H_2_SO_4_), ethyl acetate, magnesium sulfate anhydrous, sodium hydroxide (NaOH), and 2,2′-azobis­(isobutyronitrile) (AIBN) were purchased from Nacalai Tesque, Inc. (Japan). Boron trifluoride-ethyl ether complex (BF_3_–Et_2_O), epichlorohydrin, 2-methoxyethanol, benzyl alcohol, tetrabutylammonium bromide (TBAB), bromoacetaldehyde diethyl acetal, *o*-dichlorobenzen, potassium *tert*-butoxide, and di-*tert*-butyl peroxide (DTBP) were purchased from Tokyo Chemical Industry Co., Ltd. (Japan). Amberlyst 15­(H) was purchased from Sigma-Aldrich (USA). Potassium carbonate, tetrahydrofuran (THF, super dehydrated), and 2,2′-azobis­(*N*-butyl-2-methylpropionamide) (VAm-110) were purchased from FUJIFILM Wako Pure Chemical Co. (Japan).

### Measurements

The ^1^H and ^13^C NMR, DEPT90, DEPT135, ^1^H–^1^H COSY, ^1^H–^1^H TOCSY, ^1^H–^13^C HMQC, and ^1^H–^13^C HMBC analyses were conducted using a JNM-ECA500 (JEOL Resonance Co., Ltd., Japan) spectrometer (500 MHz). The high-resolution mass spectra (HRMS) were obtained using a direct analysis in real time (DART) ionization source (IonSense Inc., USA). Samples were analyzed in open air under ambient conditions using helium as the carrier gas, without the need for matrix or solvent. The instrument was calibrated with polyethylene glycol immediately before analysis to ensure high mass accuracy. The number-average molecular weight (*M*_n_) and the dispersity (*D̵* = *M*_w_/*M*_n_) of synthesized polymers were evaluated by SEC using a JASCO LC-net II/AD (JASCO Ltd., Japan) equipped with a refractive index (RI) detector. The measurements were conducted in THF at 40 °C (flow rate of 1.0 mL/min, column: KF-805L (Resonac Co., Japan)). Low-dispersity poly­(methyl methacrylate)­s (GL Sciences Inc., *M*_w_ = 2810, 5000, 10,290, 27,600, 60,150, 138,600, and 298,900) were used as calibration standards. The biodegradability of the polymer was evaluated according to the OECD 301F manometric respirometry method using an OxiTop respirometer system (WTW GmbH, Germany). A powdered polymer sample (10 mg) was dispersed in 100 mL of surface water collected from the Kizu-gawa River (Kyoto, Japan), which served as the microbial inoculum. The sealed test vessels were incubated at 22 °C under gentle stirring and in the dark to simulate natural environmental conditions. Carbon dioxide generated by microbial degradation was absorbed by NaOH placed in the headspace. The resulting pressure decrease, due to microbial oxygen consumption, was continuously recorded and used to calculate the biochemical oxygen demand (BOD). The theoretical oxygen demand (ThOD) was calculated from the known chemical structure of the polymer, and the extent of biodegradation was expressed as the percentage of BOD relative to ThOD. Each test was conducted in triplicate (*n* = 3), and the error bars reflect variability in microbial activity among replicates, likely due to differences in the microbial composition of the river water.

### Synthesis of 1-Chloro-3-methoxy-2-propanol (**1a**)

Methanol 10.48 g (327 mmol) and BF_3_–Et_2_O 3.87 g (27.3 mmol) were mixed and cooled in ice bath. Epichlorohydrin 20.11 g (217 mmol) was added dropwise to the mixture, and then the solution stirred for 2 h at ambient temperature. After reaction, water 20 mL was added to the reaction solution and extracted with dichloromethane 10 mL × 3. The organic layer was collected and dried over magnesium sulfate anhydrous. The solvent was removed in vacuo to give a crude. The objective molecule was collected by distillation (66–68 °C, 3 hPa) to obtain the pure 1-chloro-3-methoxy-2-propanol (**1a**) as a colorless liquid (12.96 g, 47.9%).

^1^H NMR (500 MHz, CDCl_3_, TMS) (Figure S1A) δ: 2.54 (s, 1H), 3.41 (s, 3H), 3.51 (d, 1H, ^3^ *J* = 5.2 Hz), 3.61 (m, 2H), 3.98 (m, 1H), 7.26 (residual internal CHCl_3_).

^13^C NMR (125 MHz, CDCl_3_, TMS) (Figure S1B) δ: 46.1, 59.4, 70.2, 73.2, 77.1 (CDCl_3_).

### Synthesis of 1-Chloro-3-ethoxy-2-propanol (**1b**)

This molecule was synthesized by the same method with **1a**. Ethanol 7.53 g (164 mmol) and BF_3_–Et_2_O 1.94 g (13.7 mmol) were mixed and cooled in ice bath. Epichlorohydrin 10.1 g (109 mmol) was added dropwise to the mixture, and then the solution stirred for 2 h at ambient temperature. After reaction, water 20 mL was added to the reaction solution and extracted with dichloromethane 10 mL × 3. The organic layer was collected and dried over magnesium sulfate anhydrous. The solvent was removed in vacuo to give a crude. The objective molecule was collected by distillation (64–66 °C, 2 hPa) to obtain the pure 1-chloro-3-ethoxy-2-propanol (**1b**) as a colorless liquid (8.28 g, 55.0%).

^1^H NMR (500 MHz, CDCl_3_, TMS) (Figure S2A) δ: 1.22 (t, 3H, 2.54, ^3^ *J* = 6.9 Hz), 2.54 (d, 1H, ^3^ *J* = 5.2 Hz), 3.55 (m, 4H), 3.62 (m, 2H), 3.98 (m, 1H), 7.26 (residual internal CHCl_3_).

^13^C NMR (125 MHz, CDCl_3_, TMS) (Figure S2B) δ: 15.1, 46.1, 67.0, 70.4, 71.1, 77.1 (CDCl_3_).

### Synthesis of 1-Chloro-3-(2-methoxyethoxy)-2-propanol (**1c**)

This molecule was synthesized by the same method with **1a**. 2-Methoxyethanol 12.45 g (164 mmol) and BF_3_–Et_2_O 1.94 g (13.7 mmol) were mixed and cooled in ice bath. Epichlorohydrin 10.1 g (109 mmol) was added dropwise to the mixture, and then the solution stirred for 2 h at ambient temperature. After reaction, water 20 mL was added to the reaction solution and extracted with dichloromethane 10 mL × 3. The organic layer was collected and dried over magnesium sulfate anhydrous. The solvent was removed in vacuo to give a crude. The objective molecule was collected by distillation (100–102 °C, 6 hPa) to obtain the pure 1-chloro-3-((methoxyethoxy)­methyl)-2-propanol (**1c**) as a colorless liquid (8.76 g, 48.7%).

^1^H NMR (500 MHz, CDCl_3_, TMS) (Figure S3A) δ: 2.17 (s, 1H), 3.39 (s, 3H), 3.56 (m, 2H), 3.69 (m, 2H), 4.01 (quin, 1H), 7.26 (residual internal CHCl_3_).

^13^C NMR (125 MHz, CDCl_3_, TMS) (Figure S3B) δ: 45.8, 59.0, 70.3, 70.9, 71.9, 72.2, 77.1 (CDCl_3_).

### Synthesis of 2-(Methoxymethyl)­oxirane (**2a**)

**1a** 12.96 g (104 mmol) was added dropwise to 50 wt % NaOH_aq_ 23 g, and then stirred for 2 h at ambient temperature. The resultant mixture was extracted with dichloromethane 50 mL × 3. The organic layer was collected and dried over magnesium sulfate anhydrous. The solvent was removed in vacuo to give a crude. The objective molecule was collected by distillation (74–76 °C, 190 hPa) to obtain the pure 2-(methoxymethyl)­oxirane (**2a**) as a colorless liquid (5.97 g, 31.2%).

^1^H NMR (500 MHz, CDCl_3_, TMS) (Figure S4A) δ: 2.62 (dd, 1H, ^2^ *J* = 5.0 Hz, ^3^ *J* = 2.5 Hz), 2.81 (dd, 1H, ^2^ *J* = 5.0 Hz, ^3^ *J* = 4.0 Hz), 3.16 (m, 1H), 3.33 (dd, 1H, ^2^ *J* = 11.0 Hz, ^3^ *J* = 6.0 Hz), 3.41 (s, 3H), 3.71 (dd, 1H, ^2^ *J* = 11.5 Hz, ^3^ *J* = 2.9 Hz), 7.26 (residual internal CHCl_3_).

^13^C NMR (125 MHz, CDCl_3_, TMS) (Figure S4B) δ: 44.2, 50.1, 59.3, 73.2, 77.1 (CDCl_3_).

### Synthesis of 2-(Ethoxymethyl)­oxirane (**2b**)

This molecule was synthesized by the same method with **2a**. **1b** 8.28 g (59.8 mmol) was added dropwise to 50 wt % NaOH_aq_ 20 g, and then stirred for 2 h at ambient temperature. The resultant mixture was extracted with dichloromethane 50 mL × 3. The organic layer was collected and dried over magnesium sulfate anhydrous. The solvent was removed in vacuo to give a crude. The objective molecule was collected by distillation (49–51 °C, 21 hPa) to obtain the pure 2-(ethoxymethyl)­oxirane (**2b**) as a colorless liquid (3.44 g, 56.3%).

^1^H NMR (500 MHz, CDCl_3_, TMS) (Figure S5A) δ: 1.23 (t, 3H, ^3^ *J* = 6.9), 2.62 (dd, 1H, ^2^ *J* = 5.5 Hz, ^3^ *J* = 2.5 Hz), 2.81 (dd, 1H, ^2^ *J* = 5.2 Hz, ^3^ *J* = 4.2 Hz), 3.16 (*m*, 1H), 3.39 (dd, 1H, ^2^ *J* = 11.5 Hz, ^3^ *J* = 5.5 Hz), 3.51–3.62 (m, 2H), 3.72 (dd, 1H, ^2^ *J* = 11.5 Hz, ^3^ *J* = 2.9 Hz), 7.26 (residual internal CHCl_3_).

^13^C NMR (125 MHz, CDCl_3_, TMS) (Figure S5B) δ: 15.2, 44.4, 50.9, 66.9, 71.2, 77.1 (CDCl_3_).

### Synthesis of 2-((2-Methoxyethoxy)­methyl)­oxirane (**2c**)

This molecule was synthesized by the same method with **2a**. **1c** 8.76 g (52.0 mmol) was added dropwise to 50 wt % NaOH_aq_ 20 g, and then stirred for 2 h at ambient temperature. The resultant mixture was extracted with dichloromethane 50 mL × 3. The organic layer was collected and dried over magnesium sulfate anhydrous. The solvent was removed in vacuo to give a crude. The objective molecule was collected by distillation (67–69 °C, 3 hPa) to obtain the pure 2-((2-methoxyethoxy)­methyl)­oxirane (**2c**) as a colorless liquid (5.66 g, 82.4%).

^1^H NMR (500 MHz, CDCl_3_, TMS) (Figure S6A) δ: 2.60 (dd, 1H, ^2^ *J* = 5.1 Hz, ^3^ *J* = 2.5 Hz), 2.79 (dd, 1H, ^2^ *J* = 5.0 Hz, ^3^ *J* = 3.9 Hz), 3.18 (m, 2H), 3.40 (s, 3H), 3.44 (dd, 1H, ^2^ *J* = 11.8 Hz, ^3^ *J* = 5.5 Hz), 3.57 (m, 2H), 3.63–3.73 (m, 2H), 3.80 (dd, 1H, ^2^ *J* = 11.5 Hz, ^3^ *J* = 2.9 Hz), 7.26 (residual internal CHCl_3_).

^13^C NMR (125 MHz, CDCl_3_, TMS) (Figure S6B) δ: 44.3, 50.8, 59.1, 70.7, 71.9, 72.1, 77.1 (CDCl_3_).

### Synthesis of 2-((Benzyloxy)­methyl)­oxirane (**2d**)

Epichlorohydrin 5.00 g (54.0 mmol) and TBAB 0.356 g (1.10 mmol) were dissolved in 50 wt % NaOH_aq_ 9.3 mL. Benzyl alcohol 5.84 g (54.0 mmol) was slowly added to the mixture solution over 30 min. After reaction, the mixture was added cold water 70 mL and extracted with ethyl acetate 50 mL × 3. The organic layer was collected and dried over magnesium sulfate anhydrous. The solvent was removed in vacuo to give a crude. The crude was passed through a silica gel column using hexane/diethyl ether (v/v = 4/1) as an eluent (*R*_f_ = 0.42). The obtained solution was concentrated to give the pure 2-((benzyloxy)­methyl)­oxirane (**2d**) as a colorless liquid (2.81 g, 31.4%).

^1^H NMR (500 MHz, CDCl_3_, TMS) (Figure S7A) δ: 2.62 (dd, 1H, ^2^ *J* = 5.0 Hz, ^3^ *J* = 2.0 Hz), 2.80 (dd, 1H, ^2^ *J* = 4.9 Hz, ^3^ *J* = 4.3 Hz), 3.19 (m, 1H), 3.44 (dd, 1H, ^2^ *J* = 11.5 Hz, ^3^ *J* = 5.5 Hz), 3.77 (dd, 1H, ^2^ *J* = 11.5 Hz, ^3^ *J* = 2.5 Hz), 4.59 (m, 2H), 7.26 (residual internal CHCl_3_), 7.27–7.35 (m, 5H).

^13^C NMR (125 MHz, CDCl_3_, TMS) (Figure S7B) δ: 44.4, 51.0, 70.9, 73.4, 77.1 (CDCl_3_), 127.9, 128.5, 138.0.

### Synthesis of 3-Methoxypropane-1,2-diol (**3a**)

**2a** 15.90 g (181 mmol) was dissolved into 3% TFA_aq_ 50 mL and stirred for 12 h at ambient temperature. The mixture was concentrated in vacuo. The objective molecule was collected by distillation (97–99 °C, 3 hPa) to obtain the pure 3-methoxypropane-1,2-diol (**3a**) as a colorless liquid (10.54 g, 55.0%).

^1^H NMR (500 MHz, CDCl_3_, TMS) (Figure S8A) δ: 2.41 (s, 2H), 3.40 (s, 3H), 3.46–3.52 (m, 2H), 3.68 (m, 2H), 3.88 (m, 1H), 7.26 (residual internal CHCl_3_).

^13^C NMR (125 MHz, CDCl_3_, TMS) (Figure S8B) δ: 59.4, 64.2, 70.5, 74.4, 77.1 (CDCl_3_).

### Synthesis of 3-Ethoxypropane-1,2-diol (**3b**)

This molecule was synthesized by the same method with **2a**. **2b** 3.44 g (33.7 mmol) was dissolved into 3% TFA_aq_ 10 mL and stirred for 12 h at ambient temperature. The mixture was concentrated in vacuo. The objective molecule was collected by distillation (100–102 °C, 3 hPa) to obtain the pure 3-ethoxypropane-1,2-diol (**3b**) as a colorless liquid (4.20 g, 95.4%).

^1^H NMR (500 MHz, CDCl_3_, TMS) (Figure S9A) δ: 1.22 (t, 3H, ^3^ *J* = 6.9 Hz), 2.63 (s, 2H), 3.50–3.57 (m, 4H), 3.70 (m, 2H), 3.86–3.92 (m, 1H), 7.26 (residual internal CHCl_3_).

^13^C NMR (125 MHz, CDCl_3_, TMS) (Figure S9B) δ: 15.1, 64.3, 67.1, 70.4, 72.2, 77.1 (CDCl_3_).

### Synthesis of 3-(2-Methoxyethoxy)­propane-1,2-diol (**3c**)

This molecule was synthesized by the same method with **2a**. **2c** 2.83 g (21.4 mmol) was dissolved into 3% TFA_aq_ 10 mL and stirred for 12 h at ambient temperature. The mixture was concentrated in vacuo. The objective molecule was collected by distillation (114–116 °C, 2 hPa) to obtain a colorless liquid of 3-(2-methoxyethoxy)­propane-1,2-diol (**3c**) with impurities (3.17 g, 98.8%). The compound was used for the next synthesis without further purification.

^1^H NMR (500 MHz, CDCl_3_, TMS) (Figure S10A) δ: 2.31 (s, 1H), 3.39 (s, 3H), 3.50–3.60 (m, 2H), 3.62–3.74 (m, 6H), 3.86–3.92 (m, 1H), 7.26 (residual internal CHCl_3_).

^13^C NMR (125 MHz, CDCl_3_, TMS) (Figure S10B) δ: 59.1, 64.0, 70.7, 70.8, 72.0, 73.1, 77.1 (CDCl_3_).

### Synthesis of 3-(Benzyloxy)­propane-1,2-diol (**3d**)

**2d** 2.81 g (17.1 mmol) and 1% H_2_SO_4aq_ 35 mL mixed and stirred for 12 h at ambient temperature. The mixture was neutralized with potassium carbonate and extracted with ethyl acetate 75 mL × 3. The organic layer was collected and dried over magnesium sulfate anhydrous. The solvent was removed in vacuo to give the pure 3-(benzylozy)­propane-1,2-diol (**3d**) as a colorless liquid (2.59 g, 83.1%).

^1^H NMR (500 MHz, CDCl_3_, TMS) (Figure S11A) δ: 2.08 (s, 2H), 3.54–3.61 (m, 2H), 3.68 (m, 2H), 3.88–3.92 (m, 1H), 4.56 (m, 2H), 7.26 (residual internal CHCl_3_), 7.29–7.38 (*m*, 5H).

^13^C NMR (125 MHz, CDCl_3_, TMS) (Figure S11B) δ: 64.3, 70.7, 71.9, 73.7, 77.1 (CDCl_3_), 127.8, 128.6, 137.7.

### Synthesis of 2-(Bromomethyl)-4-(methoxymethyl)-1,3-dioxolane (**4a**)

**3a** 11.51 g (109 mmol), bromoacetaldehyde diethyl acetal 21.38 g (109 mmol), and Amberlyst 15­(H) 3.00 g were mixed and stirred for 20 h at 85 °C. The mixture was concentrated in vacuo. The objective molecule was collected by distillation (90–92 °C, 2 hPa) to obtain the pure 2-(bromomethyl)-4-(methoxymethyl)-1,3-dioxolane (**4a**) as a colorless liquid (14.40 g, 62.9%).

^1^H NMR (500 MHz, CDCl_3_, TMS) (Figure S12A) δ: 3.36–3.44 (m, 2H), 3.40 (s, 3H), 3.46–3.55 (m, 2H), 4.29–4.41 (m, 1H), 5.15 (t, ^3^ *J* = 4.0 Hz), 5.26 (t, ^3^ *J* = 4.0 Hz), 7.26 (residual internal CHCl_3_).

^13^C NMR (125 MHz, CDCl_3_, TMS) (Figure S12B) δ: 32.2, 32.6, 59.5, 67.8, 72.6, 72.9, 75.6, 75.8, 77.1 (CDCl_3_), 102.3, 102.5.

### Synthesis of 2-(Bromomethyl)-4-(ethoxymethyl)-1,3-dioxolane (**4b**)

This molecule was synthesized by the same method with **4a**. **3b** 4.20 g (34.9 mmol), bromoacetaldehyde diethyl acetal 6.89 g (34.9 mmol), and Amberlyst 15­(H) 1.40 g were mixed and stirred for 20 h at 85 °C. The mixture was concentrated in vacuo. The objective molecule was collected by distillation (96–98 °C, 2 hPa) to obtain the pure 2-(bromomethyl)-4-(ethoxymethyl)-1,3-dioxolane (**4b**) as a colorless liquid (5.60 g, 71.1%).

^1^H NMR (500 MHz, CDCl_3_, TMS) (Figure S13A) δ: 1.21 (t, 3H, ^3^ *J* = 7.2 Hz), 3.36–3.59 (m, 6H), 3.77–4.21 (m, 2H), 4.29–4.41 (m, 1H), 5.15 (t, ^3^ *J* = 4.0 Hz), 5.26 (t, ^3^ *J* = 4.0 Hz), 7.26 (residual internal CHCl_3_).

^13^C NMR (125 MHz, CDCl_3_, TMS) (Figure S13B) δ: 15.1, 32.4, 32.7, 67.2, 68.0, 70.6, 70.9, 75.8, 76.0, 77.1 (CDCl_3_), 102.3, 102.5.

### Synthesis of 2-(Bromomethyl)-4-((2-methoxyethoxy)­methyl)-1,3-dioxolane (**4c**)

This molecule was synthesized by the same method with **4a**. **3c** 1.69 g (11.3 mmol), bromoacetaldehyde diethyl acetal 2.27 g (11.3 mmol), and Amberlyst 15­(H) 0.30 g were mixed and stirred for 20 h at 85 °C. The mixture was concentrated in vacuo. The objective molecule was collected by distillation (135–137 °C, 2 hPa) to obtain the pure 2-(bromomethyl)-4-((2-methoxyethoxy)­methyl)-1,3-dioxolane (**4c**) as a colorless liquid (0.85 g, 29.5%).

^1^H NMR (500 MHz, CDCl_3_, TMS) (Figure S14A) δ: 3.39 (s, 3H), 3.54–3.68 (m, 6H), 3.78–4.21 (m, 2H), 4.33–4.44 (m, 1H), 5.15 (t, ^3^ *J* = 4.0 Hz), 5.26 (t, ^3^ *J* = 4.0 Hz), 7.26 (residual internal CHCl_3_).

^13^C NMR (125 MHz, CDCl_3_, TMS) (Figure S14B) δ: 32.3, 32.6, 59.1, 67.9, 71.0, 71.3, 71.6, 71.8, 75.7, 75.8, 77.1 (CDCl_3_), 102.3, 102.4.

### Synthesis of 4-((Benzyloxy)­methyl)-2-(bromomethyl)-1,3-dioxolane (**4d**)

**3d** 17.87 g (98.1 mmol), bromoacetaldehyde diethyl acetal 18.63 g (99.6 mmol), and Amberlyst 15­(H) 1.11 g were mixed and stirred for 20 h at 85 °C. The mixture was concentrated in vacuo. The crude was passed through a silica gel column using hexane/diethyl ether (v/v = 4/1) as an eluent (*R*_f_ = 0.15). The obtained solution was concentrated to give the pure 4-((benzyloxy)­methyl)-2-(bromomethyl)-1,3-dioxolane (**4d**) as a colorless liquid (14.17 g, 50.6%).

^1^H NMR (500 MHz, CDCl_3_, TMS) (Figure S15A) δ: 3.36–3.45 (m, 2H), 3.57 (m, 2H), 3.79–4.20 (m, 2H), 4.32–4.44 (m, 1H), 4.48 (s, 2H), 5.15 (t, ^3^ *J* = 4.0 Hz), 5.26 (t, ^3^ *J* = 4.0 Hz), 7.26 (residual internal CHCl_3_), 7.29–7.37 (*m*, 5H).

^13^C NMR (125 MHz, CDCl_3_, TMS) (Figure S15B) δ: 32.3, 32.6, 67.9, 69.9, 70.3, 75.5, 75.7, 75.8, 77.1 (CDCl_3_), 102.3, 102.5, 127.7, 128.4, 137.7.

### Synthesis of 4-(Methoxymethyl)-2-methylene-1,3-dioxolane (**5a**)

Potassium *tert*-butoxide 3.88 g (34.6 mmol) was dissolved in THF 40 mL. **4a** 2.64 g (12.5 mmol) and TBAB 0.26 g (0.81 mmol) were dissolved in THF 50 mL, and then the mixture was added dropwise the THF solution of potassium *tert*-butoxide. The mixture was stirred for 2 h at 60 °C. After reaction, hexane 300 mL was added to the reaction mixture. The formed precipitation was removed by filtration. The filtrate was concentrated in vacuo. The objective molecule was collected by distillation (54–56 °C, 3 hPa) to obtain the pure 4-(methoxymethyl)-2-methylene-1,3-dioxolane (**5a**) as a colorless liquid (0.67 g, 41.2%).

^1^H NMR (500 MHz, CDCl_3_, TMS) (Figure 1A) δ: 3.26 (d, 2H, ^2^ *J* = 11.3 Hz), 3.42 (s, 3H), 3.54 (m, 2H), 4.02 (dd, 1H, ^2^ *J* = 11.3 Hz, ^3^ *J* = 8.0 Hz), 4.24 (dd, 1H, ^2^ *J* = 8.2 Hz, ^3^ *J* = 7.5 Hz), 4.55–5.59 (m, 1H), 7.26 (residual internal CHCl_3_).

**Figure 1:**
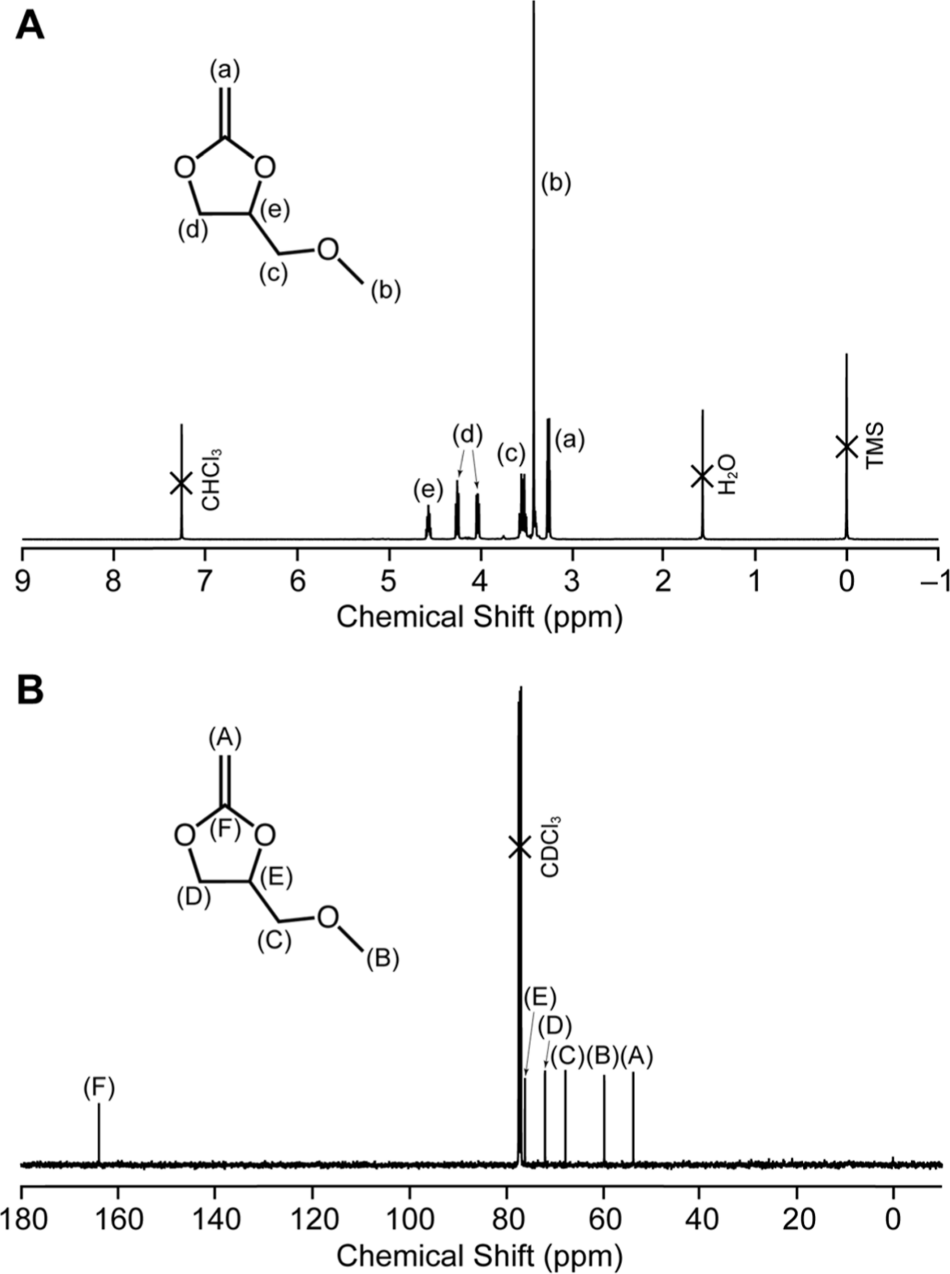
(A) ^1^H and (B) ^13^C NMR spectra of **5a** in CDCl_3_ at 25 °C.

^13^C NMR (125 MHz, CDCl_3_, TMS) (Figure 1B) δ: 53.6, 59.6, 67.6, 71.8, 76.0, 77.1 (CDCl_3_), 163.8.

HRMS (DART) *m*/*z*: [M + H]^+^ C_6_H_11_O_3_^+^ calcd for 131.0703; found, 131.0695.

### Synthesis of 4-(Ethoxymethyl)-2-methylene-1,3-dioxolane (**5b**)

This molecule was synthesized by the same method with **5a**. Potassium *tert*-butoxide 5.51 g (49.1 mmol) was dissolved in THF 70 mL. **4b** 3.67 g (16.3 mmol) and TBAB 0.37 g (1.15 mmol) were dissolved in THF 60 mL, and then the mixture was added dropwise the THF solution of potassium *tert*-butoxide. The mixture was stirred for 2 h at 60 °C. After reaction, hexane 350 mL was added to the reaction mixture. The formed precipitation was removed by filtration. The filtrate was concentrated in vacuo. The objective molecule was collected by distillation (67–69 °C, 2 hPa) to obtain the pure 4-(ethoxymethyl)-2-methylene-1,3-dioxolane (**5b**) as a colorless liquid (1.44 g, 61.3%).

^1^H NMR (500 MHz, CDCl_3_, TMS) (Figure S16A) δ: 1.21 (t, 3H, ^3^ *J* = 7.2 Hz), 3.24 (d, 2H, ^2^ *J* = 11.3 Hz), 3.51–3.62 (m, 4H), 4.02 (dd, 1H, ^2^ *J* = 8.1 Hz, ^3^ *J* = 7.0 Hz), 4.24 (dd, 1H, ^2^ *J* = 7.3 Hz, ^3^ *J* = 7.0 Hz), 4.54–4.59 (m, 1H), 7.26 (residual internal CHCl_3_).

^13^C NMR (125 MHz, CDCl_3_, TMS) (Figure S16B) δ: 15.1, 53.6, 67.2, 67.8, 71.8, 76.5, 77.1 (CDCl_3_), 163.8.

HRMS (DART) *m*/*z*: [M + H]^+^ C_7_H_13_O_3_^+^ calcd for 145.0859; found, 145.0872.

### Synthesis of 4-((2-Methoxyethoxy)­methyl)-2-methylene-1,3-dioxolane (**5c**)

This molecule was synthesized by the same method with **5a**. Potassium *tert*-butoxide 4.31 g (38.4 mmol) was dissolved in THF 60 mL. **4c** 2.87 g (11.3 mmol) and TBAB 0.28 g (0.87 mmol) were dissolved in THF 40 mL, and then the mixture was added dropwise the THF solution of potassium *tert*-butoxide. The mixture was stirred for 2 h at 60 °C. After reaction, hexane 300 mL was added to the reaction mixture. The formed precipitation was removed by filtration. The filtrate was concentrated in vacuo. The objective molecule was collected by distillation (67–69 °C, 2 hPa) to obtain the pure 4-((2-methoxyethoxy)­methyl)-2-methylene-1,3-dioxolane (**5c**) as a colorless liquid (0.67 g, 41.2%).

^1^H NMR (500 MHz, CDCl_3_, TMS) (Figure S17A) δ: 3.24 (d, 2H, ^2^ *J* = 11.3 Hz), 3.38 (s, 3H), 3.54–3.67 (m, 6H), 4.03–4.27 (m, 2H), 4.57–4.62 (m, 1H), 7.26 (residual internal CHCl_3_).

^13^C NMR (125 MHz, CDCl_3_, TMS) (Figure S17B) δ: 53.6, 59.2, 67.6, 70.6, 71.2, 71.9, 76.2, 77.1 (CDCl_3_), 163.8.

HRMS (DART) *m*/*z*: [M + H]^+^ C_8_H_15_O_4_^+^ calcd for 175.0965; found, 175.0959.

### Synthesis of 4-((Benzyloxy)­methyl)-2-methylene-1,3-dioxolane (**5d**)

This molecule was synthesized by the same method with **5a**. Potassium *tert*-butoxide 2.87 g (25.6 mmol) was dissolved in THF 30 mL. **4d** 1.82 g (6.67 mmol) and TBAB 0.28 g (0.70 mmol) were dissolved in THF 55 mL, and then the mixture was added dropwise the THF solution of potassium *tert*-butoxide. The mixture was stirred for 2 h at 60 °C. After reaction, hexane 300 mL was added to the reaction mixture. The formed precipitation was removed by filtration. The filtrate was concentrated in vacuo. The objective molecule was collected by distillation (130–132 °C, <1 hPa) to obtain the pure 4-((benzyloxy)­methyl)-2-methylene-1,3-dioxolane (**5d**) as a colorless liquid (0.95 g, 68.8%).

^1^H NMR (500 MHz, CDCl_3_, TMS) (Figure S18A) δ: 3.25 (d, 2H, ^2^ *J* = 11.3 Hz), 3.60 (m, 2H), 4.02 (dd, 1H, ^2^ *J* = 8.1 Hz, ^3^ *J* = 7.0 Hz), 4.24 (dd, 1H, ^2^ *J* = 7.3 Hz, ^3^ *J* = 7.0 Hz), 4.59 (m, 3H), 7.26 (residual internal CHCl_3_), 7.29–7.38 (*m*, 5H).

^13^C NMR (125 MHz, CDCl_3_, TMS) (Figure S18B) δ: 53.6, 67.8, 69.2, 73.7, 76.1, 77.1 (CDCl_3_), 127.7, 128.1, 128.6, 137.4, 163.9.

HRMS (DART) *m*/*z*: [M + H]^+^ C_12_H_15_O_3_^+^ calcd for 207.1016; found, 207.0985.

### Synthesis of 2-Bromomethyl-1,3-dioxolane

2-Bromomethyl-1,3-dioxolane was synthesized according to the method described in the literature.^18^

^1^H NMR (500 MHz, CDCl_3_, TMS) δ: 3.38 (d, 2H, *J* = 4.0 Hz), 3.94 (m, 2H), 4.04 (m, 2H), 5.13 (t, 1H, *J* = 4.0 Hz).

### Synthesis of 2-Methylene-1,3-dioxolane (**C5**)

**C5** was synthesized according to the method described in the literature.^18^

^1^H NMR (500 MHz, CDCl_3_, TMS) δ: 3.25 (s, 2H), 4.21 (s, 4H).

### General Procedure for the Radical Polymerization of CKAs

CKA (22 mmol) and radical initiator (1/50 equiv) were dissolved in *o*-dichlorobenzene. AIBN, VAm-110, DTBP were employed as initiators for polymerizations conducted at 60, 100, and 140 °C, respectively. The solutions were transferred to test tubes and deoxygenated using the freeze–pump–thaw cycle. The reaction mixtures were then placed in a preheated oil bath at either 60, 100, or 140 °C. After polymerization for 24 h, the mixtures were cooled to ambient temperature. The resulting polymers were isolated and purified by reprecipitation from a THF/hexane system. The obtained polymers exhibited moderate polarity. They were insoluble in highly polar protic solvents such as water and in nonpolar solvents such as hexane. In contrast, they dissolved readily in polar aprotic solvents (e.g., THF, *N*,*N*-dimethylformamide), alcoholic solvents (e.g., methanol), and moderately hydrophobic solvents (e.g., chloroform, ethyl acetate, and toluene). The resulting polymers were isolated and purified by reprecipitation from a THF/hexane system. The monomer conversion was determined by ^1^H NMR spectroscopy before purification based on the decrease in the integration of the peak at approximately 3.25 ppm. Under these polymerization conditions, all reactions achieved conversions exceeding 99%.

### Simulation Study by Density Functional Theory Calculations

DFT calculations were performed using Firefly 8.2.0.^35^ The molecular modeling for each compound was conducted using Winmostar V11. The B3LYP functional was employed for the calculations, with a 6-31G* basis set. After optimizing the structures to their lowest energy conformations, single-point energy calculations were conducted to determine the total electronic energy. Additionally, vibrational frequency analysis was performed to obtain the enthalpy (*H*), entropy (*S*), and Gibbs free energy (*G*) of the initial states. The transition states were identified as saddle points with only one imaginary frequency, and their enthalpy (*H* ^‡^), entropy (*S* ^‡^), and Gibbs free energy (*G* ^‡^) were also calculated. These differentiations were activation enthalpy (Δ*H* ^‡^), activation entropy (Δ*S* ^‡^), and activation Gibbs free energy (Δ*G* ^‡^). The reaction rate constant (*k*) for each reaction step was calculated by Eyring equation shown below.

Monomolecular reaction

1 $$k=\frac{k_{B}T}{h}e^{-\Delta G^{‡}/RT}$$

Bimolecular reaction

2 $$k=\frac{k_{B}T}{h}\cdot \frac{1}{c_{0}}e^{-\Delta G^{‡}/RT}$$

where *k*_B_ is Boltzmann constant, *h* is Planck constant, *R* is gas constant, *T* is absolute temperature, *c*_0_ is standard concentration. The 3D structures and their Cartesian coordinates for the transition states were shown in the Supporting Information.

## Results

### Synthesis of 5-Membered Cyclic Ketene Acetals Bearing Alkoxy Methyl Groups

4-Position modified CKAs with alkoxymethyl groups (**5a–d**) were synthesized from epichlorohydrin as a starting material (Scheme 2). Epichlorohydrin is attractive precursor because it can be derived from glycerol which is a major byproduct in a biodiesel industry. Initially, epichlorohydrin was reacted with the corresponding alcohols (ROH, R = CH_3_, CH_2_CH_3_, CH_2_CH_2_OCH_3_) in the presence of BF_3_–Et_2_O to obtain 1-chloro-3-alkoxy-2-propanols (**1a–c**). Oxiranes (**2a–c**) were then synthesized by treating **1a–c** with 50 wt % NaOH_aq_. Benzoyloxymethyl group-modified oxirane (**2d**) was directly synthesized from epichlorohydrin by reacting it with benzyl alcohol in 50 wt % NaOH_aq_ in the presence of TBAB. The diol compounds (**3a–d**) were obtained by acid hydrolysis of **2a–d** and then reacted with bromoacetaldehyde diethyl acetal in the presence of acid catalyst under reflux condition to give cyclic bromo acetal-derivatives (**4a–d**). The chemical structures of them were determined by ^1^H and ^13^C NMR spectroscopies (Figure S1–S15). **4a–d** were treated with *t*BuOK in the presence of TBAB to synthesize **5a–d**. The objective compounds were isolated by distillation. The CKAs are unstable in a protic environment because delocalization of charges from two oxygens in the acetal to C=C double bond ensures that carbon in the β position is a strong nucleophile.^9^ Surfaces of glassware composed of SiO_2_ are weakly acidic, which induces the spontaneous cationic polymerization of the CKAs. Therefore, the distillation of **5a–d** were carried out using apparatus precoated with chlorotrimethylsilane.^18^ The chemical structures of **5a–d** were identified by ^1^H and ^13^C NMR spectroscopies. As representative results, ^1^H and ^13^C NMR spectra of **5a** are shown in Figure 1. The NMR results of the other CKAs are shown in Figures S16–S18.

**Scheme 2:**
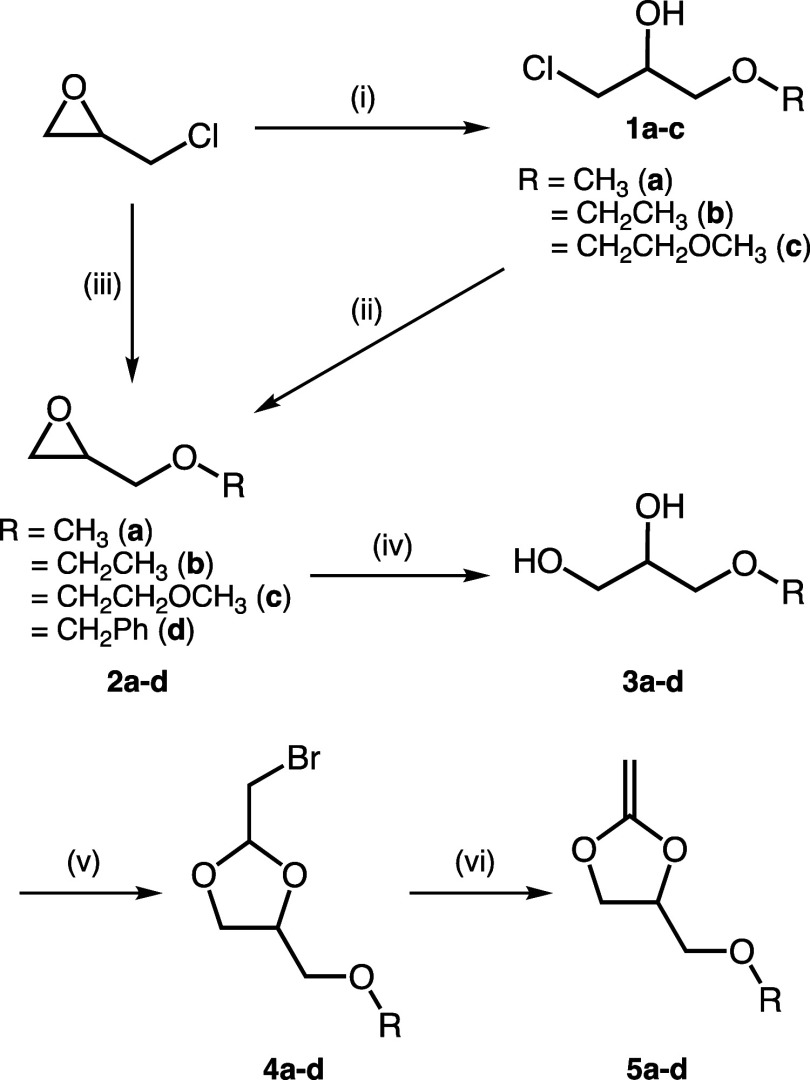
Synthetic Route for 5-Membered CKAs Bearing Alkoxymethyl Groups. (i) ROH (R = CH_3_, CH_2_CH_3_, CH_2_CH_2_OCH_3_), BF_3_–Et_2_O. (ii) 50 wt % NaOH_aq_. (iii) PhCH_2_OH, TBAB, 50 wt % NaOH_aq_. (iv) 3% TFA_aq_ (for **2a–c**). 1% H_2_SO_4aq_ (for **2d**). (v) Bromoacetaldehyde Diethyl Acetal, Amberlyst 15­(H). (vi) *t*BuOK, TBAB

### Radical Polymerization of **5a** and Analysis of Chemical Structure of Its Polymer

Initially, radical polymerization of **5a** was carried at 140 °C in *o*-dichlorobenzene using DTBP as an initiator ([**5a**] = 6 M). DTBP was selected due to its half-life of approximately 1 h at 140 °C, which ensures sufficient radical generation during the early stage of polymerization. Since the objective of this study was to analyze polymer composition at high monomer conversion, rather than to control molecular weight or dispersity, DTBP was deemed appropriate for this purpose. After polymerization for 20 h, the monomer conversion was over 99% calculated by ^1^H NMR spectroscopy. The resultant polymer was purified by reprecipitation method using the THF/hexane system. The number-average molecular weight (*M*_n_), weight-average molecular weight (*M*_w_) and dispersity (*D̵*) were evaluated by size-exclusion chromatography (SEC) (Figure 2A). The chromatogram exhibits broad distribution toward a region of low molecular wight, and the molecular weights are relatively small (*M*_n_ = 3,100, *M*_w_ = 5,200, *D̵* = 1.68). Figure 2B,C show ^1^H and ^13^C NMR spectra of the polymer, respectively. The spectra suggested that the resultant polymer contains several components formed through a complex polymerization process.

**Figure 2:**
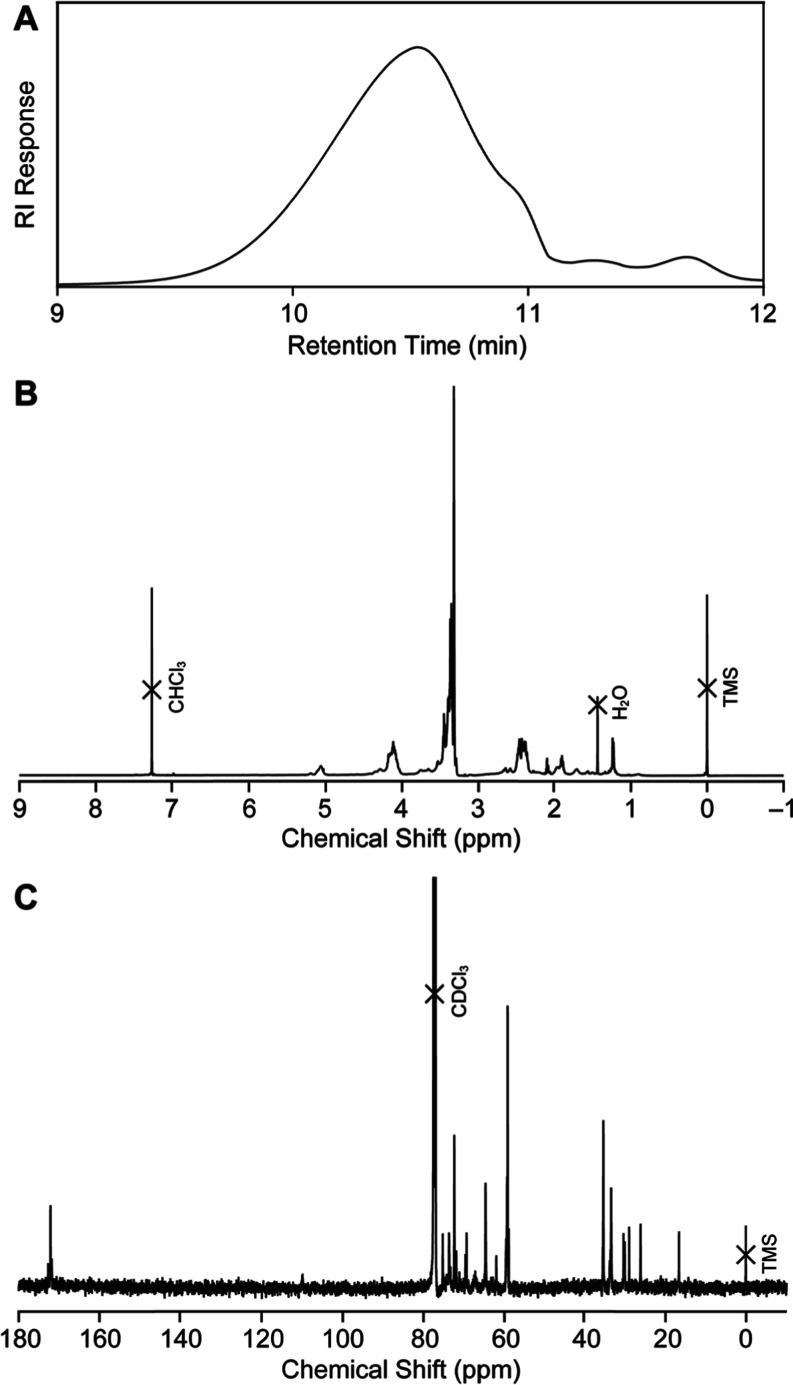
(A) SEC chromatogram of the **5a** polymer in THF at 40 °C. (B) ^1^H and (C) ^13^C NMR spectra of the polymer in CDCl_3_ at 25 °C.

During the polymerization of **C5-COR**, five kinds of growing radicals are generated (Scheme 3). The ring-closed acetalyl radical (*c* structure) forms alkyl radicals by ring-opening via β-scission at 3-position oxygen (3O)/4-position carbon (4C) (*o*1 structure) and 1-position oxygen (1O)/5-position carbon (5C) (*o*2 structure). By 1,5-H transfer reaction (backbiting reaction), these radicals rearrange to acrylate-type radicals (*b*1 and *b*2 structures, respectively). Therefore, the resultant **5a** polymer is expected to contain 25 kinds of diad structures (Figures S19). DEPT90, DEPT135, ^1^H–^1^H COSY, ^1^H–^1^H TOCSY, ^1^H–^13^C HMQC, and ^1^H–^13^C HMBC measurements were performed to determine the chemical structure of the polymer. The structural motifs *c*, *o*1, *o*2, *b*1, and *b*2, along with 15 distinct diad sequences, were successfully assigned.

**Scheme 3:**
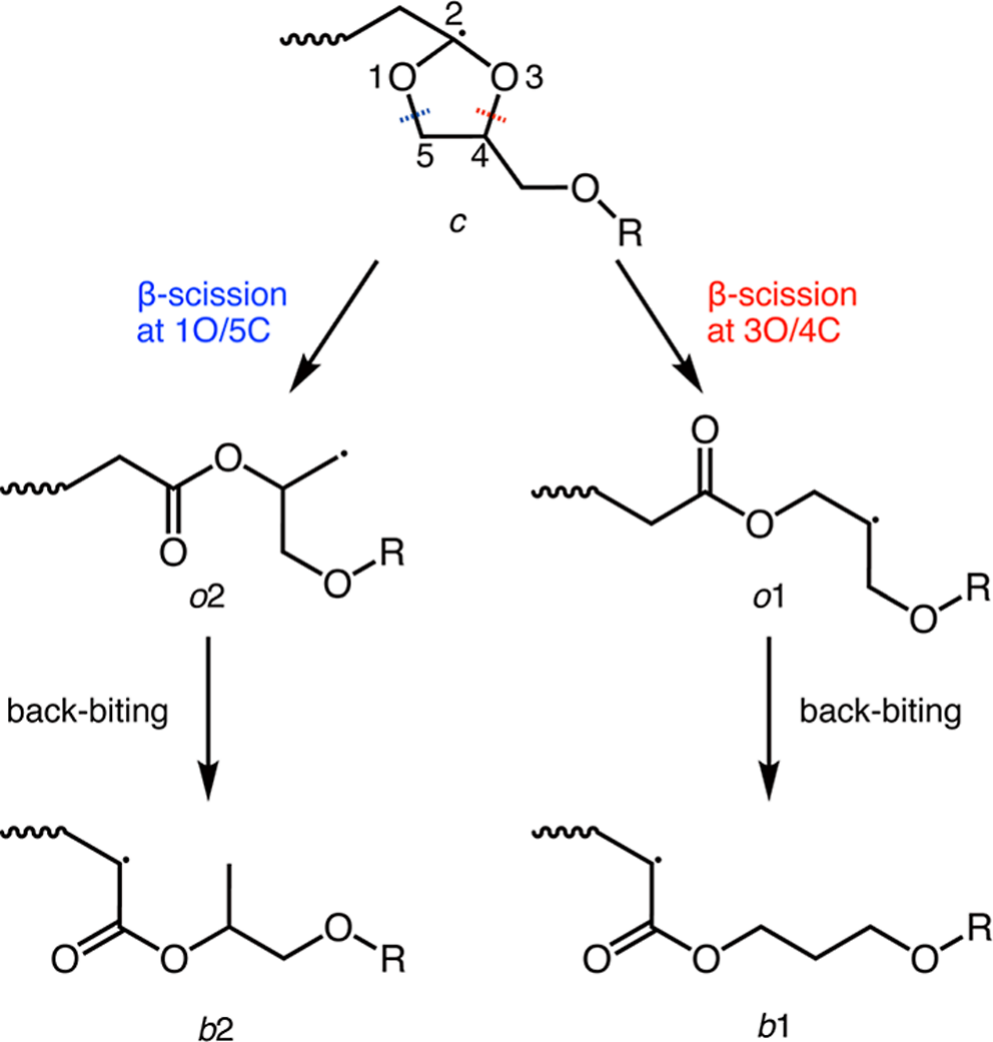
Rearrangement of Growing Radical Generated in the Polymerization of **C5-COR**

Detailed analyses including the assignment of the polymer structures are described in the Supporting Information (Figures S20–S23). Importantly, the **5a** polymer exhibited a carbon signal at approximately 170 ppm, indicative of polyester structure formation (Figure 2C). The ratio of *c*, *o*1, *o*2, *b*1, and *b*2 structures in the **5a** polymer was found to depend on the polymer conditions including monomer concentration and temperature. These points are discussed later.

Its biodegradability, evaluated according to the OECD 301F manometric respirometry method, reached approximately 20% after 28 days (Figure S24, black circle), as calculated from the ratio of BOD to ThOD. For comparison, cellulose—a widely recognized biodegradable polymer—underwent around 40% degradation under the same test conditions (Figure S24, open circle). Notably, the **5a** polymer achieved nearly half the degradation level of cellulose in this standardized assay, strongly supporting the presence of hydrolytically cleavable ester linkages in its main chain. However, it should be noted that the presence of ring-retaining units—i.e., structures resulting from incomplete ring-opening—may lead to the formation of nonhydrolyzable oligomers upon degradation.

### Polymerization of **5a** and C5 under Various Conditions

To examine an effect of modification of alkoxymethyl group at 4-position on a polymerization behavior of CKA, polymerizations of **5a** were carried out under various monomer concentrations (7.7, 6, 4, 2.4, 1.2, and 0.5 M) and temperatures (140, 100, and 60 °C) in *o*-dichlorobenzene. By ^1^H NMR analysis of the resultant polymers without purification, the following structural ratios were determined: the ratio of ring-retaining structures (*R*_c_), the ratio of ring-opening structures formed via *o*1 β-scissions including backbiting reactions (*R*_*op*1_), the ratio of ring-opening structures formed via *o*2 β-scissions including backbiting reactions (*R*_*op*2_), the ratio of ester structures formed via *o*1 β-scissions (*R*_*o*1_), the ratio of ester structures formed via *o*2 β-scissions (*R*_*o*2_), the ratio of backbitten structures formed via *o*1 β-scissions (*R*_*b*1_), and ratio of backbitten structures formed via *o*2 β-scissions (*R*_b2_). Note that the monomer concentration changes continuously over time; therefore, the resulting polymer exhibits an average ratio of units ( R̅ ) that reflects structures formed across the range of monomer concentrations between the initial and final states. The R̅ values are summarized in Table 1, and the composition of each structural unit as a function of the initial monomer concentration is plotted in Figure 3. For comparison, nonsubstituted 5-membered CKA (**C5**) was also polymerized, following a modified procedure based on previous literature reports.^16,18^ In the same manner as for **5a**, the chemical structures of polymers obtained from **C5** polymerization were analyzed (Table 2 and Figure 4). It should be noted that, in the case of **C5**, β-scissions occurring at the 3*O*/4C and 1*O*/5C positions were not distinguished. The assignments of chemical structures were performed based on the literatures.^18^ The effects of monomer concentration and polymerization temperature on the structural composition differed markedly between the **5a** and **C5** polymerizations. These differences, as shown in Figures 3 and 4, highlight the significant impact of 4-position substitution on the polymerization pathways and the resulting polymer structures. The details of these trends are discussed in the following section.

**Table 1:** Summary of the Polymerization of **5a** in This Study

[M]^a^	temp. (°C)	®*R*_*c*_ ^b^× 10^2^	®*R*_*op*1_ ^b^× 10^2^	®*R*_*op*1_ ^b^× 10^2^	®*R*_*o*1_ ^b^× 10^2^	®*R*_*o*2_ ^b^× 10^2^	®*R*_*b*1_ ^b^×10^2^	®*R*_*b*2_ ^b^× 10^2^
7.7	60	9.9	68.5	21.6	67.6	19.5	0.9	2.1
6.0	60	8.7	68.3	23.1	67.8	20.9	0.5	2.2
4.0	60	8.3	70.4	21.3	69.4	19.2	1.0	2.1
2.4	60	8.2	71.3	20.5	69.2	18.5	2.1	2.0
1.2	60	7.6	72.6	19.8	69.8	17.5	2.8	2.3
0.5	60	3.3	73.7	23.0	63.9	16.5	9.8	6.5
7.7	100	3.3	70.7	26.0	68.8	19.1	1.9	6.9
6.0	100	5.1	68.6	26.3	66.9	19.4	1.7	6.9
4.0	100	3.4	71.6	25.0	67.6	18.3	4.0	6.7
2.4	100	2.9	71.1	26.0	67.8	18.8	3.3	7.2
1.2	100	2.8	74.0	23.2	67.9	16.2	6.1	7.0
0.5	100	1.7	70.8	27.5	54.5	14.2	16.3	13.3
7.7	140	1.6	69.7	28.7	60.8	15.0	8.9	13.7
6.0	140	0.6	73.1	26.3	58.6	10.5	14.5	15.8
4.0	140	1.1	72.2	26.7	55.8	10.5	16.4	16.2
2.4	140	0.6	74.3	25.1	57.1	8.7	17.2	16.4
1.2	140	1.0	71.3	27.7	54.6	9.1	16.7	18.6
0.5	140	1.0	74.1	24.9	42.0	5.6	32.1	19.3

a The polymerizations were carried out in *o*-dichlorobenzene.

b Calculated from ^1^H NMR analyses.

**Figure 3:**
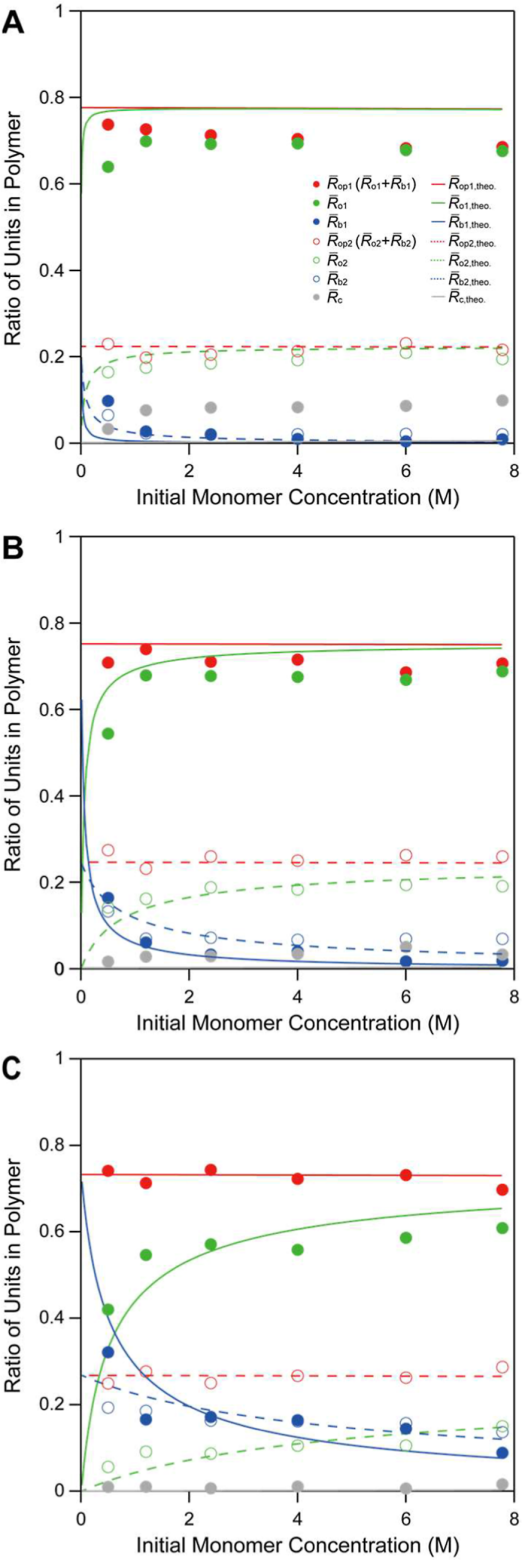
Plots of average ratio of units in the polymers obtained by polymerization of **5a** at various initial monomer concentrations. The polymerizations were conducted at (A) 60 °C, (B) 100 °C, and (C) 140 °C. The plots show ${\mathrm{R̅}}_{c}$ (gray circle), ${\mathrm{R̅}}_{op1}$ (red circle), ${\mathrm{R̅}}_{op2}$ (open red circle), ${\mathrm{R̅}}_{o1}$ (green circle), ${\mathrm{R̅}}_{o2}$ (open green circle), ${\mathrm{R̅}}_{b1}$ (blue circle), ${\mathrm{R̅}}_{b2}$ (open blue circle). The theoretical values are shown as solid lines for filled circles and dashed line for open circle with the corresponding colors.

**Table 2:** Summary of the Polymerization of **C5** in This Study

[M]^a^	temp. (°C)	®*R*_*c*_ ^b^× 10^2^	®*R*_*op*_ ^b^× 10^2^	®*R*_*o*_ ^b^× 10^2^	*®*R**_*b*_ ^b^× 10^2^
7.7	60	13.9	86.1	76.1	10.0
6.0	60	13.2	86.8	75.9	10.9
4.0	60	12.7	87.3	72.3	15.0
2.4	60	11.6	88.4	68.9	19.5
1.2	60	7.5	92.5	71.1	21.4
0.5	60	8.6	91.4	63.9	27.5
7.7	100	11.6	88.4	73.7	14.7
6.0	100	12.7	87.3	71.0	16.3
4.0	100	13.6	86.4	63.6	22.8
2.4	100	11.1	88.9	57.2	31.7
1.2	100	7.3	92.7	45.8	46.9
0.5	100	5.1	94.9	30.5	64.4
7.7	140	2.6	97.4	58.7	38.7
6.0	140	3.9	96.1	53.6	42.5
4.0	140	2.9	97.1	48.7	48.4
2.4	140	2.2	97.8	34.1	63.7
1.2	140	4.4	95.6	18.5	77.1
0.5	140	3.4	96.6	11.9	84.7

a The polymerizations were carried out in *o*-dichlorobenzene.

b Calculated from ^1^H NMR analyses.

**Figure 4:**
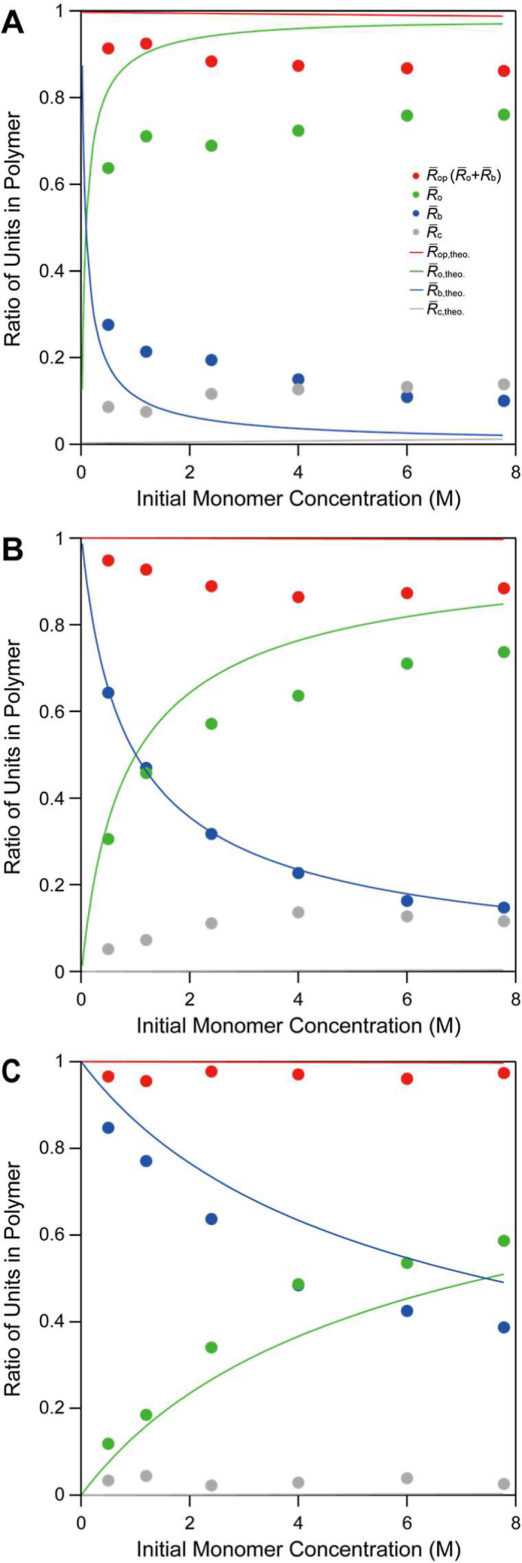
Plots of average ratio of units in the polymers obtained by polymerization of **C5** at various initial monomer concentrations. The polymerizations were conducted at (A) 60 °C, (B) 100 °C, and (C) 140 °C. The plots show ${\mathrm{R̅}}_{c}$ (gray circle), ${\mathrm{R̅}}_{op}$ (red circle), ${\mathrm{R̅}}_{o}$ (green circle), ${\mathrm{R̅}}_{b}$ (blue circle). The theoretical values are shown as solid lines with the corresponding colors.

### Computational Study for the Polymerization Behaviors of **5a** and **C5**

We determined rate constants in the propagation process of **5a** polymerizations at standard state by density functional theory (DFT) calculation at the B3LYP/6-31G* level. Tardy et al. reported that B3LYP/6-31G* level is one of the most suitable choices for evaluating thermodynamic quantities of CKAs. In this simulation, the model radicals were assumed to be initiated by a methyl radical. The propagation reaction of **5a** (Scheme 4) can be classified into nine types of reactions as follow: (i) addition of the *c* radical to **5a**, (ii) β-scission of the *c* radical at 3*O*/4C position to form *o*1 radical, (iii) β-scission of the *c* radical at 1*O*/5C position to form *o*2 radical, (iv) addition of the *o*1 radical to **5a**, (v) addition of the *o*2 radical to **5a**, (vi) backbiting of the *o*1 radical to form *b*1 radical, (vii) backbiting of the *o*2 radical to form *b*2 radical, (viii) addition of the *b*1 radical to **5a**, (ix) addition of the *b*2 radical to **5a**. The rate constants for each reaction are denoted as follow: *k*_*c*–*c*_ (i), *k*_*o*1_ (ii), *k*_*o*2_ (iii), *k*_*o*1–*c*_ (iv), *k*_*o*2–*c*_ (v), *k*_*b*1_ (vi), *k*_*b*2_ (vii), *k*_*b*1–*c*_ (viii), and *k*_*b*2–*c*_ (ix). For these reactions, activation enthalpy (Δ*H* ^‡^), entropy (Δ*S* ^‡^), and Gibbs free energy (Δ*G* ^‡^) were estimated, and corresponding rate constants were calculated using Eyring equation. The results of these DFT calculations are summarized in Table 3. To enable direct comparison, the same computational approach was applied to the nonsubstituted **C5**. In contrast to **5a**, the propagation pathway of **C5** (Scheme 5) is simpler and consists of five distinct reactions: (x) addition of the *c* radical to **C5**, (xi) β-scission of the *c* to form *o* radical (without distinction between 3*O*/4C and 1*O*/5C positions). (xii) addition of the *o* radical to **C5**, (xiii) backbiting of the *o* radical to form *b* radical, (xiv) addition of the *b* radical to **C5**. The corresponding rate constants are denoted as *k*_*c*–*c*_ (x), *k*_*o*_ (xi), *k*_*o*–*c*_ (xii), *k*_*b*_ (xiii), and *k*_*b*–*c*_ (xiv), respectively. The calculated thermodynamic parameters and rate constants for **C5** are summarized in Table 4. These calculations revealed distinct differences in activation parameters and rate constants between **5a** and **C5**, reflecting the influence of 4-position substitution on the propagation and scission pathways. A detailed discussion of these results is provided in the following section.

**Scheme 4:**
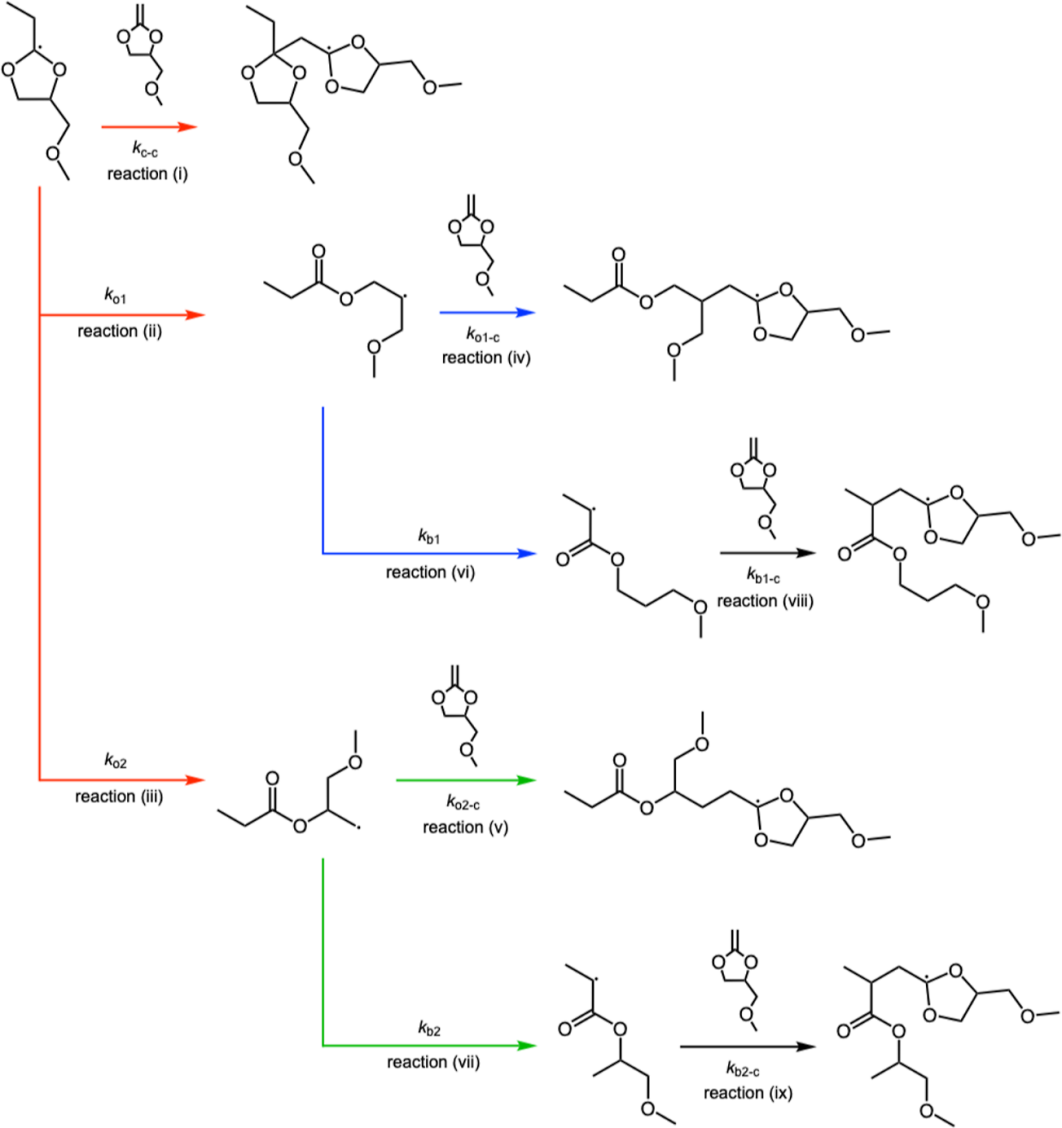
Reaction Scheme Illustrating the Possible Pathways for Propagation of **5a** polymerization

**Table 3:** Summary of DFT Calculations for the Propagation of **5a** Using the B3LYP/6-31G* Method^a^

reactions	Δ*H* ^‡^ (kJ/mol)	Δ*S* ^‡^ (kJ/(mol K))	Δ*G* ^‡^ (kJ/mol)	*k* (L/(mol s))^b^
(i) *c*–*c*	24.2	–0.152	69.6	3.94
(ii) *o*1	57.9	3.05 × 10^–3^	56.9	6.49 × 10^2^ (/s)^c^
(iii) *o*2	61.3	3.05 × 10^–3^	60.4	1.62 × 10^2^ (/s)^c^
(iv) *o*1–c	12.4	–0.170	62.9	58.3
(v) *o*2–c	10.3	–0.178	63.3	50.9
(vi) *b*1	89.3	5.04 × 10^–3^	87.8	2.60 × 10^–3^ (/s)^c^
(vii) *b*2	72.4	–1.98 × 10^–2^	78.3	0.118 (/s)^c^
(viii) *b*1–c	23.3	–0.183	77.9	0.142
(xi) *b*2–c	28.7	–0.190	85.5	6.63 × 10^–3^

a The simulations were performed at 298.15 K (25 °C). Details of the reaction pathways are shown in Scheme 4.

b Calculated from Eyring equation for bimolecular reaction: *k* = (*k*_B_·*T*/*h*)·(1/*c*_0_) exp­(−Δ*G* ^‡^/*RT*).

c Calculated from Eyring equation for monomolecular reaction: *k* = (*k*_B_·*T*/*h*) exp­(−Δ*G* ^‡^/*RT*).*k*_B_: Boltzmann constant. *h*: Planck constant. *R*: gas constant. *T*: absolute temperature. *c*_0_: standard concentration.

**Scheme 5:**
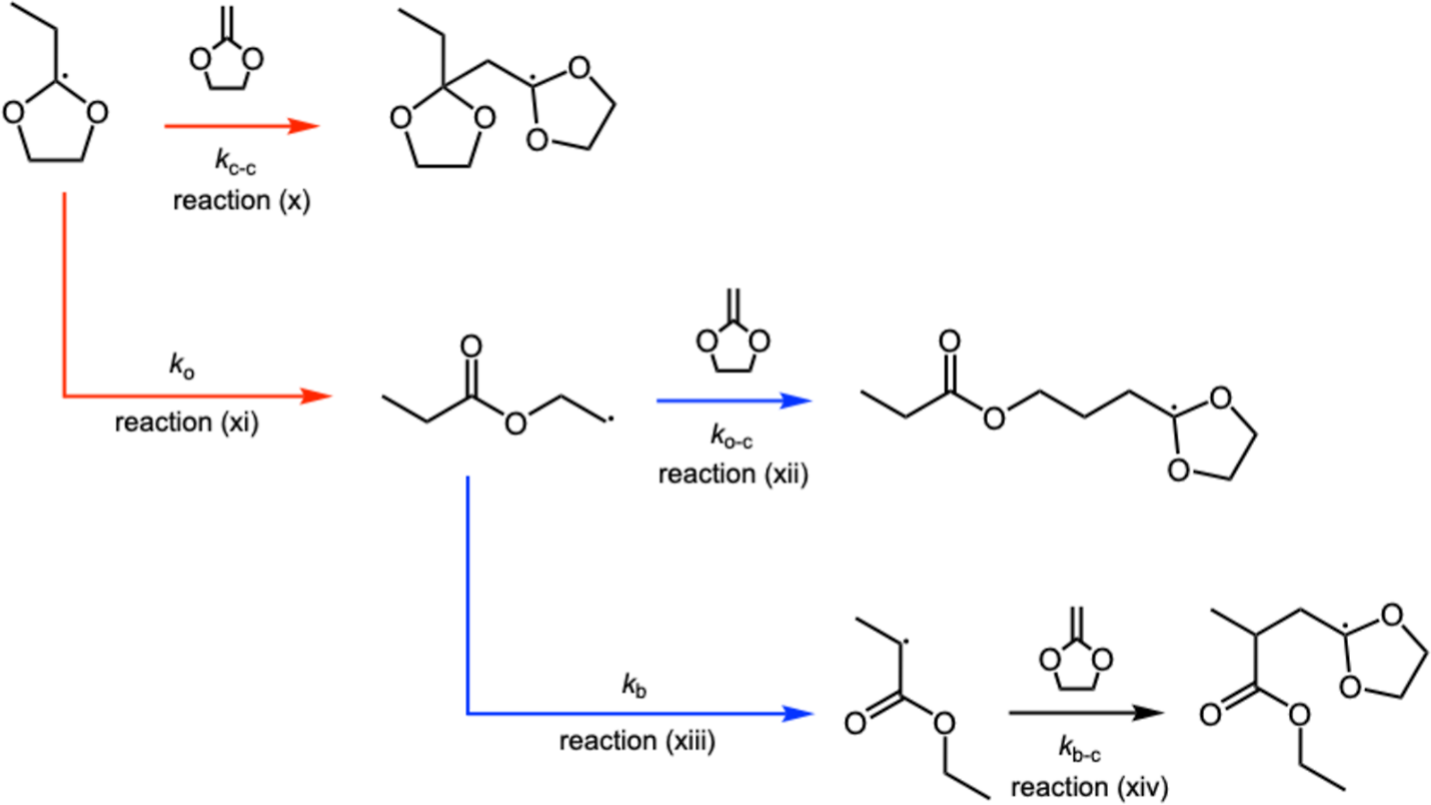
Reaction Scheme Illustrating the Possible Pathways for Propagation of **C5** Polymerization

**Table 4:** Summary of DFT Calculations for the Propagation of **C5** Using the B3LYP/6-31G* Method^a^

reactions	Δ*H* ^‡^ (kJ/mol)	Δ*S* ^‡^ (kJ/(mol K))	Δ*G* ^‡^ (kJ/mol)	*k* (L/(mol s))^b^
(x) *c*–*c*	27.6	–0.139	69.1	4.87
(xi) *o*	57.4	8.0 × 10^–6^	57.4	5.36 × 10^2^ (/s)^c^
(xii) *o*–*c*	15.5	–0.176	68.0	7.45
(xiii) *b*	79.8	–1.12 × 10^–2^	83.2	1.66 × 10^–2^ (/s)^c^
(xiv) *b*–*c*	23.8	–0.137	64.7	28.6

a The simulations were performed at 298.15 K (25 °C). Details of the reaction pathways are shown in Scheme 5.

b Calculated from Eyring equation for bimolecular reaction: *k* = (*k*_B_·*T*/*h*)·(1/*c*_0_) exp­(−Δ*G* ^‡^/*RT*).

c Calculated from Eyring equation for monomolecular reaction: *k* = (*k*_B_·*T*/*h*) exp­(−Δ*G* ^‡^/*RT*).*k*_B_: Boltzmann constant. *h*: Planck constant. *R*: gas constant. *T*: absolute temperature. *c*_0_: standard concentration.

## Discussion

### Effect of 4-Position Substituent on Composition of Copolymers Generated from 5-Membered CKA

We analyzed the unit composition of the resultant polymers by NMR spectroscopy using the reaction solutions without purification, because purification may remove intermediates and minor products important for mechanistic discussion. It should be noted that in this study, monomer conversion exceeded 99% in all polymerization systems. The introduction of a methoxymethyl group at the 4-position significantly affects monomer reactivity of 5-membered CKAs. A comparison of the structural compositions obtained from **5a** and **C5**, as shown in Figures 3 and 4, reveals how this substitution affects the balance of propagation reactions across a range of monomer concentrations and temperatures.

Ring-retaining structures (gray makers in Figures 3 and 4) are formed when propagation occurs without β-scission. For both **C5** and **5a**, the ${\mathrm{R̅}}_{c}$ values are consistently low under all conditions. In the following discussion, reaction numbers such as (i–xiv) refer to the mechanistic steps illustrated in Scheme 4 for **5a** (i–ix) and Scheme 5 for **C5** (x–xiv), respectively. In the case of **C5**, even at the most favorable conditions for the formation of ring-retaining structure (60 °C and 7.7 M), the ${\mathrm{R̅}}_{c}$ value did not exceed 0.2, and it decreases further with increasing temperature and decreasing monomer concentration. This value is considerably lower than those reported in previous studies, which have shown greater ring-retaining fractions under comparable conditions (0.5–1.0).^16,23^ These trends are in line with DFT-calculated rate constants: although direct propagation (reaction (x), Δ*G* ^‡^ = 69.1 kJ/mol) is moderately fast (*k* = 4.87 L mol^–1^ s^–1^), it competes with β-scission (reaction (xi), Δ*G* ^‡^ = 57.4 kJ/mol, *k* = 5.36 × 10^2^ s^–1^), which proceeds more rapidly due to a lower barrier in the unimolecular context. Thus, even the unsubstituted **C5** monomer does not strongly favor ring retention once a radical is formed. This indicates that, contrary to expectations based on **C5**’s symmetric and unsubstituted structure, β-scission is a highly favored pathway even at high monomer concentrations and low temperature. The differences between our results and those reported in previous studies^16,23^ could be attributed to slight differences in polymerization conditions and the occurrence of cationic polymerization initiated by contact with glass surfaces; the cationic polymerization of CKAs is known to form ring-retaining structures.^36^ The situation is even more pronounced for **5a**, whose ring-retaining fractions remain below 10% across all conditions. DFT calculations support this observation: the β-scission pathways (reactions (ii) and (iii)) have Δ*G* ^‡^ of 56.9 and 60.4 kJ/mol, respectively, with corresponding *k* values of 6.49 × 10^2^ and 1.62 × 10^2^ s^–1^. Although reaction (iii) proceeds more slowly than the β-scission of **C5**, reaction (ii)—β-scission at the 3*O*/4C position—is faster than reaction (xi). This suggests that the methoxymethyl substitution at the 4-position selectively lowers the activation barrier for β-scission at this site, thereby promoting ring-opening and suppressing ring-retaining propagation (reaction (i), Δ*G* ^‡^ = 69.6 kJ/mol, k = 3.94 L mol^–1^ s^–1^).

Ester structures (green markers in Figures 3 and 4), which arise from the addition of a ring-opened radical to monomer, show interesting differences between **C5** and **5a**. For **C5**, as monomer concentration decreases, the ester fraction decreases sharply, meaning that unimolecular backbiting reaction becomes kinetically dominant. In addition, the ester fraction drastically decreases with an increasing temperature. The calculated *k* value for backbiting in **C5** (reaction (xiii), Δ*G* ^‡^ = 83.2 kJ/mol, *k* = 1.66 × 10^–2^ s^–1^) is significantly smaller than that for intermolecular propagation (reaction (xii), Δ*G* ^‡^ = 68.0 kJ/mol, *k* = 7.45 L mol^–1^ s^–1^) at standard state (25 °C). However, the rate constants increase more drastically for reactions with high Δ*G* ^‡^. At 140 °C, the rate constant for the backbiting reaction increases by more than 10,000-fold compared to that at standard state, whereas that for intermolecular propagation increases by only about 8-fold. The general formula describing the relationship between temperature and rate constant, based on the Eyring equation, along with its derivation, is provided in the Supporting Information (eqs S1–S5). These DFT results support temperature dependence on the ratio of backbiting structure in the resultant polymer. For **5a**, on the other hand, ester structures are formed in high proportion across all conditions, with values often exceeding 70–90% ( ${\mathrm{R̅}}_{o1}$ + ${\mathrm{R̅}}_{o2}$ ). The proportion of backbiting structures remains consistently low across all temperatures and concentrations, typically not exceeding 30%. This limited formation of backbiting units can be rationalized by the large kinetic disparity between intermolecular propagation and backbiting reactions. According to the DFT results at standard state, the rate constant for intermolecular propagation via the 3*O*/4C radical (reaction (iv), Δ*G* ^‡^ = 62.9 kJ/mol, *k* = 58.3 L mol^–1^ s^–1^) is substantially greater than that of the competing backbiting pathway (reaction (vi), Δ*G* ^‡^ = 87.8 kJ/mol, *k* = 2.60 × 10^–3^ s^–1^). Although these rate constants differ in reaction order, assuming a monomer concentration of 1 M (relatively dilute condition for polymerization), the initial rate of propagation is faster than that of backbiting by more than 20,000-fold at 25 °C. Both rate constants increase with temperature, but the large disparity at 25 °C ensures that propagation remains kinetically dominant. As a result, even at elevated temperatures such as 100 and 140 °C, the rate of the backbiting reaction does not surpass that of propagation. This explains the minimal increase in backbiting fractions observed experimentally for **5a** under high temperature conditions. This behavior stands in contrast to that of **C5**, where the initial difference in rate constants is smaller. In the case of **C5**, temperature has a more pronounced effect on shifting the balance toward backbiting, particularly at low monomer concentrations. Therefore, the resistance of **5a** to form backbiting structures, even under thermally activated conditions, stems from the inherently large kinetic preference for propagation at standard state. Furthermore, the predominance of 3*O*/4C-derived esters (solid green circles in Figure 3) over 1*O*/5C-derived esters (open green circles in Figure 3) reflects a strong regioselectivity in the β-scission process induced by the asymmetric substitution at the 4-position.

### Polymerization Model for the Asymmetric and Symmetric CKAs

In the polymerization of CKAs, multiple reactions compete in a complex manner during the propagation process. This complexity is particularly pronounced in asymmetric CKAs compared to their symmetric counterparts. Therefore, a simple comparison of individual rate constants is insufficient to accurately predict polymerization behavior. Tardy et al. reported that the relationship between the rate constants of β-scission and monomer addition of the acetalyl radical (i.e., the *c* radical) is critical for predicting the composition of ester structures in polymers derived from CKAs with various ring sizes.^16^ Their study is particularly useful, as the proposed framework is applicable to both non-substituted and asymmetric CKAs such as 4-position-modified CKAs. However, backbiting reactions are not considered in their theory. In addition, asymmetric CKAs undergo two distinct types of β-scissions, leading to the formation of different ester units in the resulting polymers. In this study, we experimentally analyzed the composition of the resulting polymers’ structure, taking into account backbiting reactions and two distinct types of β-scissions. Based on these results, the relationship between polymer composition and the individual rate constants was investigated.

Herein, we assumed that (1) the steady-state approximation and terminal model are applicable to the polymerization of CKAs; (2) monomer consumption during the initiation process is negligible; (3) the composition of the resulting polymers is determined during the propagation process; (4) the concentration ratios of each propagating radical are determined solely by the rate constants for their formation; and (5) termination and intermolecular chain transfer reactions are negligible. Five kinds of radicals and one monomer exist in the polymerization of the asymmetric CKAs. Let the initial monomer concentration be [M]_0_ = β, and the final concentration after polymerization be [M]_f_ = α = (1 – conversion)­[M]_0_. The ratio of ring-retaining structure (*R*_c_) and the ratio of ring-opening structure (*R*_op_) in the resulting polymer are given by

3 $$R_{c}=\frac{k_{c-c}\left[M\right]}{k_{o1}+k_{o2}+k_{c-c}\left[M\right]}$$

4 $$R_{op}=\frac{k_{o1}+k_{o2}}{k_{o1}+k_{o2}+k_{c-c}\left[M\right]}$$

5 $$R_{op}=1-R_{c}$$

Let us define

6 $$X=k_{o1}+k_{o2}$$

Then eq 3 becomes

7 $$R_{c}=\frac{k_{c-c}\left[M\right]}{X+k_{c-c}\left[M\right]}$$

In an actual polymerization system, the monomer concentration changes continuously over time. Therefore, the resulting polymer has an average composition that reflects the structures formed at monomer concentrations between the initial and final values. The average ring-retaining ratio in the polymers ${\mathrm{R̅}}_{c}$ , obtained over the conversion form β to α, is

8 $${\mathrm{R̅}}_{c}=\frac{1}{\beta -\alpha }\int_{\alpha }^{\beta }R_{c}d\left[M\right]=1-\frac{X}{k_{c-c}\left(\beta -\alpha \right)}\ln\left(\frac{\beta k_{c-c}+X}{\alpha k_{c-c}+X}\right)$$

Similarly, the average ring-opening ratio in the polymers ${\mathrm{R̅}}_{op}$ is

9 $${\mathrm{R̅}}_{op}=\frac{1}{\beta -\alpha }\int_{\alpha }^{\beta }R_{op}d\left[M\right]=\frac{X}{k_{c-c}\left(\beta -\alpha \right)}\ln\left(\frac{\beta k_{c-c}+X}{\alpha k_{c-c}+X}\right)$$

The ring-opening structures consist of two types of β-scission events

10 $$R_{op}=R_{op1}+R_{op2}$$

where

11 $$R_{op1}=\frac{k_{o1}}{X+k_{c-c}\left[M\right]}$$

12 $$R_{op2}=\frac{k_{o2}}{X+k_{c-c}\left[M\right]}$$

Their average values considering conversion are

13 $${\mathrm{R̅}}_{op1}=\frac{k_{o1}}{k_{c-c}\left(\beta -\alpha \right)}\ln\left(\frac{\beta k_{c-c}+X}{\alpha k_{c-c}+X}\right)$$

14 $${\mathrm{R̅}}_{op2}=\frac{k_{o2}}{k_{c-c}\left(\beta -\alpha \right)}\ln\left(\frac{\beta k_{c-c}+X}{\alpha k_{c-c}+X}\right)$$

Each β-scission-derived ring-opening structure is further subdivided into ester-type structures (*R*_0_) and backbiting structures (*R*_b_)­

15 $$R_{op1}=R_{o1}+R_{b1}$$

16 $$R_{op2}=R_{o2}+R_{b2}$$

17 $$R_{op}=R_{o1}+R_{b1}+R_{o2}+R_{b2}$$

The ratio of ester structures is determined by the competition between radical–monomer addition and intramolecular backbiting reaction

18 $$R_{o1}=R_{op1}\times \frac{k_{o1-c}\left[M\right]}{k_{o1-c}\left[M\right]+k_{b1}}$$

19 $$R_{o2}=R_{op2}\times \frac{k_{o2-c}\left[M\right]}{k_{o2-c}\left[M\right]+k_{b2}}$$

Expressed in quadratic form

20 $$R_{o1}=\frac{D\left[M\right]}{A{\left[M\right]}^{2}+B\left[M\right]+C}$$

21 $$R_{o2}=\frac{D^{\prime }\left[M\right]}{{A^{\prime }\left[M\right]}^{2}+B^{\prime }\left[M\right]+C^{\prime }}$$

where

$$A=k_{c-c}k_{o1-c},B={k_{c-c}k_{b1}+k}_{o1-c}\left(k_{o1}+k_{o2}\right),C=k_{b1}\left(k_{o1}+k_{o2}\right),D=k_{o1}k_{o1-c}$$

$$A^{\prime }=k_{c-c}k_{o2-c},B^{\prime }={k_{c-c}k_{b2}+k}_{o2-c}\left(k_{o1}+k_{o2}\right),C^{\prime }=k_{b2}\left(k_{o1}+k_{o2}\right),D^{\prime }=k_{o2}k_{o2-c}$$

The average of ester ratios

22 $$\begin{array}{l} {\mathrm{R̅}}_{o1}=\frac{1}{\beta -\alpha }\int_{\alpha }^{\beta }\frac{D\left[M\right]}{A{\left[M\right]}^{2}+B\left[M\right]+C}d\left[M\right]=\\ \frac{D}{2A\left(\beta -\alpha \right)}\ln\left(\frac{Q\left(\beta \right)}{Q\left(\alpha \right)}\right)-\frac{BD}{2A\left(\beta -\alpha \right)\sqrt{\Delta }}\ln\left(|\frac{L\left(\beta \right)-\sqrt{\Delta }}{L\left(\beta \right)+\sqrt{\Delta }}\cdot \frac{L\left(\alpha \right)+\sqrt{\Delta }}{L\left(\alpha \right)-\sqrt{\Delta }}|\right) \end{array}$$

$$\begin{array}{l} {\mathrm{R̅}}_{o2}=\frac{1}{\beta -\alpha }\int_{\alpha }^{\beta }\frac{D^{\prime }\left[M\right]}{A^{\prime }{\left[M\right]}^{2}+B^{\prime }\left[M\right]+C^{\prime }}d\left[M\right]=\\ \frac{D^{\prime }}{2A^{\prime }\left(\beta -\alpha \right)}\ln\left(\frac{Q^{\prime }\left(\beta \right)}{Q^{\prime }\left(\alpha \right)}\right)-\frac{B^{\prime }D^{\prime }}{2A^{\prime }\left(\beta -\alpha \right)\sqrt{\Delta^{\prime }}}\ln\left(|\frac{L^{\prime }\left(\beta \right)-\sqrt{\Delta^{\prime }}}{L^{\prime }\left(\beta \right)+{\sqrt{\Delta }}^{\prime }}\cdot \frac{L^{\prime }\left(\alpha \right)+\sqrt{\Delta^{\prime }}}{L^{\prime }\left(\alpha \right)-\sqrt{\Delta^{\prime }}}|\right) \end{array}$$ 23

where

$$\Delta =B^{2}-4AC,Q\left(x\right)=Ax^{2}+Bx+C,L\left(x\right)=2Ax+B$$

$$\Delta^{\prime }={B^{\prime }}^{2}-4A^{\prime }C^{\prime },Q^{\prime }\left(x\right)=A^{\prime }x^{2}+B^{\prime }x+C^{\prime },L^{\prime }\left(x\right)=2A^{\prime }x+B^{\prime }$$

The backbiting structures are formally formed when *b*_1_ and *b*_2_ radicals react with CKA. However, in practice, the generation of *b*_1_ and *b*_2_ radicals results in the incorporation of backbiting structures into the polymer. Therefore, the following expressions hold

24 $$R_{b1}=R_{op1}\times \frac{k_{b1}}{k_{o1-c}\left[M\right]+k_{b1}}=\frac{E}{A{\left[M\right]}^{2}+B\left[M\right]+C}$$

$$R_{b2}=R_{op2}\times \frac{k_{b2}}{k_{o2-c}\left[M\right]+k_{b2}}=\frac{E^{\prime }}{{A^{\prime }[M]}^{2}+B^{\prime }\left[M\right]+C^{\prime }}$$ 25

where

$$E=k_{o1}k_{b1},E^{\prime }=k_{o2}k_{b2}$$

Their average values are shown below

26 $$\begin{array}{l} {\mathrm{R̅}}_{b1}=\frac{1}{\beta -\alpha }\int_{\alpha }^{\beta }\frac{E}{A{\left[M\right]}^{2}+B\left[M\right]+C}d\left[M\right]=\\ \frac{E}{\left(\beta -\alpha \right)\sqrt{\Delta }}\ln\left(\frac{L\left(\beta \right)-\sqrt{\Delta }}{L\left(\beta \right)+\sqrt{\Delta }}\cdot \frac{L\left(\alpha \right)+\sqrt{\Delta }}{L\left(\alpha \right)-\sqrt{\Delta }}\right) \end{array}$$

$$\begin{array}{l} {\mathrm{R̅}}_{b2}=\frac{1}{\beta -\alpha }\int_{\alpha }^{\beta }\frac{E^{\prime }}{A^{\prime }{\left[M\right]}^{2}+B^{\prime }\left[M\right]+C^{\prime }}d\left[M\right]=\\ \frac{E^{\prime }}{\left(\beta -\alpha \right)\sqrt{\Delta^{\prime }}}\ln\left(\frac{L^{\prime }\left(\beta \right)-{\sqrt{\Delta }}^{\prime }}{L^{\prime }\left(\beta \right)+\sqrt{\Delta^{\prime }}}\cdot \frac{L^{\prime }\left(\alpha \right)+\sqrt{\Delta^{\prime }}}{L^{\prime }\left(\alpha \right)-\sqrt{\Delta^{\prime }}}\right) \end{array}$$ 27

In the case of symmetric CKAs, although three types of radicals and one monomer are involved, each formula can be derived in a similar manner. The derivation of these formulas is provided in the Supporting Information (eqs S6–S16).

To further evaluate the validity of the proposed kinetic model, the average structural compositions of polymers derived from the asymmetric **5a** were theoretically calculated and compared with experimental results. The final monomer concentrations (α values) were calculated from the initial monomer concentrations (β values), assuming 99% monomer conversion. Using the rate constants of **5a** polymerization at 25 °C obtained from DFT calculations and eq S5, the rate constants at 60, 100, and 140 °C were estimated according to the Eyring equation. These values were then applied to eqs 13, 14, 22, 23, 26, and 27 to compute the average ratios of each structure (ring-retaining, ester, and backbiting) as a function of initial monomer concentration. The results are shown in Figure 3, where the calculated theoretical values are drawn as solid curves. The model reproduces key experimental trends observed across all temperatures. For example, an increase in the initial monomer concentration leads to a noticeable rise in the average ring-retaining fraction ${\mathrm{R̅}}_{c}$ , which can be rationalized by the fact that the bimolecular propagation of the *c* radical (reaction rate *V*_*c*–*c*_ = *k*_*c*–*c*_[*c*·]­[M]) becomes increasingly dominant at higher monomer concentrations. In contrast, the β-scission process (a first-order reaction) remains largely unaffected by concentration, thus becoming relatively less competitive under these conditions. This explains the rising trend in ${\mathrm{R̅}}_{c}$ with increasing [M]_0_ and highlights the model’s ability to capture the concentration dependence of ring-retaining pathways. Although the model captures the overall trends, some discrepancies are observed. In particular, the predicted ${\mathrm{R̅}}_{c}$ values tend to slightly underestimate the experimentally observed ring-retaining fractions at the highest concentrations, especially at lower temperatures. This deviation may result from factors not fully accounted for in the model, such as nonideal reactions including termination and chain transfer reactions. Additionally, the model assumes rate constants remain constant throughout the reaction, whereas in reality, local environment changes (e.g., viscosity, polarity) may influence the effective reactivity of intermediates. Nevertheless, the theoretical model provides a quantitative basis for understanding how temperature and monomer concentration influence polymer composition.

A similar analysis was performed for the unsubstituted symmetric **C5** using the corresponding rate constants obtained from DFT calculations. The calculated average compositions for the **C5** polymer also reproduced the general experimental trends shown in Figure 4. For example, the fraction of backbiting structures sharply increased at low monomer concentrations and high temperatures. However, compared to **5a**, the model predictions for **C5** show slightly greater deviations from the experimental data, especially at the lower temperature. One possible reason is that the kinetic competition between β-scission and propagation in **C5** is more sensitive to subtle variations in reaction conditions, as the differences in the corresponding rate constants are smaller than those in **5a**. For instance, at 25 °C, the ratio between intermolecular propagation (reaction (xii), *k* = 7.45 L mol^–1^ s^–1^) and backbiting (reaction (xiii), *k* = 1.66 × 10^–2^ s^–1^) in **C5** is approximately 450, while in **5a**, the analogous ratio between reactions (iv) and (vi) exceeds 20,000. Consequently, small changes in temperature or concentration in **C5** can more easily shift the dominant pathway, making the model more sensitive to kinetic approximations.

The theoretical model does not explicitly account for possible differences in the natures of the propagating radicals, such as solvation or steric effects, which may differ between **C5** and **5a** due to the presence of the methoxymethyl substituent. These effects could influence the effective reaction barriers in ways not fully captured by gas-phase DFT calculations. Despite these minor discrepancies, the theoretical curves for both **C5** and **5a** provide a good semiquantitative description of the experimentally observed trends. In both cases, the model successfully predicts the dominant structural motifs as a function of monomer concentration and temperature, highlighting the critical role of substitution at the 4-position in controlling the competition between propagation, β-scission, and backbiting pathways. Additionally, **5b–d** were polymerized at 100 and 140 °C with a monomer concentration of 2.4 M. The chemical structures were determined by ^1^H NMR spectroscopy of the purified polymers, with spectral assignments made by reference to the NMR data of the **5a** polymer. The average composition ratios, ${\mathrm{R̅}}_{c}$ , ${\mathrm{R̅}}_{op1}$ , ${\mathrm{R̅}}_{op2}$ , ${\mathrm{R̅}}_{o1}$ , ${\mathrm{R̅}}_{o1}$ , ${\mathrm{R̅}}_{b1}$ , and ${\mathrm{R̅}}_{b2}$ , were determined by ^1^H NMR analysis of the resultant polymers without purification. The results are summarized in Table S3. Assuming that the rate constants governing the polymerization of **5b–d** are the same as those for **5a**, the theoretical model provides a good match with the experimental composition ratios obtained under the same conditions. This consistency supports the applicability of the proposed kinetic framework to a broader range of 4-position-substituted CKAs.

In the present kinetic model, termination and chain transfer processes, including intermolecular hydrogen transfer and transfer to monomer, were not considered in order to maintain an analytically solvable framework. These reactions may occur under certain conditions—particularly at high monomer concentrations—but were intentionally omitted to focus on the dominant elementary steps governing polymer composition. A key advantage of this simplified model is that all required rate constants can be reasonably estimated by DFT calculations, as propagation and β-scission pathways involve well-defined transition states. In contrast, evaluating rate constants for chain transfer reactions—especially to monomer or polymer—would require exhaustive identification of all possible hydrogen abstraction sites, leading to a prohibitively large number of calculations. Despite this simplification, the model quantitatively reproduces the experimentally observed trends across a range of monomer concentrations and temperatures, demonstrating its robustness and relevance. This framework thus offers a practical and predictive tool for analyzing structure–reactivity relationships in CKA polymerization, while allowing future incorporation of additional side reactions when detailed kinetic data become available.

## Conclusion

In this study, we have elucidated the radical polymerization behavior of asymmetric 5-membered CKAs bearing alkoxymethyl groups at the 4-position, focusing on the relationship between monomer and structures. Detailed NMR analyses revealed the structural complexity of the resulting polymers, which was successfully rationalized using a kinetic model incorporating DFT-calculated rate constants for propagation, β-scission, and backbiting reactions. The model demonstrated excellent predictive power across varying monomer concentrations and temperatures.

Importantly, the integration of theoretical and experimental insights allowed us to identify critical parameters governing the formation of degradable ester units, which was corroborated by OECD 301F biodegradation tests. These findings underscore the potential of monomer structure engineering to tailor both the composition and degradability of radical-derived polymers. By providing a predictive framework for structural design, this work contributes to the broader effort to develop sustainable materials through mechanistically informed polymer chemistry.

## Supplementary Material



## Notes

Detailed ^1^H and ^13^C NMR assignments, derivation of the kinetic model for symmetric CKAs, 2D NMR spectra, Biodegradability data, 3D structure and Cartesian coordinate of the transition states (PDF)
