# Supplementary material for: Kinetic Model of Radical Ring-Opening Polymerization of Asymmetric Five-Membered Cyclic Ketene Acetals
Source: Macromolecules. 2025 Aug 19;58(17):9387–401. doi: 10.1021/acs.macromol.5c01438 (PMC12424300; doi:10.1021/acs.macromol.5c01438)
Supplement: Supplementary file 1 [file ma5c01438_si_001.pdf]

Kinetic Model of Radical Ring-Opening Polymerization of Asymmetric Five-Membered Cyclic Ketene Acetals

Shin-nosuke Nishimura,[1] Marina Uryu,[1] and Tomoyuki Koga[1*]*

[1]Department of Molecular Chemistry and Biochemistry, Faculty of Science and Engineering, Doshisha University,1-3 Tatara Miyakodani, Kyotanabe, Kyoto 610-0321, JAPAN, Tel:+81-774-65-6622; +81-774-65-6621

e-mail: shnishim@mail.doshisha.ac.jp, tkoga@mail.doshisha.ac.jp

KEYWORDS: Cyclic Ketene Acetal, Radical Ring-Opening Polymerization, Kinetic Model, Asymmetric Structure

Contents

Structural assignment of the polymer generated by polymerization of **5a** 3
General formula describing the relationship between temperature and rate constant based on the Eyring equation 5
Derivation of the model formula for symmetrical CKAs 6
Figure S1. ^{1}H and ^{13}C NMR spectra of **1a** 9
Figure S2. ^{1}H and ^{13}C NMR spectra of **1b** 10
Figure S3. ^{1}H and ^{13}C NMR spectra of **1c** 11
Figure S4. ^{1}H and ^{13}C NMR spectra of **2a** 12
Figure S5. ^{1}H and ^{13}C NMR spectra of **2b** 13
Figure S6. ^{1}H and ^{13}C NMR spectra of **2c** 14
Figure S7. ^{1}H and ^{13}C NMR spectra of **2d** 15
Figure S8. ^{1}H and ^{13}C NMR spectra of **3a** 16
Figure S9. ^{1}H and ^{13}C NMR spectra of **3b** 17
Figure S10. ^{1}H and ^{13}C NMR spectra of **3c** 18
Figure S11. ^{1}H and ^{13}C NMR spectra of **3d** 19
Figure S12. ^{1}H and ^{13}C NMR spectra of **4a** 20
Figure S13. ^{1}H and ^{13}C NMR spectra of **4b** 21
Figure S14. ^{1}H and ^{13}C NMR spectra of **4c** 22
Figure S15. ^{1}H and ^{13}C NMR spectra of **4d** 23
Figure S16. ^{1}H and ^{13}C NMR spectra of **5b** 24
Figure S17. ^{1}H and ^{13}C NMR spectra of **5c** 25
Figure S18. ^{1}H and ^{13}C NMR spectra of **5d** 26
Figure S19. Diad structures of the polymer generated by polymerization of **5a** 27
Figure S20. DEPT analyses of the polymer generated by polymerization of **5a** 28
Figure S21. ^{1}H-^{1}H COSY and ^{1}H-^{1}H TOCSY spectra of the polymer generated by polymerization of **5a** 29
Figure S22. ^{1}H-^{13}C HMQC spectrum of the polymer generated by polymerization of **5a** 30
Figure S23. ^{1}H-^{13}C HMBC spectrum of the polymer generated by polymerization of **5a** 31
Figure S24. Biodegradability test of the **5a** polymer 32
Table S1. Summary of assignment of carbon signals for the **5a** polymer 33
Table S2. Summary of assignment of proton signals for the **5a** polymer 34
Table S3. Summary of polymerization of **5b-5d** 35
The 3D structures and cartesian coordinates for the transition states 36

Structural assignment of the polymer generated by polymerization of 5a

Initially, the carbon atoms in the polymer were classified as primary (1°), secondary (2°), tertiary (3°), quaternary (4°), or carbonyl carbons based on ^{13}C NMR and DEPT spectra (Figure S20, Table S1). Carbons not directly bonded to protons were assigned to quaternary carbons in the c structure and to the ester carbonyl groups in the ring-opened structures (*o*1, *o*2, *b*1, and *b*2). These signals disappeared in DEPT spectra under all flip angles. The 4° carbon in the *c* structure was observed at 109.6 ppm, while the carbonyl carbons appeared at 169.9, 171.7, 172.0, and 172.6 ppm. The tertiary carbons were assigned to 35.2, 69.4, 71.7, and 74.3 ppm. Primary and secondary carbon assignments are summarized in Table S1. Based on these carbon assignments, corresponding proton environments were identified using ^{1}H-^{13}C HMQC analysis (Figure S22), with full details in Table S2. In the ^{1}H-^{1}H COSY spectrum, the absence of correlations between tertiary protons suggested low abundance of *o*1-*b*1, *o*1-*b*2, *b*1-*b*1, *b*1-*b*2, *b*2-*b*1, and *b*2-*b*2 diads. Furthermore, the ^{1}H-^{1}H TOCSY spectrum lacked correlations between multiple secondary and tertiary protons, indicating limited formation of *o*2-*b*1 and *o*2-*b*2 diads.

A pair of proton signals at 3.63 ppm (2°) and 4.29 ppm (3°) showed COSY correlation and jointly exhibited HMBC correlation with the 4° carbon at 109.6 ppm. These were assigned to protons at the 5- and 4-positions, respectively, of the cyclic acetal ring in the *c* structure. The 3° proton at 4.29 ppm further correlated with a 2° proton at 3.35 ppm adjacent to an ether oxygen. The corresponding 3° carbon at 74.3 ppm correlated with a methoxy proton at 3.39 ppm. The 2° protons at 2.23 and 2.63 ppm correlated only with the 4° carbon at 109.6 ppm and were therefore assigned to CH_2 groups in the main chain of *c*-*c*, *c*-*o*1, and *c*-*o*2 diads, which appear to be minor components. Although a long-range $^{3}J_{CH}$ coupling with ester carbonyl carbon is theoretically

possible in the *o*2 structure, no such correlation was observed, likely due to the small magnitude of the coupling constant. This prevented differentiation between *c*-*o*1 and *c*-*o*2 diads.

In another region, the 3° proton at 2.45 ppm displayed COSY correlations with three distinct 2° protons at 2.40, 3.35, and 4.11 ppm. The associated 3° carbon at 35.2 ppm showed HMBC correlations with all three, consistent with the *o*1 structure. These signals were assigned to CH_2 groups adjacent to the ester carbonyl (2.40 ppm), ether oxygen (3.35 ppm), and ester oxygen (4.11 ppm). The 2° carbon at 72.1 ppm (bonded to the 3.35 ppm proton) correlated with the methoxy proton at 3.39 ppm, while the carbonyl carbon at 171.7 ppm showed a cross-peak with the proton at 4.11 ppm. This pattern is consistent with both *o*1-*o*1 and *o*1-*o*2 diads, which could not be resolved.

The 3° proton signal at 5.05 ppm correlated with the 2° protons at 1.89 and 3.45 ppm in the COSY spectrum, matching the expected pattern of the *o*2 structure. The 1.89 ppm signal corresponds to a central CH_2, while the 3.45 ppm proton is adjacent to an ether oxygen. The 2° carbon at 69.1 ppm (bonded to 3.45 ppm) correlated with a methoxy proton at 3.39 ppm in HMBC. In the TOCSY spectrum, the 2.40 ppm proton adjacent to an ester carbonyl also showed correlations with the 1.89, 3.45, and 5.05 ppm signals, supporting the presence of both *o*2-*o*1 and *o*2-o2 diads. However, these could not be distinguished individually.

Another 2° proton at 4.17 ppm exhibited HMBC correlation with the carbonyl carbon at 172.6 ppm. It also correlated in COSY with the 2° proton at 1.89 ppm, and in TOCSY with the 2° proton at 3.45 ppm. These three were assigned to CH_2 units adjacent to the ester carbonyl (4.17 ppm), the ether oxygen (3.45 ppm), and the intermediate methylene (1.89 ppm) within the side chain. The 2° carbon at 69.1 ppm (attached to the proton at 3.45 ppm) correlated with the methoxy

group at 3.29 ppm. However, no long-range coupling was detected between the tertiary main-chain proton and the side-chain carbonyl carbon, hindering definitive identification of *b*1-based diads.

Finally, the 1° proton signal at 1.23 ppm showed COSY correlation with the 3° proton at 5.10 ppm. Given that the *b*2 structure contains a methyl group in the side chain, the signal at 1.23 ppm was attributed to this CH_3. The proton at 5.10 ppm further correlated with the 2° proton at 3.52 ppm, and these correlations were confirmed in the TOCSY spectrum. In HMBC spectrum, the 2° carbon at 70.8 ppm (attached to the proton at 3.52 ppm) correlated with a methoxy proton at 3.29 ppm. As before, the lack of $^3J_{CH}$ coupling to the ester carbonyl prevented definitive assignment, and *b*2-based diads could not be conclusively identified.

The ratio of the *c*, *o*1, *o*2, *b*1, and *b*2 structures in the polymers was determined based on the integration values of specific proton signals. The *c* and *b*2 structures were quantified using well-isolated tertiary proton signals at 3.63 ppm and 1.23 ppm, respectively. The *o*2 structure was quantified using the proton signal at 5.05 ppm, which overlaps with a proton signal derived from the *b*2 structure. Therefore, the content of the *o*2 structure was estimated from the differential integration value after subtracting the contribution of the *b*2 structure. The *o*1 structure was quantified using the proton signal at 4.11 ppm, which overlaps with signals derived from the *c* and *b*1 structures. Accordingly, the content of the *o*1 structure was estimated by correcting the integration value based on the known contributions from the *c* and *b*1 structures.

General formula describing the relationship between temperature and rate constant based on the Eyring equation

Lets us now consider a reaction at a standard concentration of one molar (c_0 =1) at two temperatures, T_1 and T_2 (where $T_1 < T_2$). From the Eyring equation (Eqs. (1) and (2)), the rate constants at these temperatures, k_1 and k_2 are:

$$k_1 = \frac{k_B T_1}{h} exp\left(\frac{\Delta S^{\neq}}{R}\right) exp\left(-\frac{\Delta H^{\neq}}{RT_1}\right) \tag{S1}$$

$$k_2 = \frac{k_B T_2}{h} exp\left(\frac{\Delta S^{\neq}}{R}\right) exp\left(-\frac{\Delta H^{\neq}}{RT_2}\right) \tag{S2}$$

Dividing Eq. (S2) by Eq. (S1):

$$\frac{k_2}{k_1} = \frac{T_2}{T_1} exp\left(\frac{\Delta H^{\neq}}{R}\left(\frac{1}{T_1} - \frac{1}{T_2}\right)\right) \tag{S3}$$

Taking the natural logarithm of both sides:

$$\ln\frac{k_2}{k_1} = \ln\frac{T_2}{T_1} + \frac{\Delta H^{\neq}}{R}\left(\frac{1}{T_1} - \frac{1}{T_2}\right) \tag{S4}$$

If the right-hand side evaluates to a numerical value y, then:

$$\ln\frac{k_2}{k_1} = y \Rightarrow \frac{k_2}{k_1} = e^y \tag{S5}$$

Thus, the rate constant increases by a factor of e^y with the rise in temperature.

Derivation of the model formula for symmetrical CKAs

Three kinds of radicals and one monomer exist in the polymerization of the symmetrical CKAs. Let the initial monomer concentration be $[M]_0 = \beta$, and the final concentration after polymerization

be $[M]_f = \alpha = (1 - \text{Conversion})[M]_0$. The ratio of ring-retaining structure (R_c) and the ratio of ring-opening structure (R_{op}) in the resulting polymer are given by:

$$R_c = \frac{k_{c-c}[M]}{k_o + k_{c-c}[M]} \qquad (S6)$$

$$R_{op} = \frac{k_o}{k_o + k_{c-c}[M]} \qquad (S7)$$

$$R_{op} = 1 - R_c \qquad (S8)$$

In an actual polymerization system, the monomer concentration changes continuously over time. Therefore, the resulting polymer has an average composition that reflects the structures formed at monomer concentrations between the initial and final values. The average ring-retaining ratio in the polymers $\bar{R}_c$, obtained over the conversion form β to α, is:

$$\bar{R}_c = \frac{1}{\beta - \alpha}\int_{\alpha}^{\beta} R_c d[M] = 1 - \frac{k_o}{k_{c-c}(\beta - \alpha)} \ln\left(\frac{\beta k_{c-c} + k_o}{\alpha k_{c-c} + k_o}\right) \qquad (S9)$$

Similarly, the average ring-opening ratio in the polymers $\bar{R}_{op}$ is:

$$\bar{R}_{op} = \frac{1}{\beta - \alpha}\int_{\alpha}^{\beta} R_{op} d[M] = \frac{k_o}{k_{c-c}(\beta - \alpha)} \ln\left(\frac{\beta k_{c-c} + k_o}{\alpha k_{c-c} + k_o}\right) \qquad (S10)$$

Each β-scission-derived ring-opening structure is further subdivided into ester-type structures (R_o) and back-biting structures (R_b):

$$R_{op} = R_o + R_b \qquad (S11)$$

The ratio of ester structures is determined by the competition between radical–monomer addition and intramolecular back-biting reaction:

$$R_o = R_{op} \times \frac{k_{o-c}[M]}{k_{o-c}[M] + k_b} \qquad (S12)$$

Expressed in quadratic form:

$$R_o = \frac{D[M]}{A[M]^2 + B[M] + C} \qquad (S12)$$

Where:

$$A = k_{c-c}k_{o-c}, B = k_{c-c}k_b + k_{o-c}k_o, C = k_bk_o, D = k_ok_{o-c}$$

The average of ester ratios:

$$\bar{R}_o = \frac{1}{\beta - \alpha}\int_{\alpha}^{\beta} \frac{D[M]}{A[M]^2 + B[M] + C} d[M] =$$

$$\frac{D}{2A(\beta - \alpha)} \ln\left(\frac{Q(\beta)}{Q(\alpha)}\right) - \frac{BD}{2A(\beta - \alpha)\sqrt{\Delta}} \ln\left(\left|\frac{L(\beta) - \sqrt{\Delta}}{L(\beta) + \sqrt{\Delta}} \cdot \frac{L(\alpha) + \sqrt{\Delta}}{L(\alpha) - \sqrt{\Delta}}\right|\right) \qquad (S13)$$

Where:

$$\Delta = B^2 - 4AC, Q(x) = Ax^2 + Bx + C, L(x) = 2Ax + B,$$

The back-biting structures are formally formed when *b* radical react with CKA. However, in practice, the generation of *b* radical results in the incorporation of back-biting structures into the polymer. Therefore, the following expressions hold:

$$R_B = R_{op} \times \frac{k_b}{k_{o-c}[M] + k_b} = \frac{E}{A[M]^2 + B[M] + C} \qquad (S14)$$

Where:

$$E = k_ok_b \qquad (S15)$$

Their average values are shown below:

$$\bar{R}_B = \frac{1}{\beta - \alpha}\int_{\alpha}^{\beta} R_B d[M] = \frac{E}{(\beta - \alpha)\sqrt{\Delta}} \ln\left(\frac{L(\beta) - \sqrt{\Delta}}{L(\beta) + \sqrt{\Delta}} \cdot \frac{L(\alpha) + \sqrt{\Delta}}{L(\alpha) - \sqrt{\Delta}}\right) \qquad (S16)$$

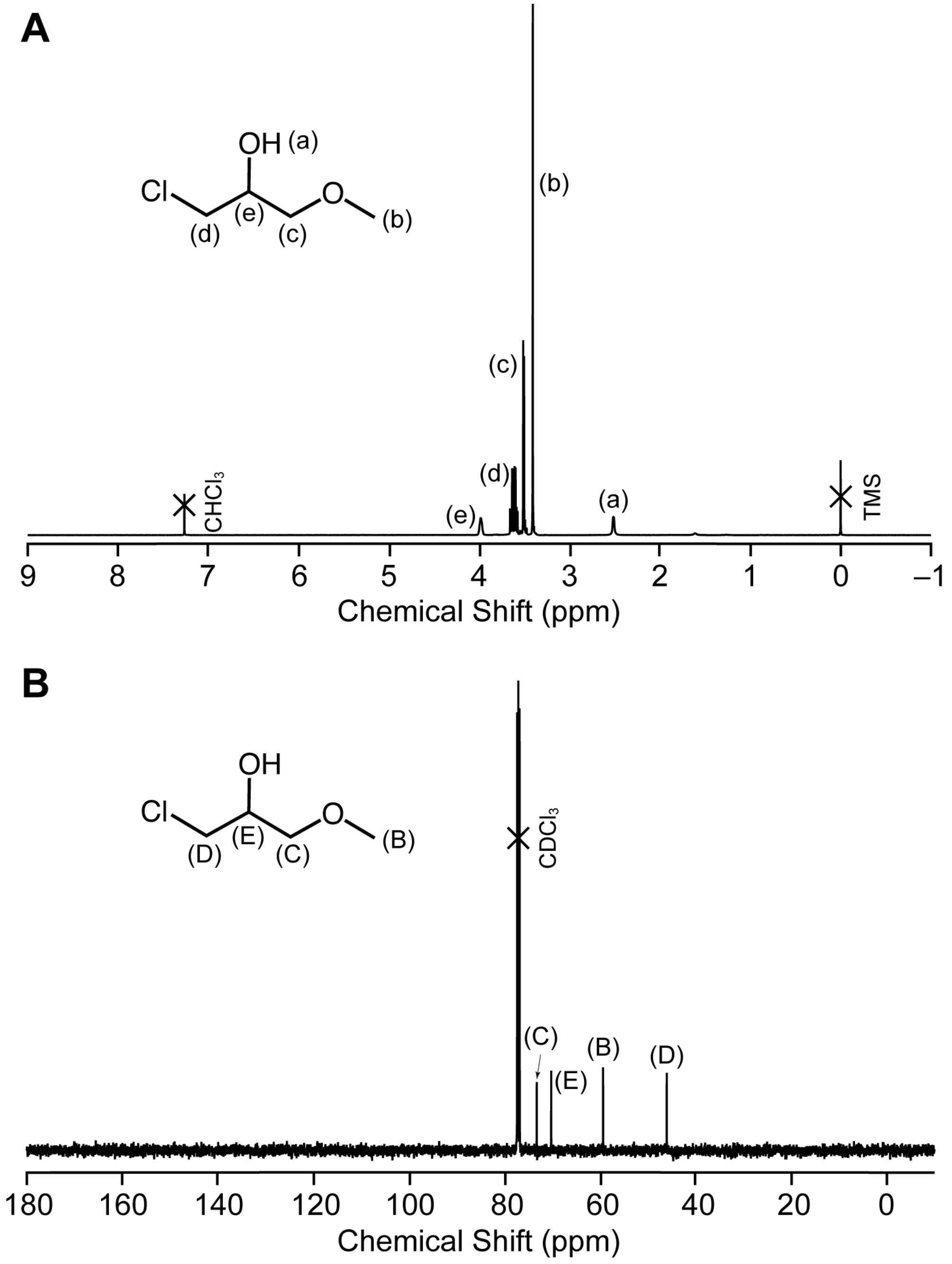

Figure S1. (A) ^{1}H and (B) ^{13}C NMR spectra of **1a** in $CDCl_3$ at 25 °C.

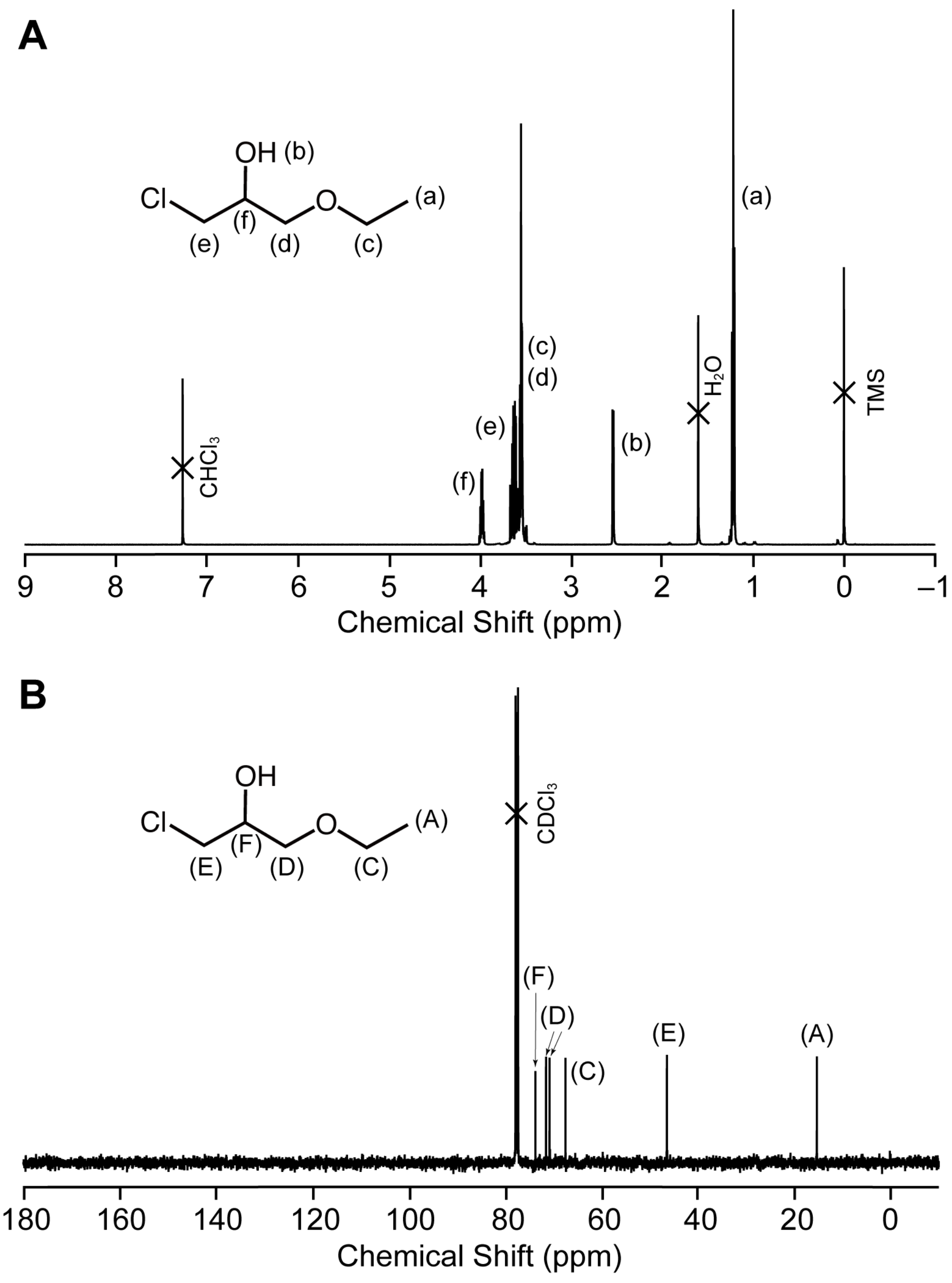

Figure S2. (A) ^{1}H and (B) ^{13}C NMR spectra of **1b** in $CDCl_3$ at 25 °C.

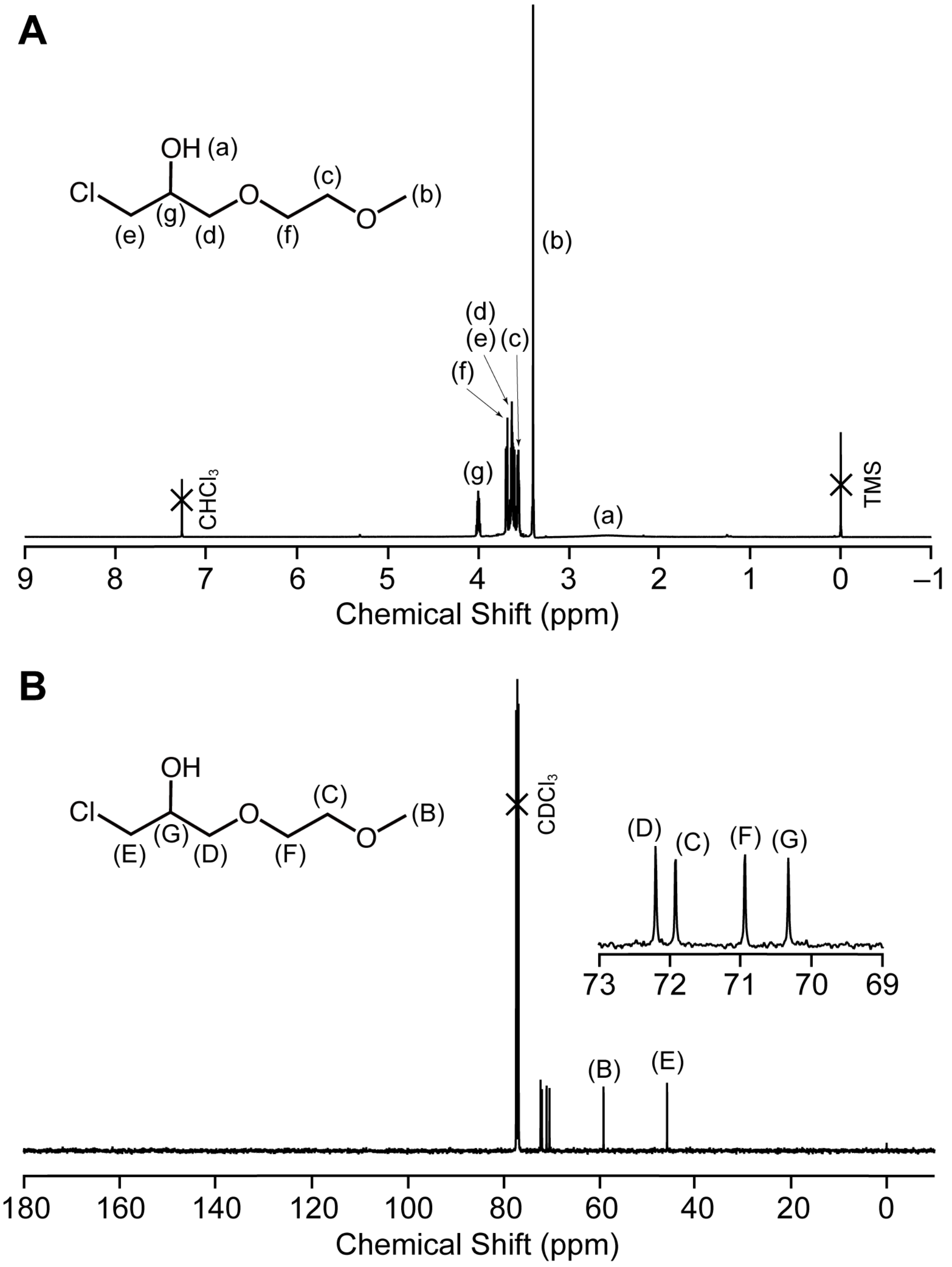

Figure S3. (A) ^{1}H and (B) ^{13}C NMR spectra of **1c** in $CDCl_3$ at 25 °C.

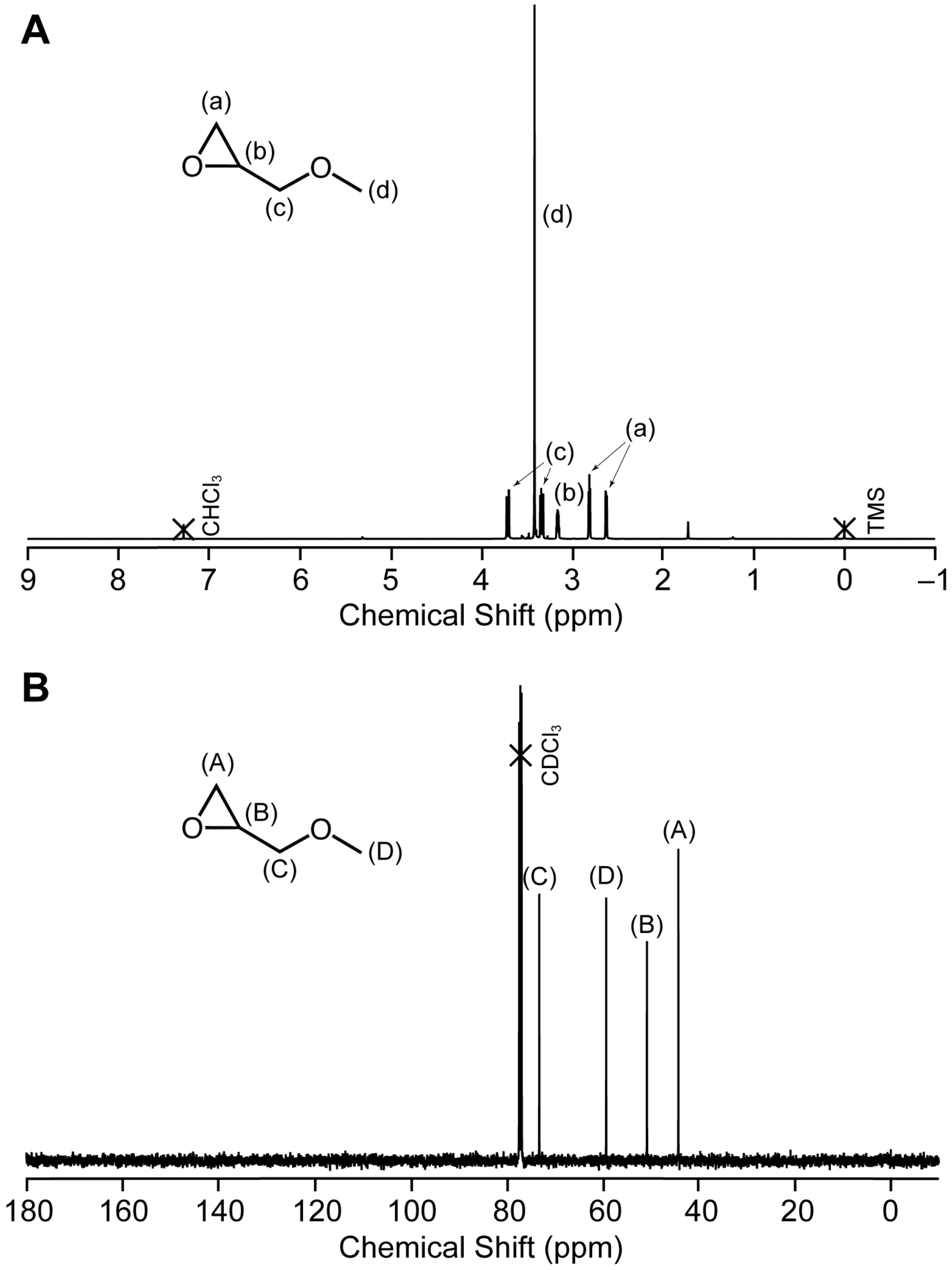

Figure S4. (A) ^{1}H and (B) ^{13}C NMR spectra of **2a** in $CDCl_3$ at 25 °C.

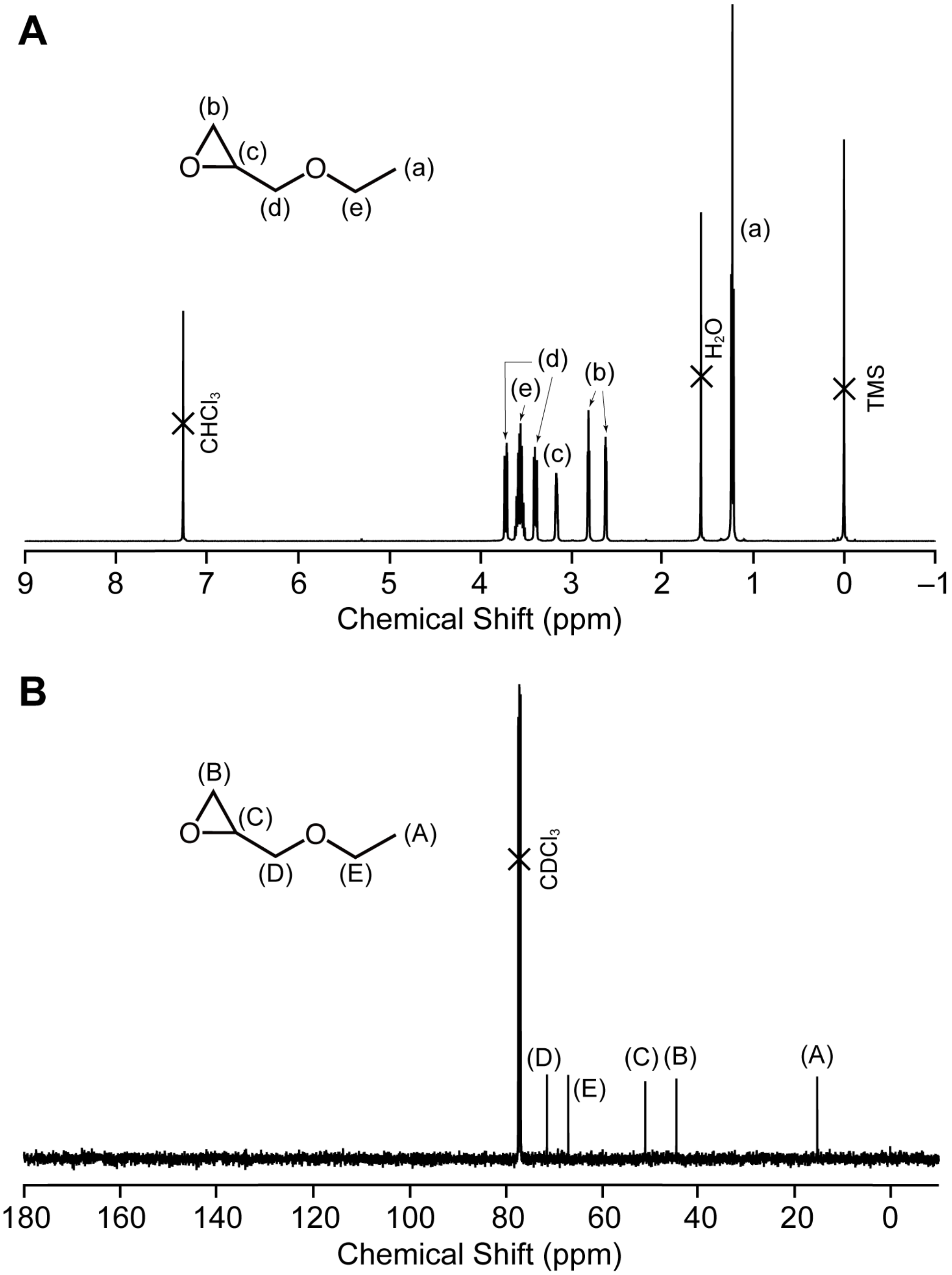

Figure S5. (A) ^{1}H and (B) ^{13}C NMR spectra of **2b** in $CDCl_3$ at 25 °C.

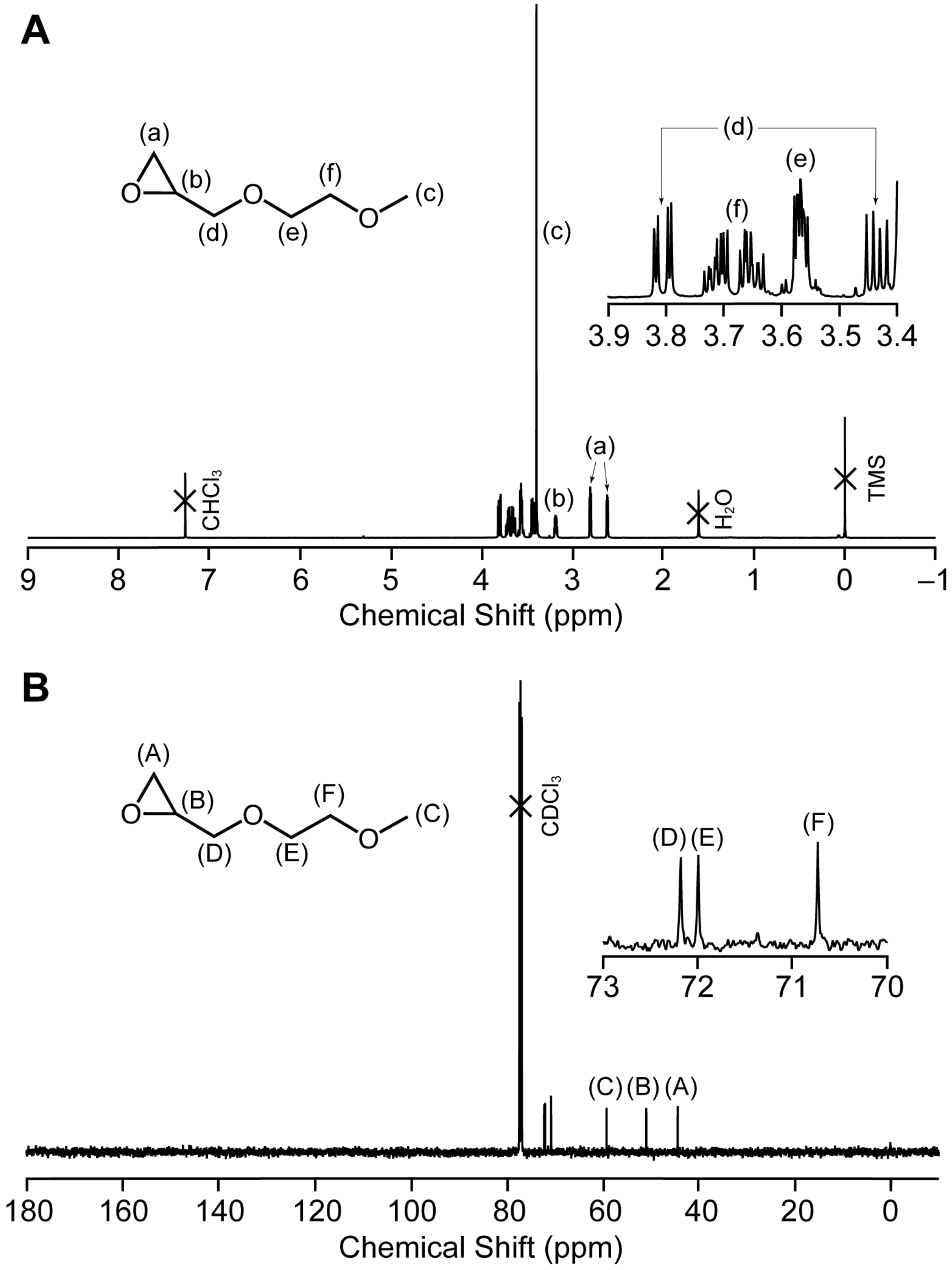

Figure S6. (A) ^{1}H and (B) ^{13}C NMR spectra of **2c** in $CDCl_3$ at 25 °C.

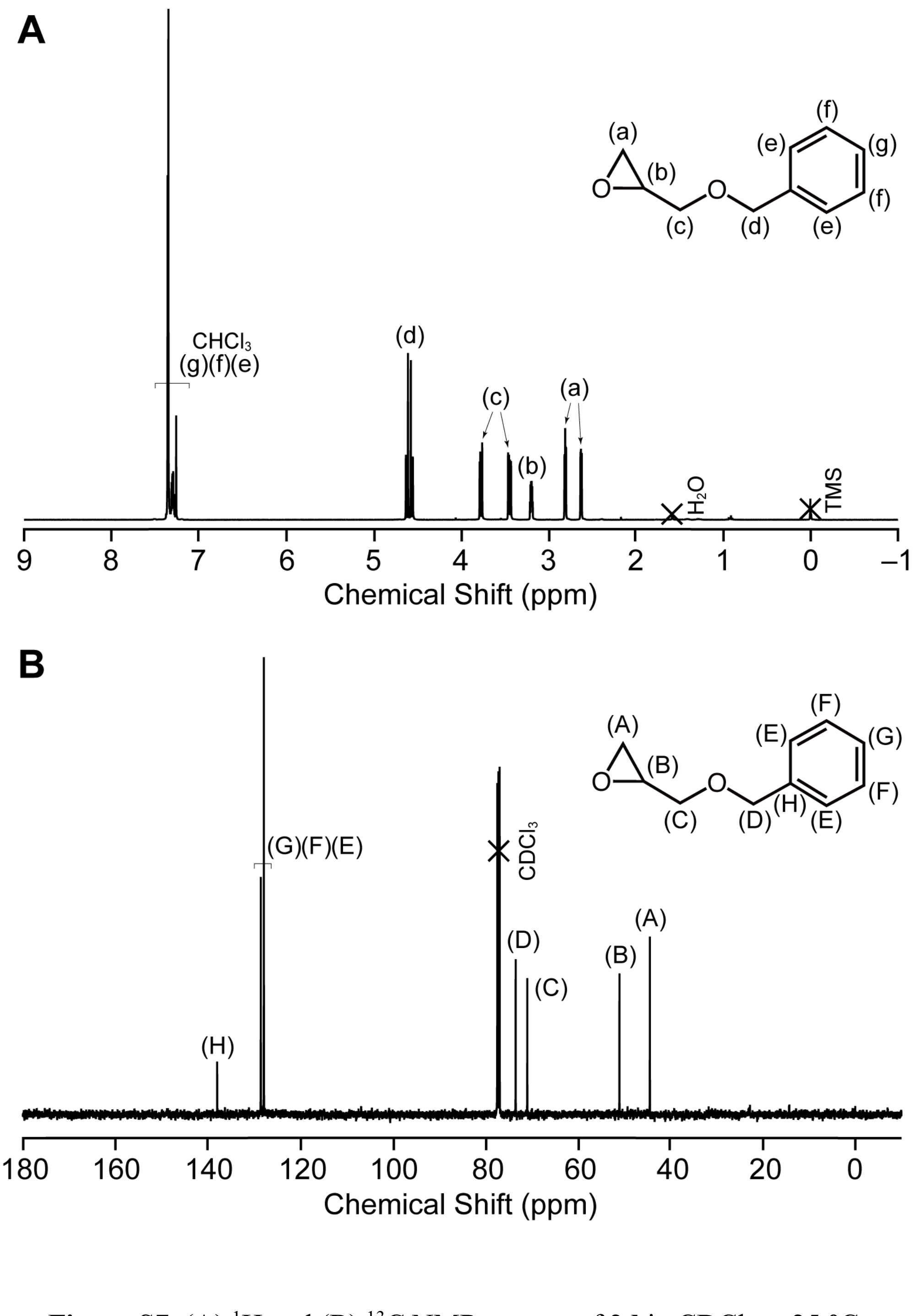

Figure S7. (A) ^{1}H and (B) ^{13}C NMR spectra of **2d** in $CDCl_3$ at 25 °C.

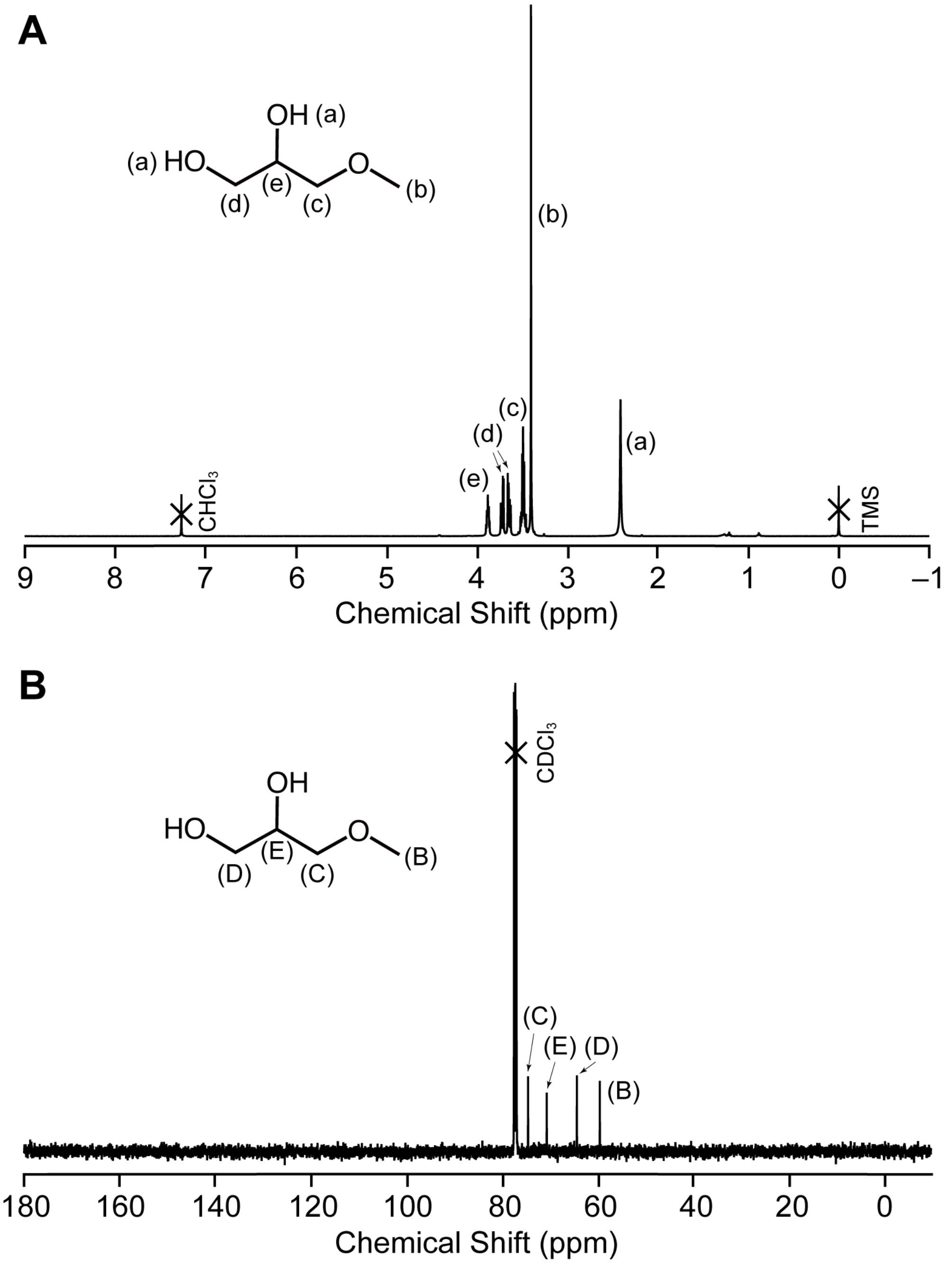

Figure S8. (A) ^{1}H and (B) ^{13}C NMR spectra of **3a** in $CDCl_3$ at 25 °C.

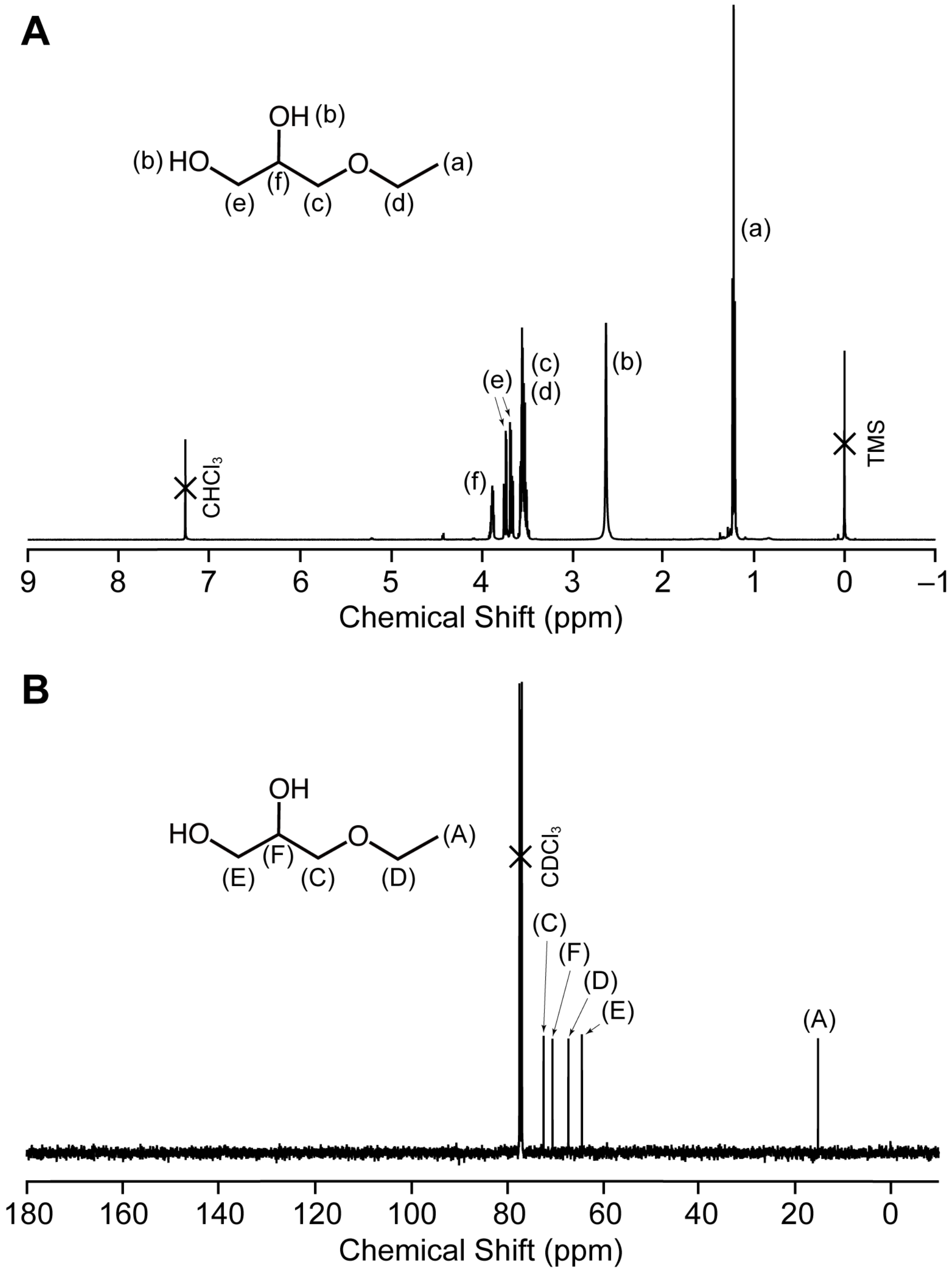

Figure S9. (A) ^{1}H and (B) ^{13}C NMR spectra of **3b** in $CDCl_3$ at 25 °C.

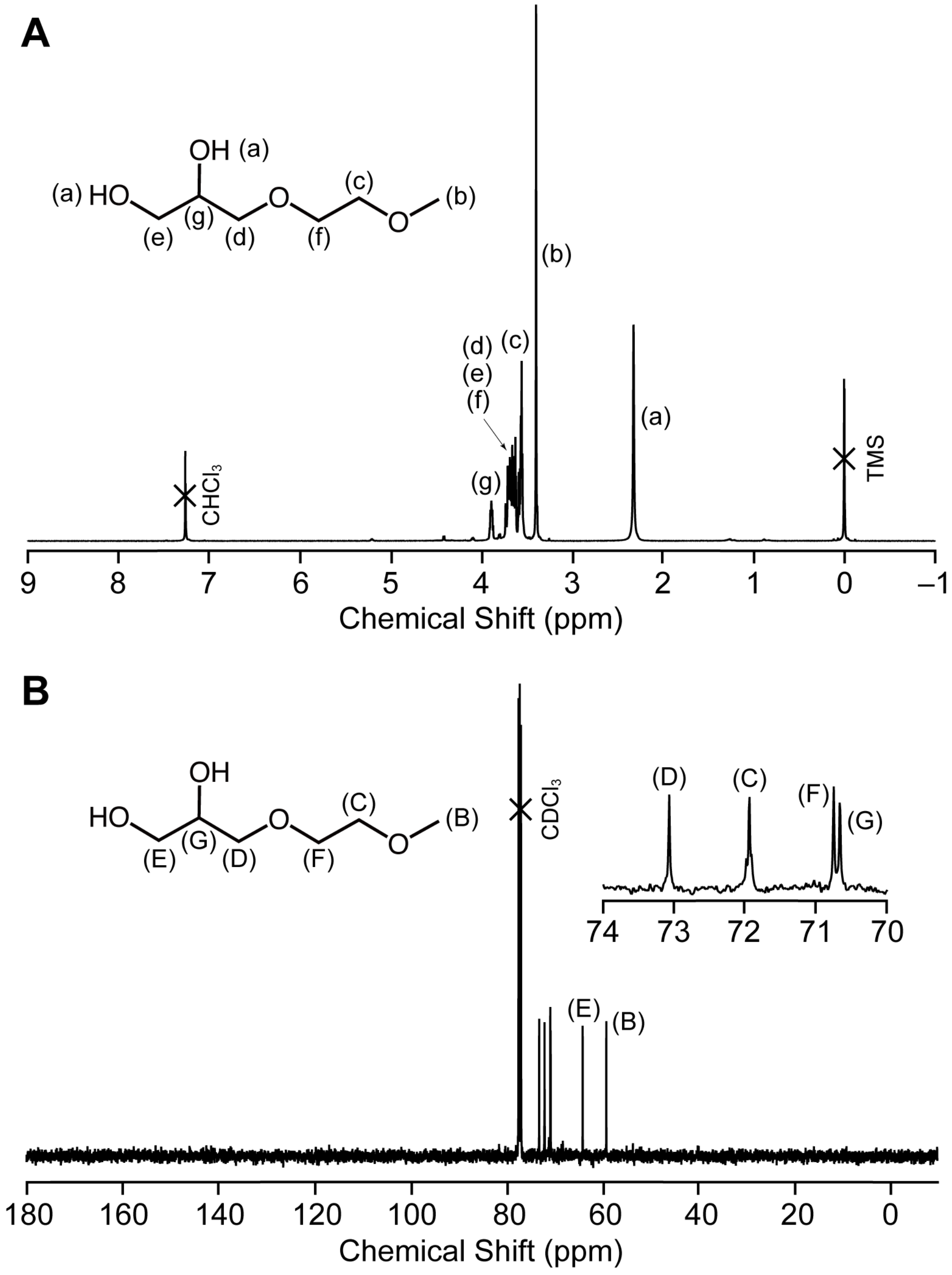

Figure S10. (A) ^{1}H and (B) ^{13}C NMR spectra of **3c** in $CDCl_3$ at 25 °C.

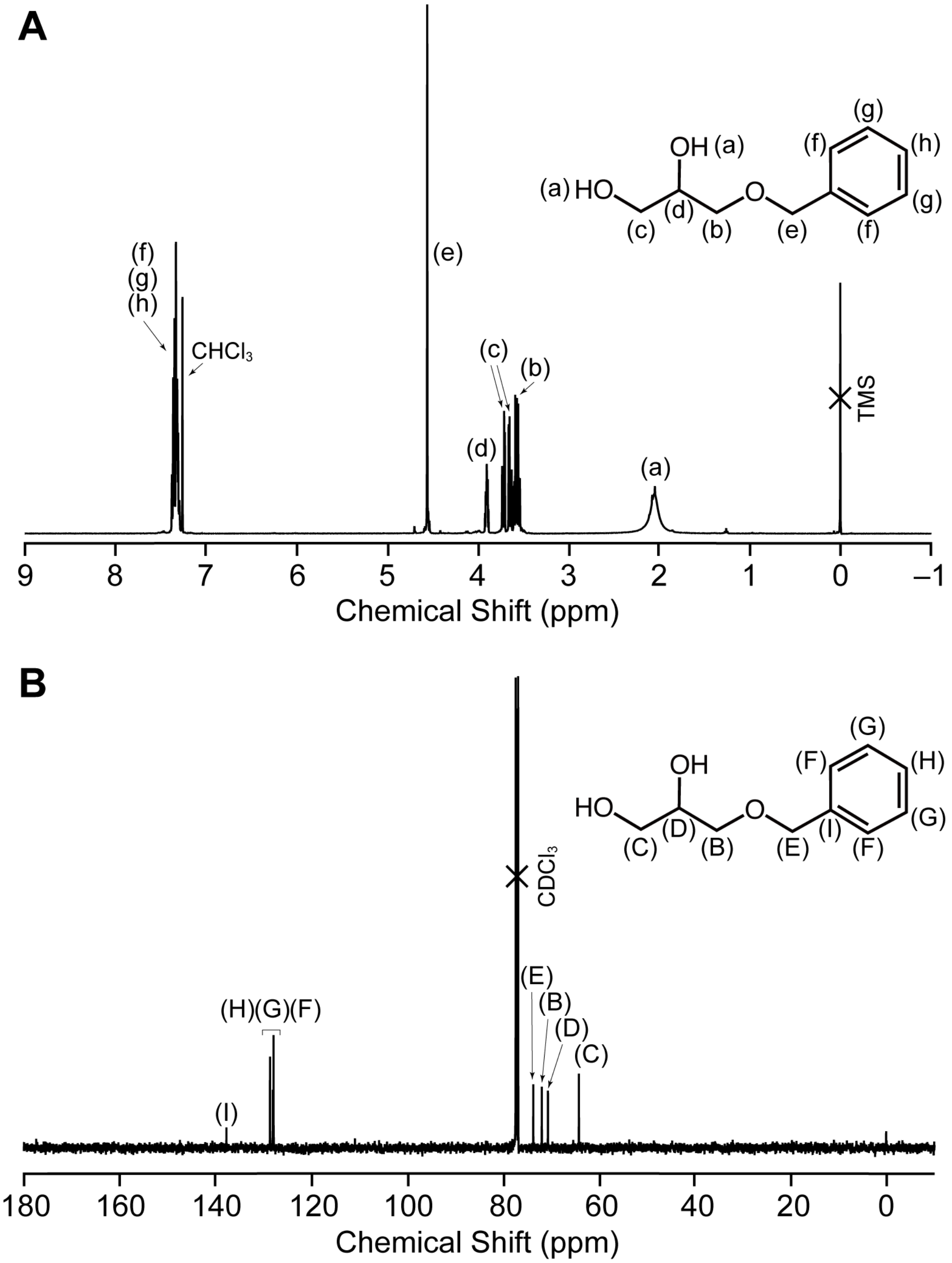

Figure S11. (A) ^{1}H and (B) ^{13}C NMR spectra of **3d** in $CDCl_3$ at 25 °C.

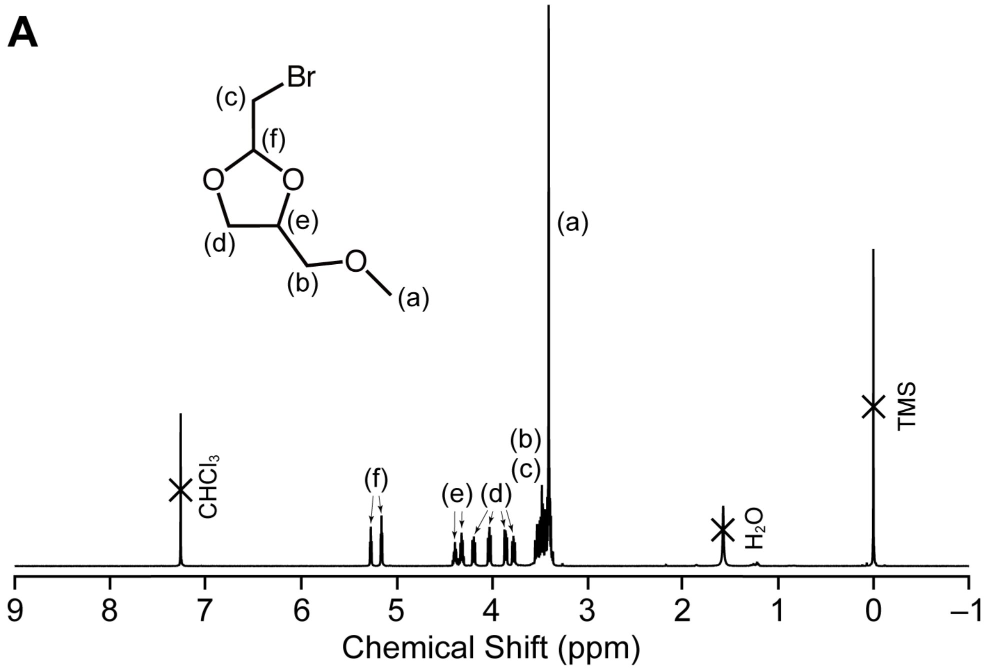

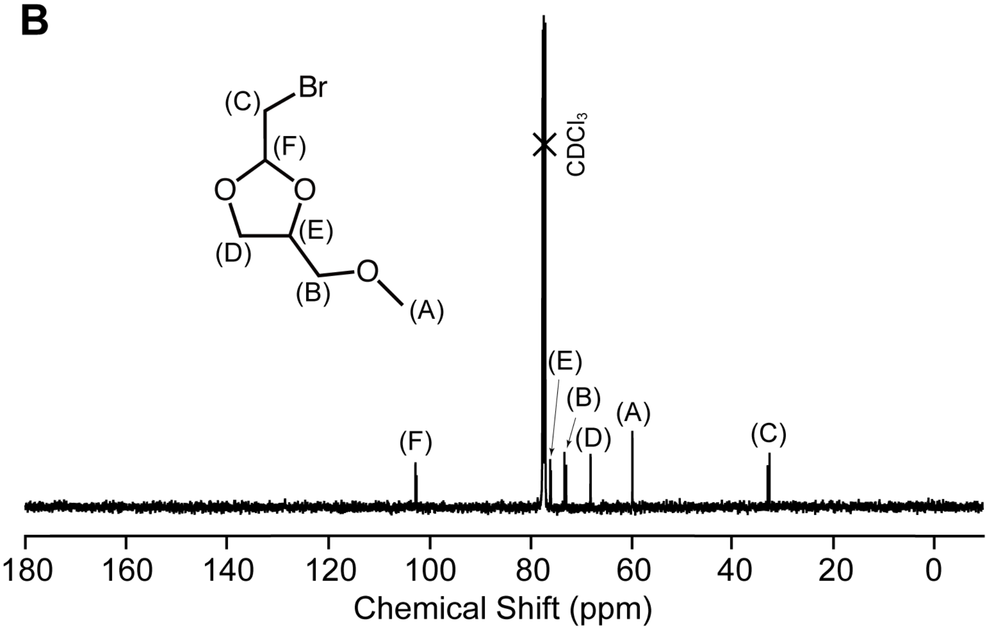

Figure S12. (A) ^{1}H and (B) ^{13}C NMR spectra of **4a** in $CDCl_3$ at 25 °C.

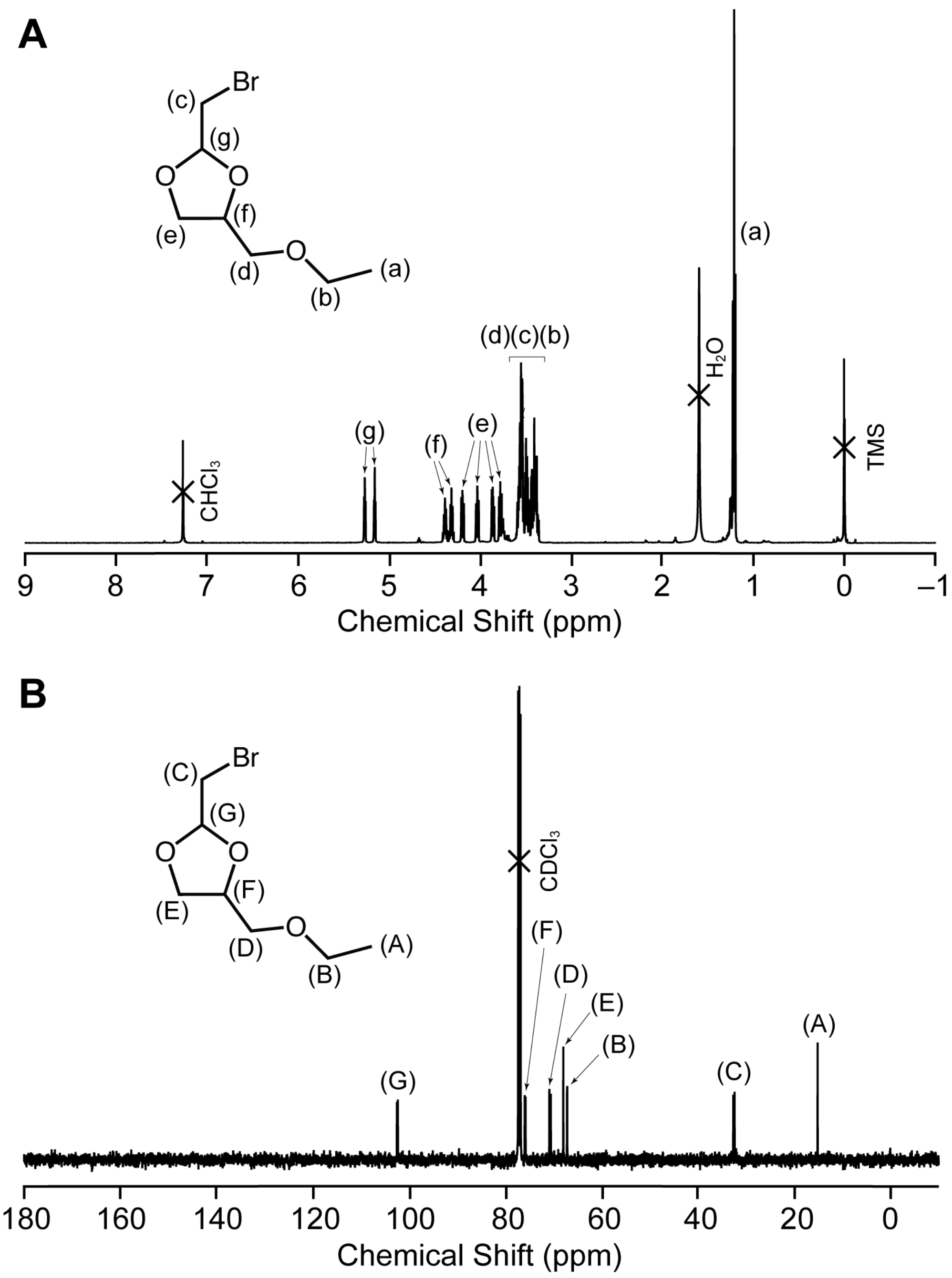

Figure S13. (A) ^{1}H and (B) ^{13}C NMR spectra of **4b** in $CDCl_3$ at 25 °C.

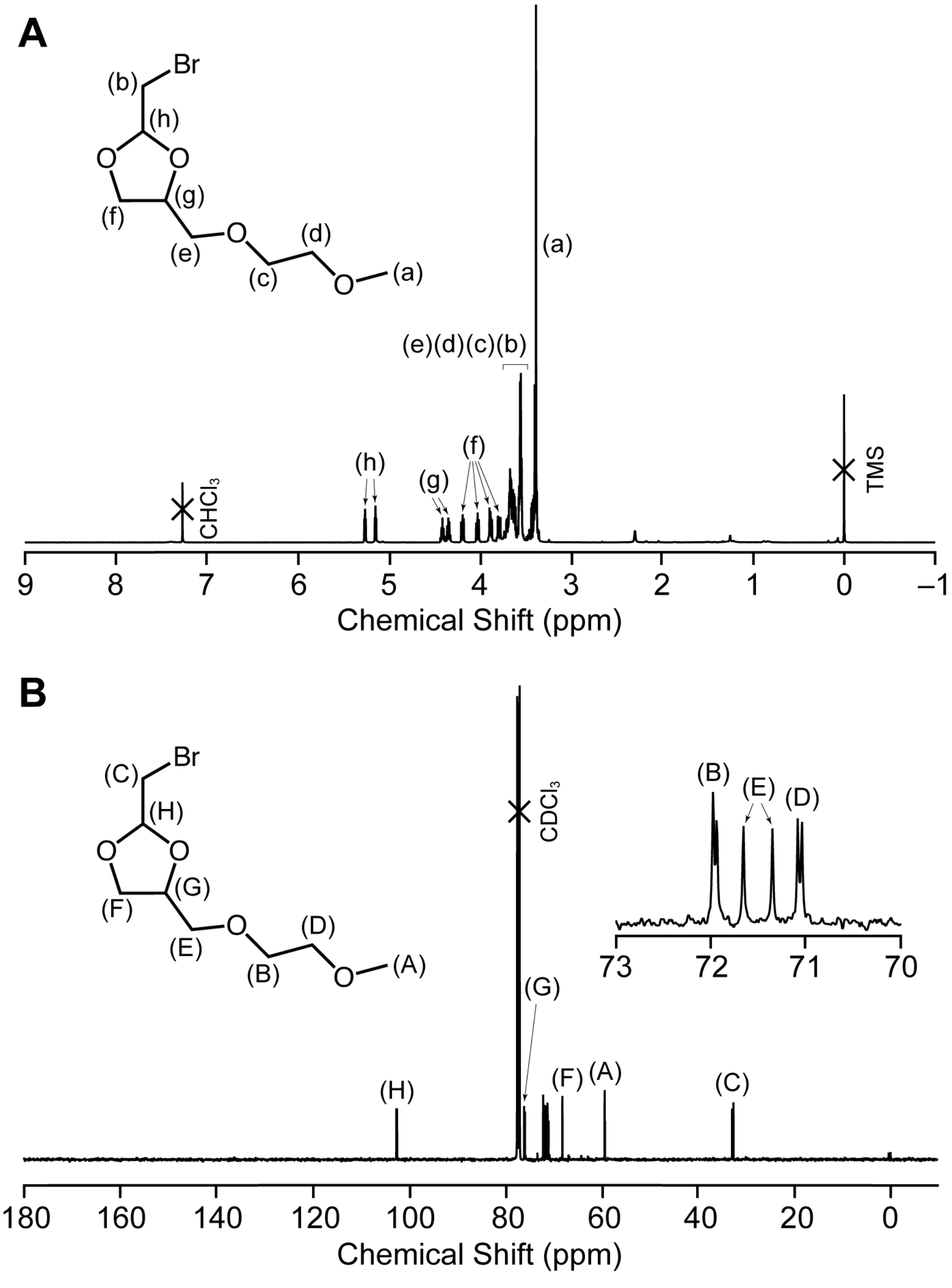

Figure S14. (A) ^{1}H and (B) ^{13}C NMR spectra of **4c** in $CDCl_3$ at 25 °C.

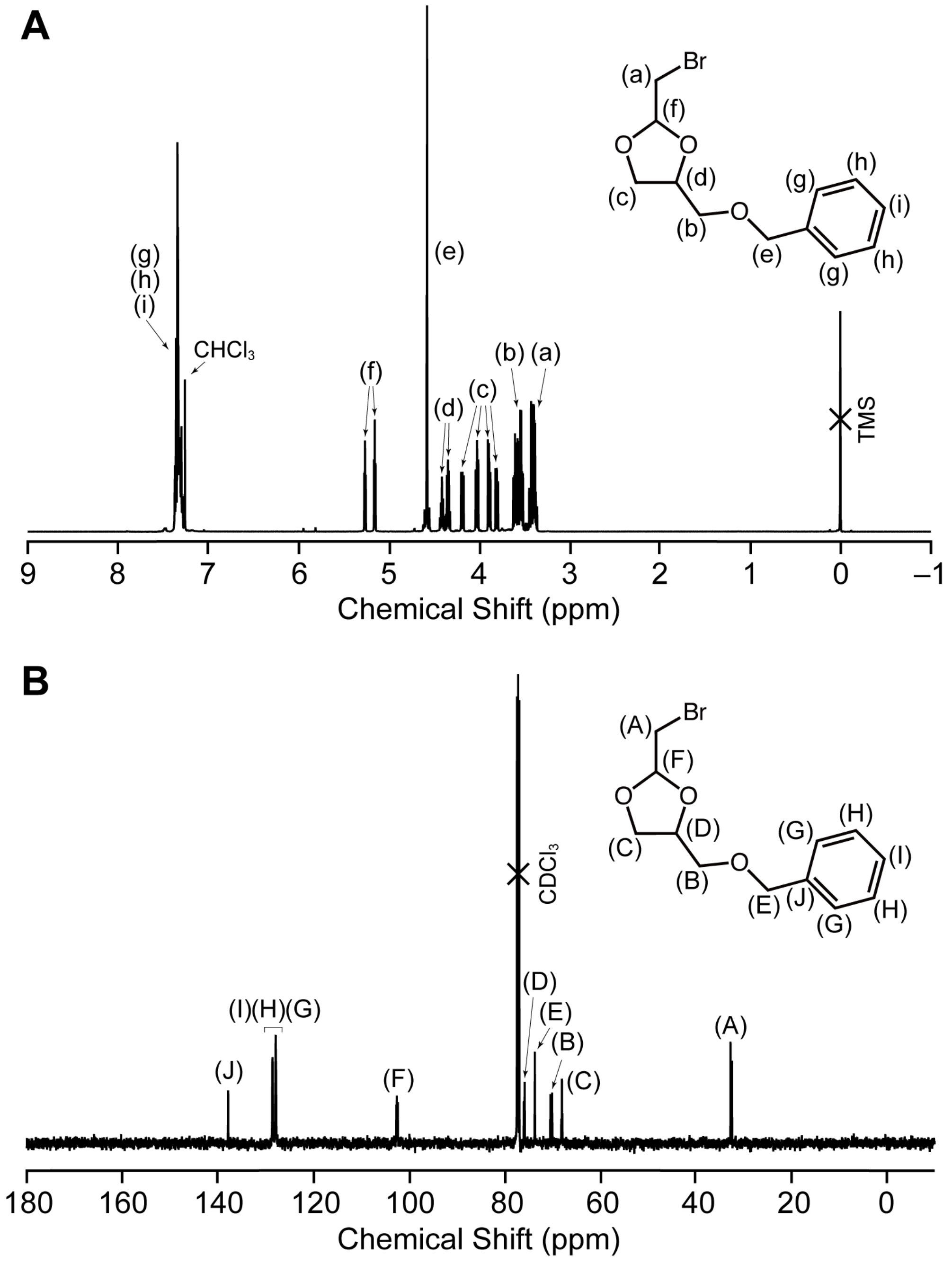

Figure S15. (A) ^{1}H and (B) ^{13}C NMR spectra of **4d** in $CDCl_3$ at 25 °C.

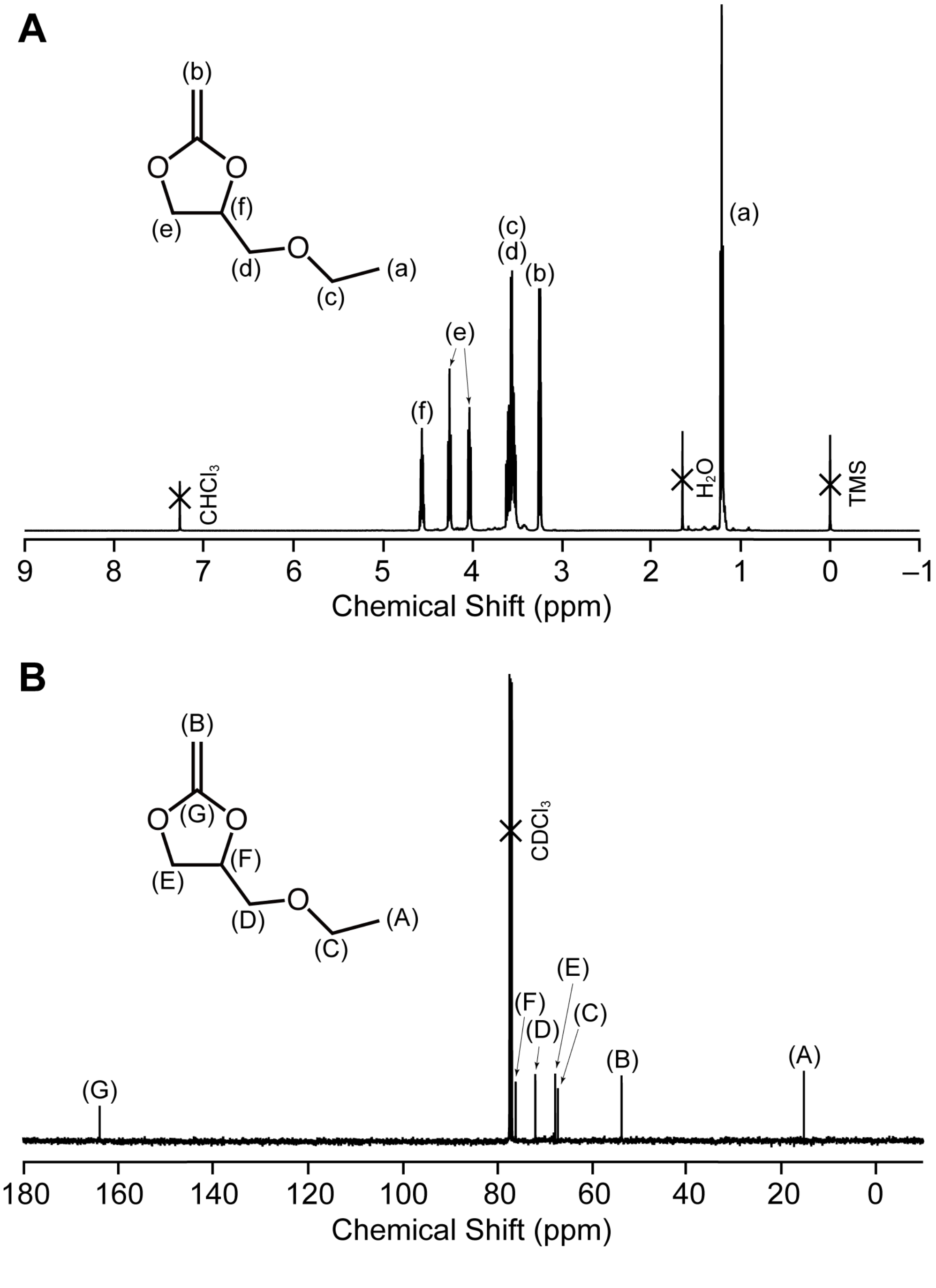

Figure S16. (A) ^{1}H and (B) ^{13}C NMR spectra of **5b** in $CDCl_3$ at 25 °C.

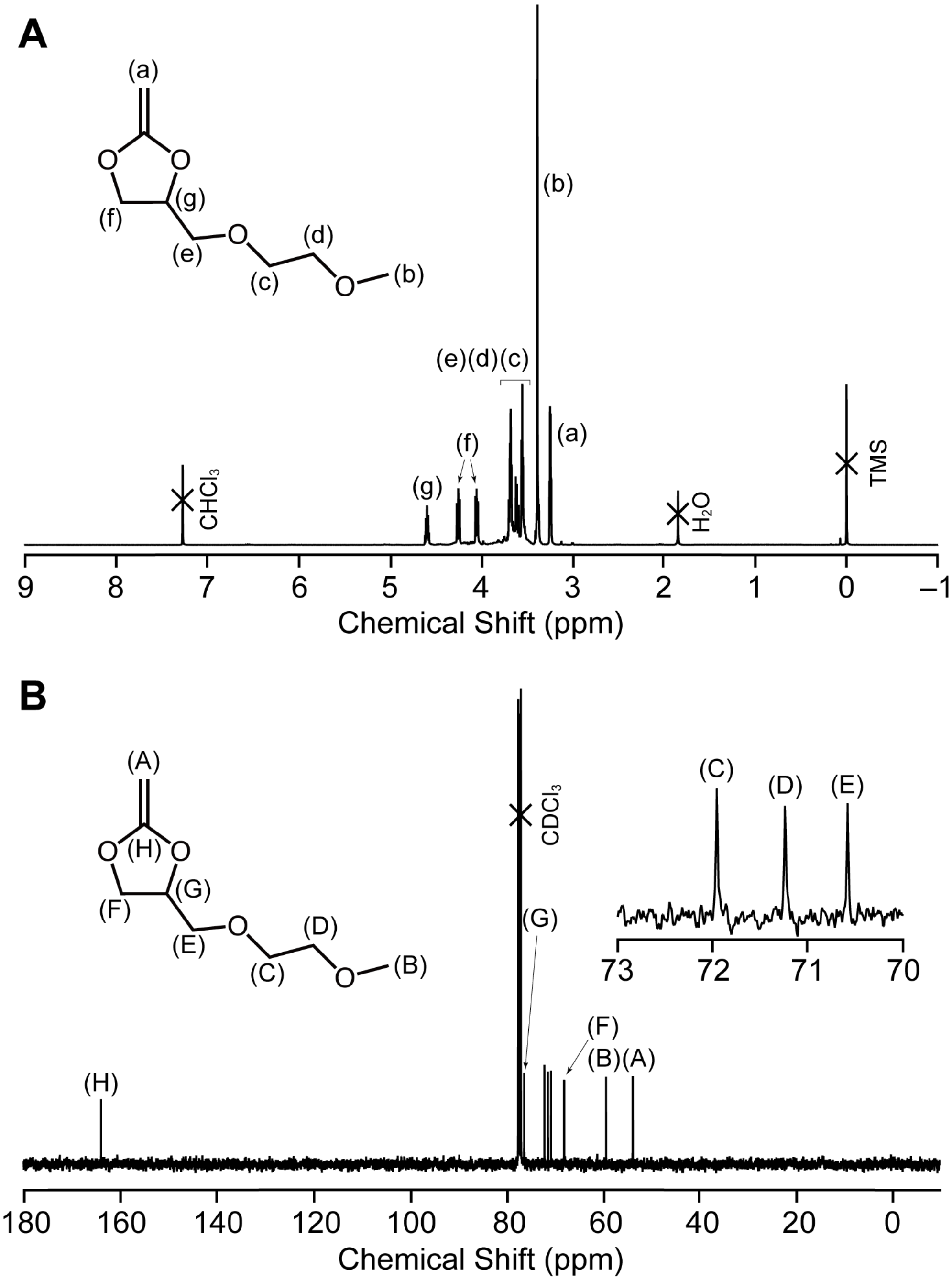

Figure S17. (A) ^{1}H and (B) ^{13}C NMR spectra of **5c** in $CDCl_3$ at 25 °C.

A

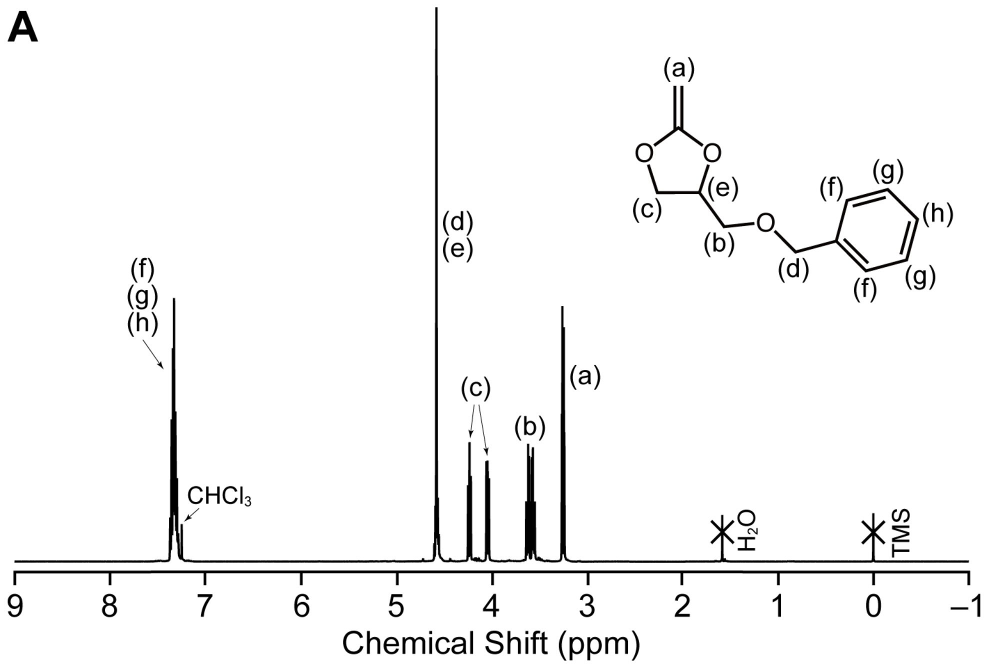

B

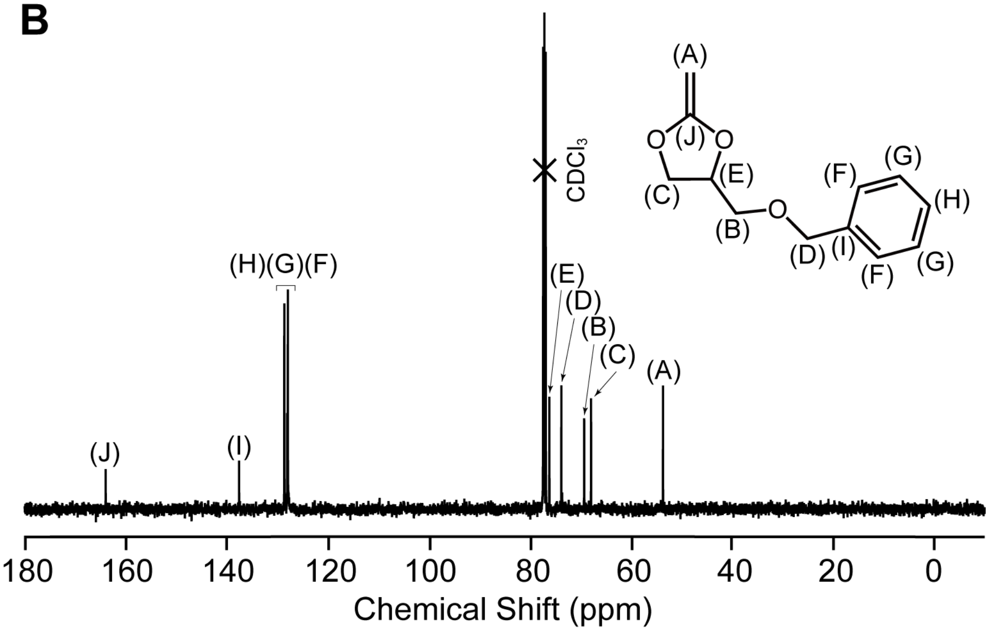

Figure S18. (A) ^{1}H and (B) ^{13}C NMR spectra of **5d** in $CDCl_3$ at 25 °C.

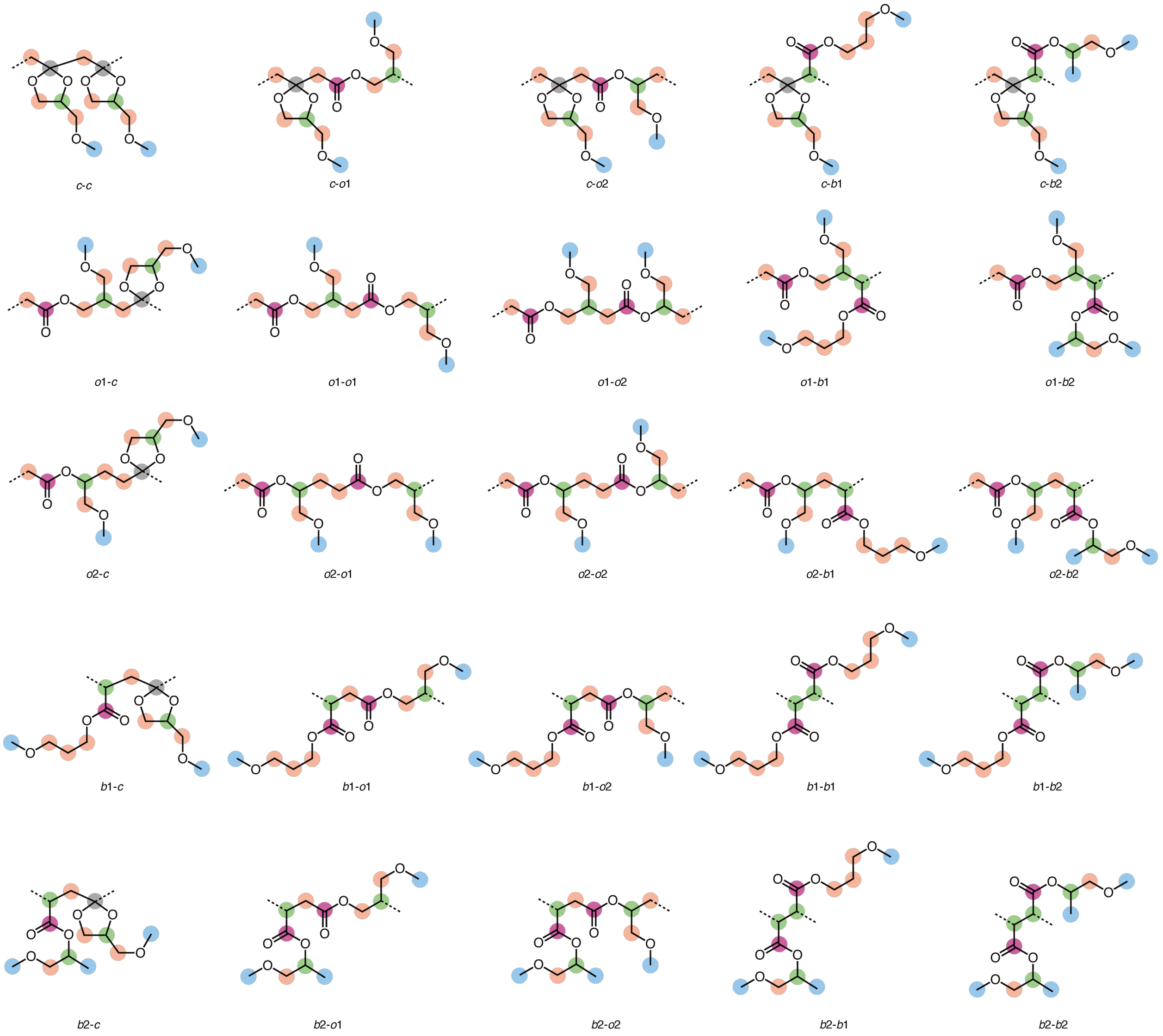

Figure S19. Twenty-five kinds of diad structures of the polymer generated by polymerization of **5a**. Color coding means that blue is 1°, orange is 2°, green is 3°, gray is 4°, and purple is carbonyl carbons.

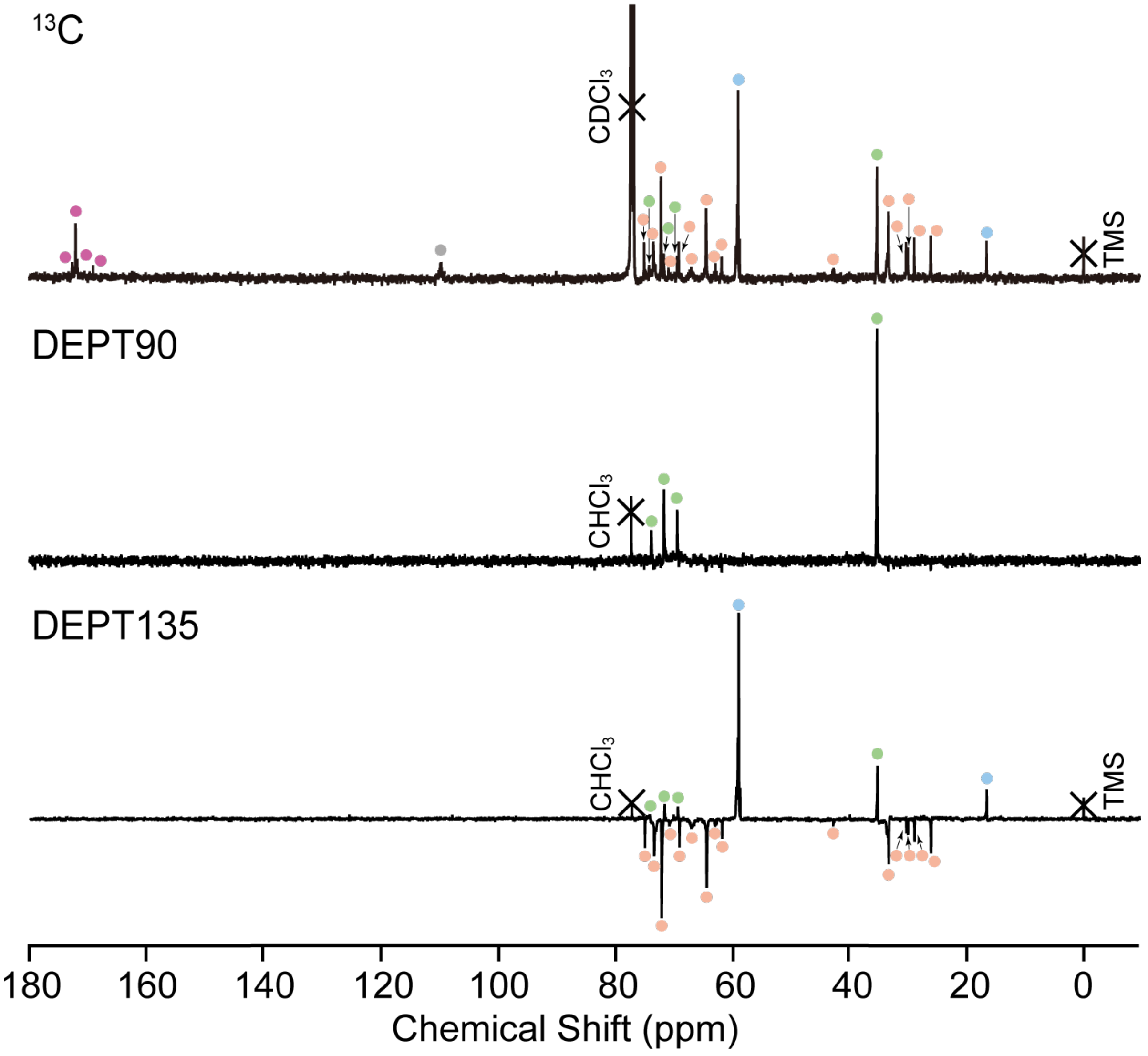

Figure S20. DEPT analyses of the polymer generated by polymerization of **5a**. The spectra were corrected in $CDCl_3$ at 25 °C. Color coding means that blue is 1°, orange is 2°, green is 3°, gray is 4°, and purple is carbonyl carbons.

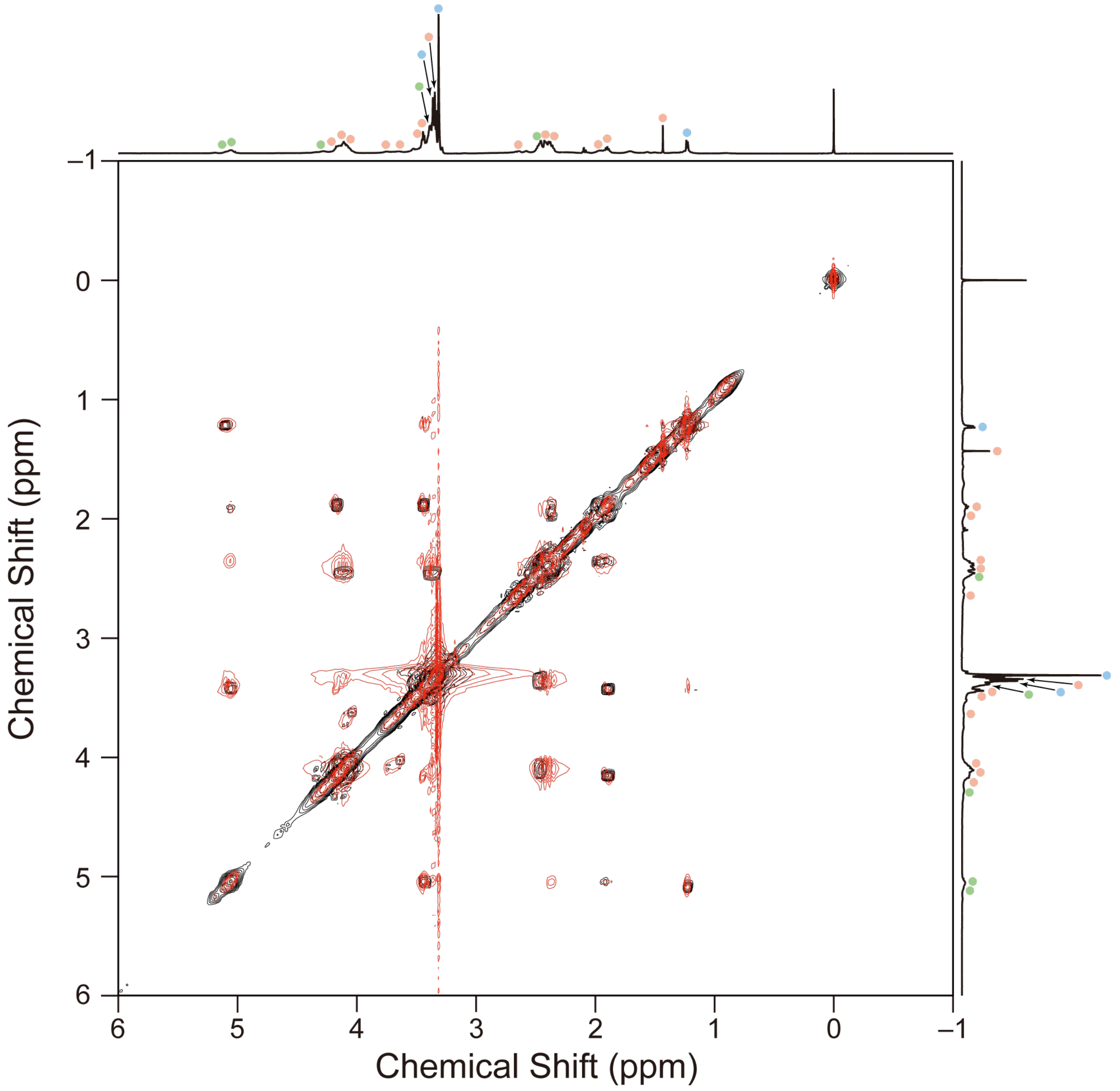

Figure S21. ^{1}H-^{1}H COSY (black) and ^{1}H-^{1}H TOCSY (red) spectra of **5a** in $CDCl_3$ at 25 °C.

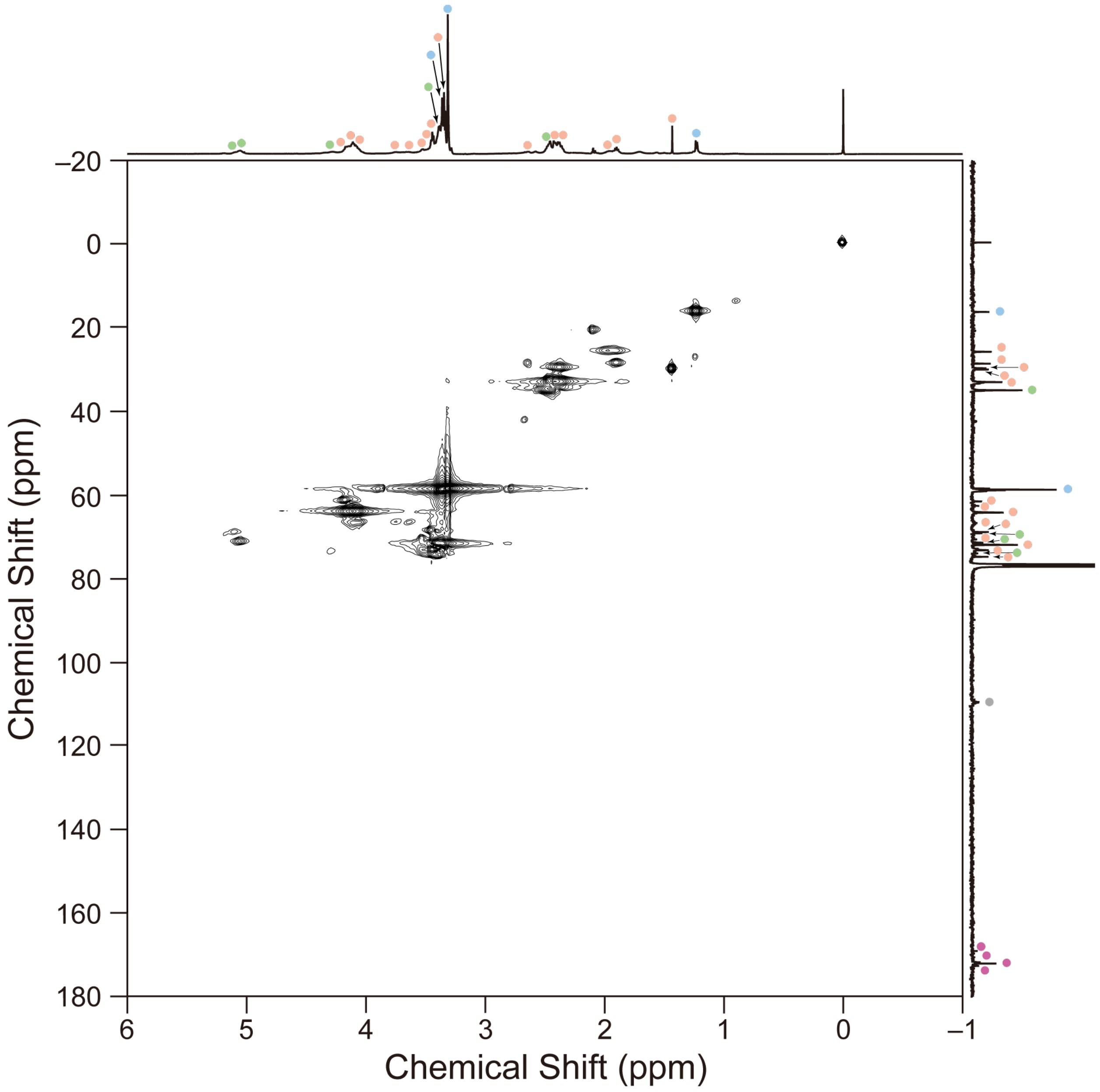

Figure S22. ^{1}H-^{13}C HMQC spectrum of **5a** in $CDCl_3$ at 25 °C.

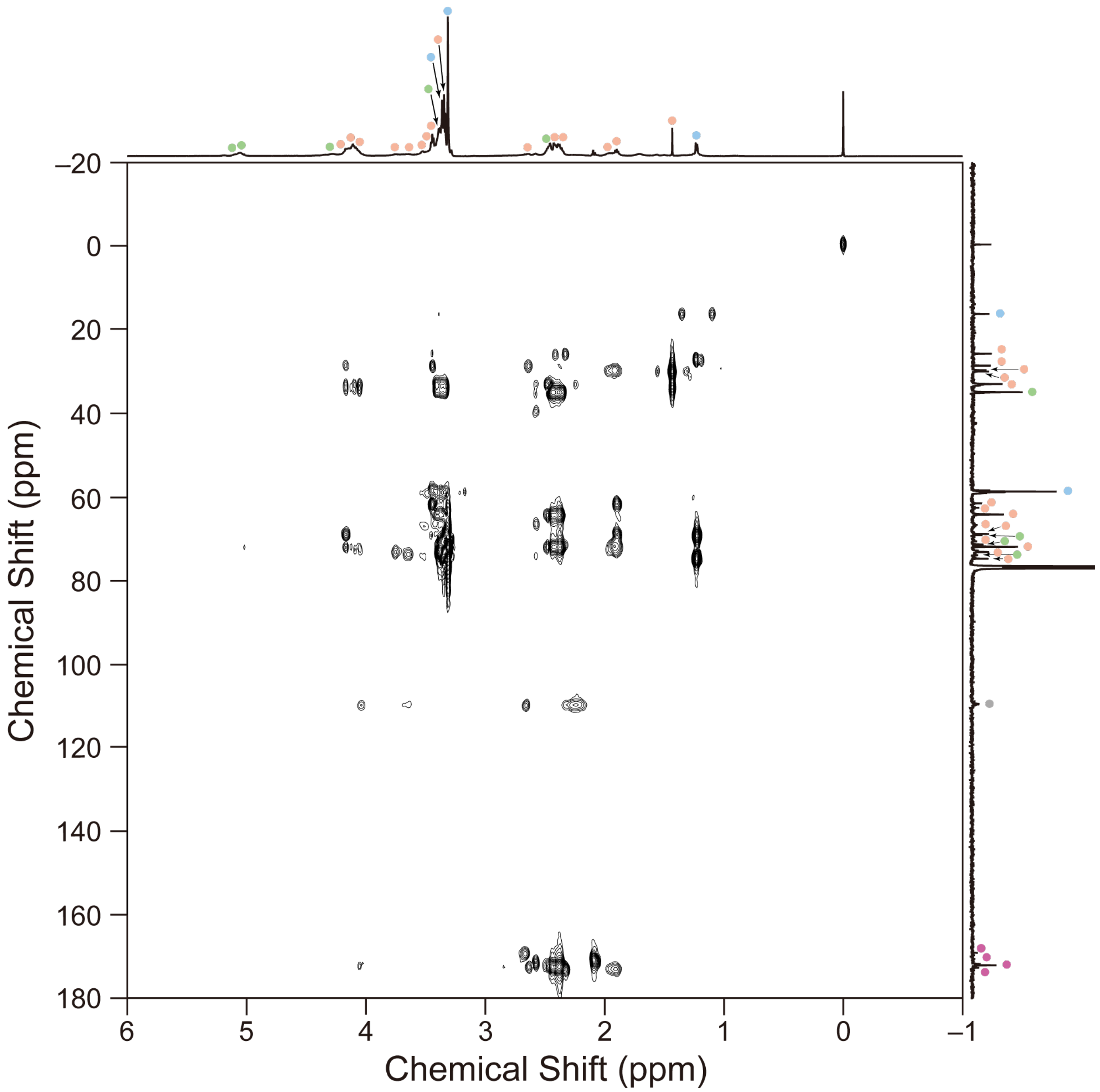

Figure S23. ^{1}H-^{13}C HMBC spectrum of **5a** in $CDCl_3$ at 25 °C.

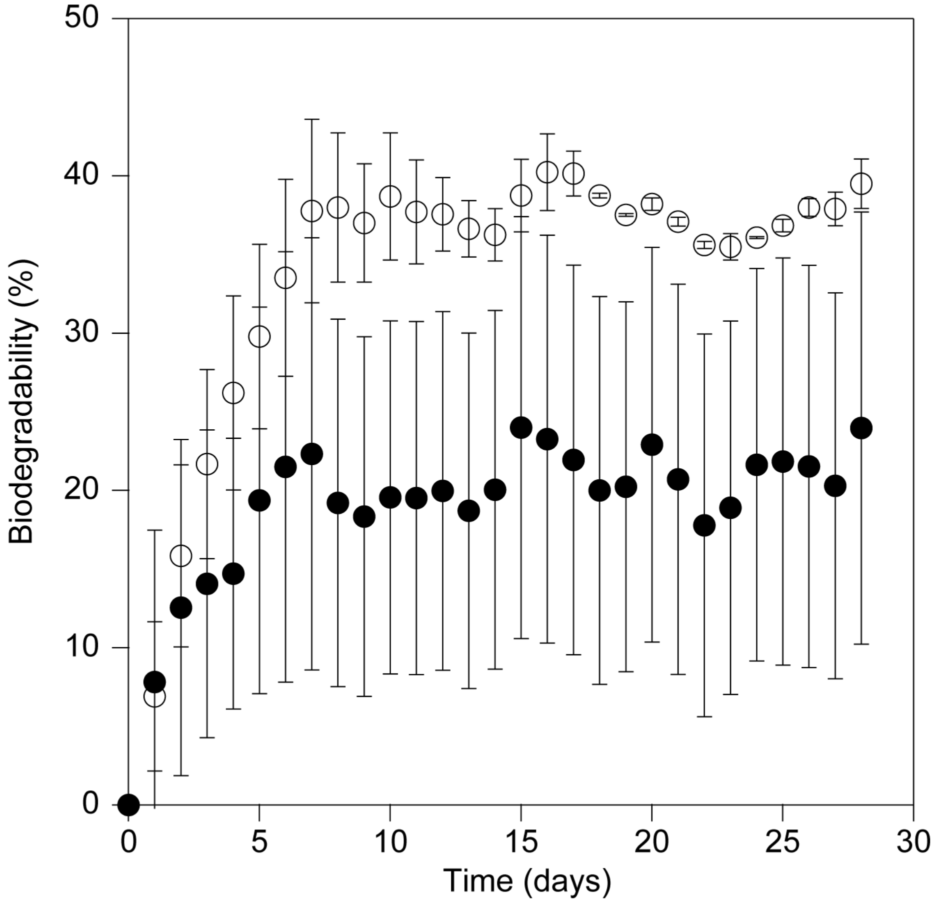

Figure S24. Biodegradability test for the **5a** polymer (black circle) and cellulose (open circle) using river water. The experiment was carried out for 28 days at 22 °C.

Table S1. Assignment of carbon atom of the **5a** polymer.

1° (ppm)	2°(ppm)	3°(ppm)	4°(ppm)	carbonyl (ppm)
16.6	26.1	35.2	109.6	169.9
59.0	28.9	69.4		171.7
	30.0	71.6		172.0
	30.3	74.3		172.6
	33.3			
	42.7			
	61.8			
	63.0			
	64.4			
	66.9			
	69.1			
	70.8			
	72.1			
	73.4			
	75.5			

Table S2. Assignment of proton atom of the C5-COMe polymer.

1° (ppm)	2°(ppm)	3°(ppm)
1.23	1.43	2.45
3.39	1.50	4.29
	1.56	5.05
	1.89	5.10
	1.94	
	2.30	
	2.40	
	2.47	
	2.63	
	2.66	
	3.31	
	3.35	
	3.39	
	3.45	
	3.52	
	3.63	
	3.74	
	4.09	
	4.11	
	4.17	

Table S3. Summary of the polymerization of **5b-5d** in this study. The polymerization were carried out with a monomer concentration of 2.4 M.[a)]

Monomer	Temp. (°C)	$\bar{R}_c$ [b)] ×10^2	$\bar{R}_{op1}$ [b)] ×10^2	$\bar{R}_{op2}$ [b)] ×10^2	$\bar{R}_{o1}$ [b)] ×10^2	$\bar{R}_{o2}$ [b)] ×10^2	$\bar{R}_{b1}$ [b)] ×10^2	$\bar{R}_{b2}$ [b)] ×10^2
**5b**	100	3.6	70.1	26.3	64.5	18.2	5.6	8.1
	140	1.2	70.5	28.3	53.6	8.3	16.9	20.0
**5c**	100	4.8	69.0	26.2	60.8	16.3	8.2	9.9
	140	0.9	71.1	28.0	50.0	8.7	21.1	19.3
**5d**	100	2.9	71.3	25.8	68.0	18.5	3.3	7.3
	140	1.5	69.3	29.2	49.8	11.0	19.5	18.2

a) The polymerizations were carried out in *o*-dichlorobenzene.
b) calculated from ^{1}H NMR analyses.

The 3D Structures and Cartesian Coordinates for The Transition States in The Reactions

reaction (i)

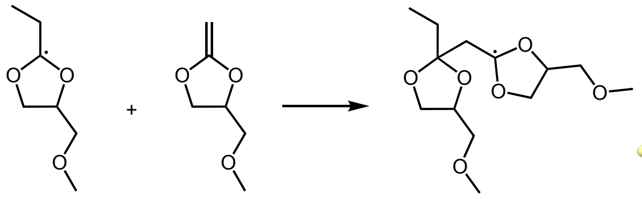

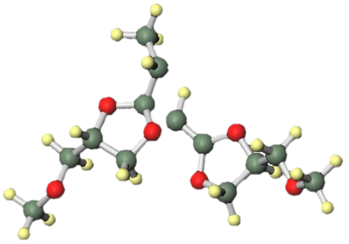

	x	y	z
C	0.64550069	2.44293342	1.03361003
C	1.63959095	1.29688568	1.24204574
C	1.63760536	0.28154083	0.12887562
C	2.56462611	1.22792594	-1.70869872
C	3.86415723	0.82317534	-1.79899648
O	4.27445934	-0.35197259	-2.37529658
C	5.51074046	-0.71878984	-1.74667801
C	6.07808883	0.6350561	-1.30798064
C	6.8613614	0.60072211	-0.00615404
O	7.99128927	-0.21936935	-0.22888121
C	8.81094771	-0.34859035	0.91549163
O	4.89590338	1.42430683	-1.11738255
O	2.45650674	-0.8099171	0.39886129
C	1.94583923	-1.89794882	-0.37365327
C	0.43749892	-1.61592314	-0.40267496
C	-0.25357567	-2.00065775	-1.70197208
O	-0.16046036	-3.40683364	-1.82318702
C	-0.76224361	-3.89709167	-3.00330838
O	0.36659377	-0.19555146	-0.2253063
H	0.67593155	3.13494242	1.88207783
H	0.86992597	3.01476009	0.1270012
H	-0.37512986	2.05887944	0.94199091
H	2.66184606	1.68296004	1.3339237
H	1.41092955	0.77867792	2.18903386
H	1.84570306	0.77598526	-2.38148005
H	2.38527032	2.25857979	-1.42708411
H	5.31134908	-1.3663863	-0.88292905
H	6.13022686	-1.24577876	-2.47302601
H	6.68895522	1.08199799	-2.10375948
H	7.15879954	1.62103367	0.28502417
H	6.22060636	0.19513962	0.79407141
H	9.64914048	-0.99315796	0.63936261
H	9.20170043	0.62679877	1.24557456
H	8.26571839	-0.80710791	1.75546336
H	2.37965933	-1.88587592	-1.38191197
H	2.20701984	-2.8314359	0.1277594
H	-0.07023237	-2.10698144	0.43857898
H	-1.30539026	-1.67153568	-1.68356822
H	0.24070686	-1.49506892	-2.54829534
H	-0.63950828	-4.9832635	-3.0007281
H	-1.83702586	-3.65755922	-3.04015558
H	-0.28390395	-3.4840368	-3.9057055

reaction (ii)

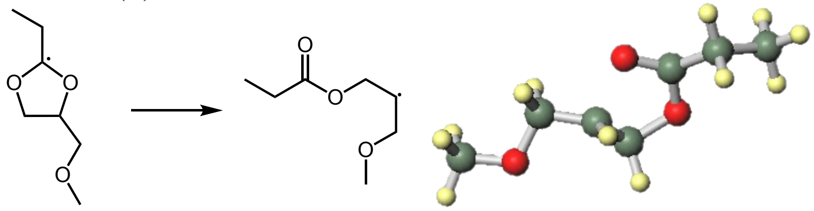

	x	y	z
C	3.22541948	1.43976302	-0.68693184
C	1.85350429	1.15737153	-0.06306249
C	1.16277943	-0.04466712	-0.65439337
O	1.88567303	-1.19558052	-0.67374695
C	0.98928027	-2.33028221	-0.41321152
C	-0.26248197	-1.79881388	0.21338701
C	-1.58776701	-2.32053464	-0.24719194
O	-1.69455629	-3.6877757	0.13818349
C	-2.9195606	-4.26728615	-0.26426812
O	-0.11130452	-0.21266445	-0.63800262
H	3.69348068	2.30793616	-0.21122577
H	3.89229303	0.58106232	-0.56477699
H	3.13463528	1.64537004	-1.75906971
H	1.97079084	0.99622249	1.02387342
H	1.18382805	2.01444702	-0.18498591
H	0.78043698	-2.82989593	-1.36508477
H	1.55387822	-3.00309683	0.23528119
H	-0.19396505	-1.55189744	1.27150223
H	-2.4079759	-1.73068556	0.19198791
H	-1.65823246	-2.22302635	-1.34326985
H	-2.90866535	-5.30780235	0.0702554
H	-3.78207213	-3.75376903	0.19023872
H	-3.04217152	-4.24363107	-1.35918961

reaction (iii)

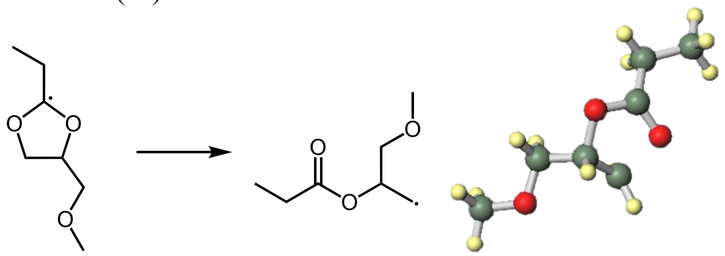

	x	y	z
C	3.21471819	1.49979617	-0.67287413
C	1.74039752	1.10976174	-0.54057194
C	1.54306739	-0.19347754	0.19208222
O	2.2551284	-1.23936351	-0.00496994
C	0.9847398	-2.45061243	-0.47611354
C	-0.02747403	-1.87596836	0.46181162
C	-1.46888479	-1.92518024	-0.02707109
O	-1.86182797	-3.28176318	-0.07610242
C	-3.20284386	-3.44653883	-0.48867694
O	0.27925042	-0.43803982	0.62663921
H	3.32077254	2.41996217	-1.2569051
H	3.6678473	1.6638327	0.31065943
H	3.77936683	0.70623464	-1.17145519
H	1.1695555	1.88653224	-0.01964697
H	1.28484674	1.01659101	-1.54340303
H	0.81866678	-2.29866497	-1.54076908
H	1.45586118	-3.39093854	-0.21778871
H	0.0490211	-2.34096234	1.4499427
H	-2.11353117	-1.35170377	0.65872839
H	-1.53668738	-1.45307372	-1.02199682
H	-3.40931833	-4.51976081	-0.48909437
H	-3.90435499	-2.94629254	0.1980812
H	-3.36864528	-3.04907904	-1.5029026

reaction (iv)

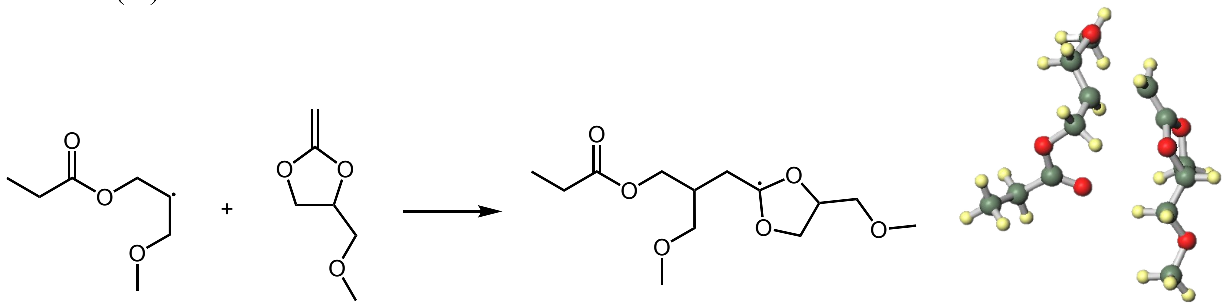

	x	y	z
C	7.29188395	2.82798405	0.18920299
C	6.36143515	2.41197657	-0.96058495
C	5.14347224	1.63256932	-0.49547309
O	5.500802	0.5223684	0.17240844
C	4.39650475	-0.33888291	0.65925358
C	3.95549645	-1.33124862	-0.35728818
C	1.85842019	-1.89203956	0.3648475
C	1.09003747	-0.78464577	0.17030526
O	0.53734977	-0.43691411	-1.02271584
C	0.33197239	0.98887856	-1.00013955
C	0.20290623	1.28669205	0.49634303
C	0.79696353	2.61795471	0.92657962
O	0.01091833	3.62020335	0.30937628
C	0.49003588	4.92546185	0.56572189
O	0.95878571	0.21571979	1.09430186
C	4.72358547	-2.61703638	-0.48761945
O	4.06632669	-3.614719	-1.2643184
C	4.14992533	-3.40201948	-2.65868368
O	3.99193916	1.97233181	-0.70418586
H	8.14695774	3.39183203	-0.19828062
H	6.76795676	3.46572709	0.90974348
H	7.66927821	1.95109892	0.72212775
H	5.99337618	3.28455968	-1.50683211
H	6.9144452	1.78086778	-1.66824759
H	4.8243859	-0.81836792	1.54447687
H	3.58438398	0.32446488	0.95813176
H	3.59532504	-0.8914534	-1.28510634
H	1.8269513	-2.68838787	-0.36594025
H	2.1129728	-2.15809859	1.38385354
H	1.20455791	1.48994485	-1.43538949
H	-0.56383284	1.21849778	-1.57727235
H	-0.83981715	1.21082947	0.82960558
H	0.76736274	2.70850812	2.0248355
H	1.84711322	2.67250688	0.60212107
H	-0.17564583	5.61928753	0.04613125
H	0.47803808	5.15634697	1.64282391
H	1.51623038	5.06059922	0.19122148
H	5.73448318	-2.44190332	-0.91163089
H	4.87471379	-3.06670631	0.502789
H	5.19789058	-3.31983716	-2.99107398
H	3.69390697	-4.27146648	-3.14040768
H	3.61157133	-2.49957054	-2.9844563

reaction (v)

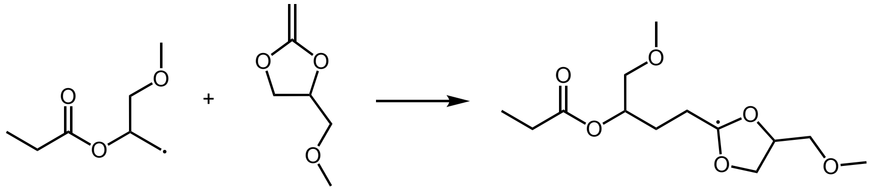

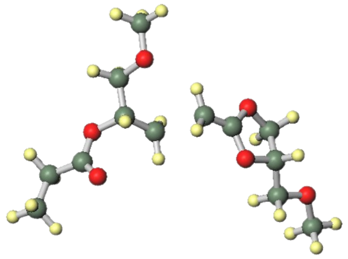

	x	y	z
C	-2.64758919	8.00745882	0.28206524
C	-2.99763276	6.83745942	1.19927289
C	-2.99014542	5.49855045	0.47897366
O	-3.33277949	4.50345237	1.32244574
C	-3.34468521	3.12239132	0.76599748
C	-4.38750834	2.42442468	1.63853224
O	-4.69756482	1.16298627	1.07877056
C	-5.66730345	0.46179313	1.82828045
C	-1.97934531	2.53076246	0.80398253
C	-1.95642315	0.59550504	-0.47697884
C	-0.75527816	-0.02045577	-0.33983111
O	-0.46385713	-0.94326679	0.62368695
C	0.9630997	-1.07215433	0.69212911
C	1.4146355	-0.5910319	-0.69004291
C	2.73462068	0.16152009	-0.70532102
O	3.72700455	-0.75241635	-0.28660717
C	5.02223971	-0.18506934	-0.2592625
O	0.35947218	0.30782478	-1.06360963
O	-2.71178957	5.34224216	-0.69204717
H	-2.66092922	8.94960818	0.84024582
H	-1.65352579	7.87798092	-0.15592919
H	-3.36023512	8.08528542	-0.54451266
H	-2.29509554	6.76459787	2.0397991
H	-3.98687346	6.96831472	1.65629724
H	-3.71213961	3.205644	-0.26047774
H	-4.0008212	2.31229532	2.66558346
H	-5.29036089	3.05347159	1.69099821
H	-6.61585041	1.01978378	1.88709236
H	-5.84428012	-0.48846864	1.31804718
H	-5.32088141	0.25909612	2.85455349
H	-1.22492215	3.04361927	0.21576176
H	-1.63725023	2.13752607	1.75913632
H	-2.80944286	0.21331973	0.0645746
H	-2.13724857	1.1526941	-1.38710216
H	1.35687019	-0.43209904	1.49319561
H	1.21130814	-2.11333108	0.90144459
H	1.44925113	-1.42443492	-1.40407352
H	2.9412073	0.54408049	-1.71751522
H	2.67186501	1.02751445	-0.02554398
H	5.70867768	-0.96881521	0.06992939
H	5.32974752	0.16729982	-1.25596832
H	5.07917826	0.66146946	0.44256143

reaction (vi)

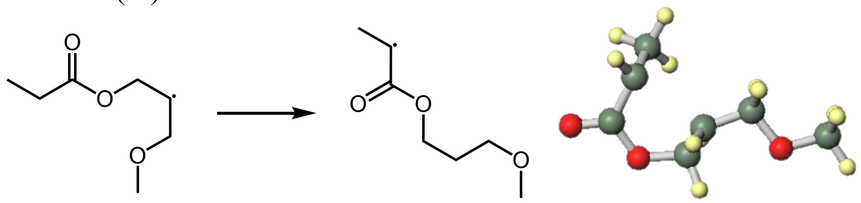

	x	y	z
C	-1.69877104	1.42628123	-2.42055521
C	-0.81932173	1.02258045	-1.24832236
C	-0.85335919	1.93776696	-0.05131146
O	-1.81955574	1.71488294	0.88353332
C	-2.75663499	0.62231154	0.74090163
C	-2.09033318	-0.65362564	0.27434043
C	-2.99919137	-1.71320187	-0.2875948
O	-3.91169372	-2.11288933	0.73041708
C	-4.80679802	-3.11687852	0.29602467
O	-0.06218228	2.83665072	0.11996152
H	-1.63963733	0.68609885	-3.22524443
H	-2.75063729	1.53459084	-2.13264727
H	-1.37101182	2.39182037	-2.82847722
H	-1.33726279	-0.09751942	-0.70875594
H	0.21300052	0.8060681	-1.52878394
H	-3.20498309	0.51318184	1.73210186
H	-3.55312859	0.92043311	0.04595687
H	-1.34605498	-1.0302407	0.98097531
H	-3.5591376	-1.32398819	-1.15690356
H	-2.41337309	-2.57939544	-0.63674114
H	-5.45685046	-3.35578666	1.14149338
H	-5.4275172	-2.77371715	-0.5475544
H	-4.27398701	-4.02969393	-0.01558884

reaction (vii)

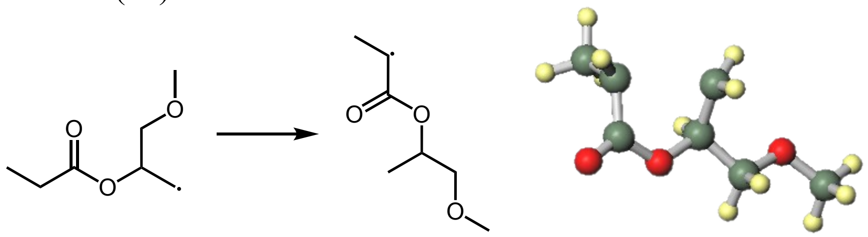

	x	y	z
C	-0.41161729	1.75442362	-2.70058732
C	-0.07644319	1.11027084	-1.36410444
C	-0.29746034	1.96814426	-0.14651754
O	-1.55302328	1.9786222	0.39122199
C	-2.61810318	1.14757821	-0.14244283
C	-3.68879908	1.15595363	0.94959896
O	-4.81166929	0.46410297	0.4464197
C	-5.88072805	0.41373316	1.36913901
C	-2.12240663	-0.22976855	-0.5095745
O	0.56966667	2.62738616	0.378477
H	-0.25811592	1.04565552	-3.52087365
H	-1.44948969	2.10327356	-2.74272907
H	0.23510509	2.62333047	-2.8808735
H	-0.96943748	0.15107766	-1.14424095
H	0.91833601	0.66213897	-1.33269899
H	-3.03207385	1.64291494	-1.02975195
H	-3.29072171	0.67249757	1.85712203
H	-3.94119934	2.19739932	1.20686035
H	-6.69465935	-0.13434237	0.88786156
H	-5.59374381	-0.10922914	2.29517535
H	-6.23508088	1.42208633	1.63572616
H	-2.74771953	-0.77689891	-1.21289729
H	-1.78021811	-0.83383482	0.33368872

reaction (viii)

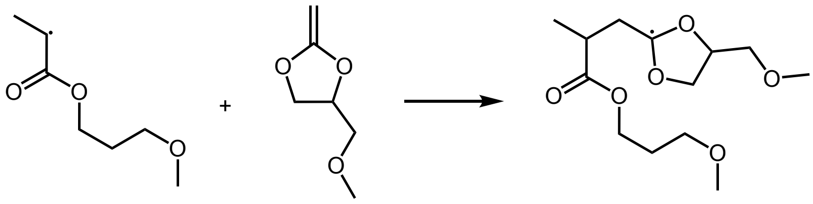

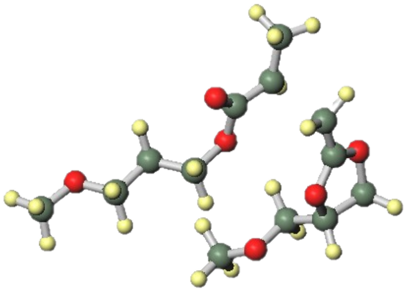

	x	y	z
O	-0.84224526	3.66663938	1.65209919
C	-0.47846507	2.97308046	0.71039
C	0.71993715	3.18616461	-0.08183366
C	2.07892846	1.85017202	1.03044166
C	1.9818121	0.55619691	0.59154756
O	2.59717866	0.11443812	-0.54145582
C	2.3480514	-1.29225088	-0.65110477
C	1.078082	-1.49380426	0.19059246
C	-0.20716672	-1.47734553	-0.62896376
O	-1.28060378	-1.87065846	0.19875821
C	-2.49245146	-2.01452465	-0.51903223
O	1.14826125	-0.38361088	1.11019173
C	1.38620209	4.52960421	-0.00428321
O	-1.13400818	1.82364016	0.33855978
C	-2.23206109	1.42071539	1.17775684
C	-3.55778666	1.93833602	0.62534184
C	-4.74456615	1.48484179	1.46707112
O	-5.93366896	1.95139411	0.85616411
C	-7.09060926	1.61244326	1.58699969
H	0.76662666	2.65400304	-1.02748232
H	2.95657717	2.40255139	0.71721484
H	1.67558435	2.07961965	2.00909157
H	2.22585013	-1.535458	-1.70903039
H	3.20119878	-1.84899142	-0.24511939
H	1.10650987	-2.40817334	0.78856402
H	-0.37462366	-0.47490272	-1.05132983
H	-0.09143989	-2.19064741	-1.46528546
H	-3.25347127	-2.32859526	0.19927407
H	-2.80536881	-1.06828447	-0.98525976
H	-2.40388203	-2.78031378	-1.30626949
H	2.41507182	4.49040489	-0.38052187
H	0.84903518	5.27771006	-0.60683714
H	1.39628605	4.8971092	1.02622774
H	-2.19078028	0.32833689	1.18795902
H	-2.06141134	1.79832722	2.18934987
H	-3.53442384	3.03295529	0.59772462
H	-3.69518078	1.58608784	-0.40442824
H	-4.76669826	0.3822574	1.54789189
H	-4.66148903	1.87978159	2.49473502
H	-7.94821359	2.0149424	1.04105408
H	-7.21010576	0.52022754	1.6833078
H	-7.077016	2.04638286	2.60026668

reaction (ix)

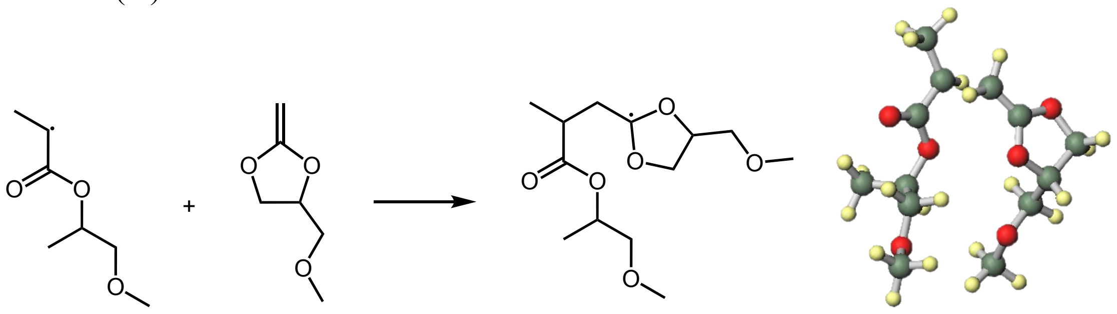

	x	y	z
O	0.98427194	3.33247118	1.83533488
C	1.08870581	2.67786805	0.80321237
C	2.32750214	2.10092492	0.30573073
C	2.33576768	0.17913366	1.39854045
C	1.50711009	-0.76858378	0.86201785
O	1.84205667	-1.5255703	-0.21910118
C	0.79818144	-2.48594435	-0.42304895
C	-0.41083543	-1.86126434	0.29259674
C	-1.3564327	-1.11454729	-0.63809797
O	-2.45063637	-0.65087726	0.11560931
C	-3.48258677	-0.10220932	-0.68502666
O	0.21778924	-0.97191296	1.23894398
C	3.61779812	2.71156769	0.77421653
O	0.02337148	2.35149516	0.00592074
C	-1.29125359	2.86495323	0.33910747
C	-1.37790313	4.38213987	0.19969641
C	-1.79113247	2.33518428	1.68725467
O	-3.21091577	2.45670661	1.66739594
C	-3.7954324	2.09968086	2.90131461
H	2.29409612	1.70286865	-0.70417452
H	3.39588463	0.05041918	1.21853767
H	2.04078569	0.62588204	2.34014471
H	0.65843711	-2.61414658	-1.49912642
H	1.09004844	-3.44415246	0.02264555
H	-0.98306034	-2.59017926	0.87226715
H	-0.8255573	-0.28269308	-1.12914816
H	-1.69017505	-1.81885992	-1.42252032
H	-4.20660968	0.34670655	-0.00411556
H	-3.10244878	0.68174299	-1.35634504
H	-3.96968409	-0.87939444	-1.29567382
H	4.47068269	2.04974385	0.58491369
H	3.82217596	3.65909047	0.25307537
H	3.57227952	2.93800919	1.84356917
H	-1.92209207	2.40456529	-0.42675716
H	-2.41518692	4.69209435	0.36329531
H	-0.73874953	4.8794074	0.93218001
H	-1.07593529	4.69321805	-0.80628511
H	-1.49855069	1.28145666	1.79464203
H	-1.36481312	2.9107813	2.51787331
H	-4.876705	2.23052704	2.79855372
H	-3.58435825	1.05081558	3.16387442
H	-3.43513862	2.73988499	3.72243362

reaction (x)

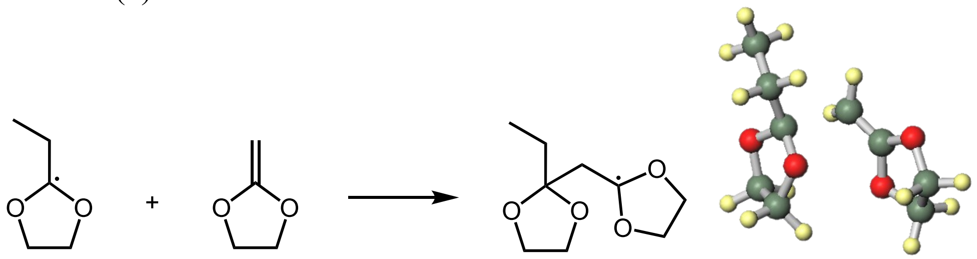

	x	y	z
C	-0.59734556	2.60835441	0.66825988
C	0.3483341	1.49913412	1.14070689
C	1.10080593	0.82938915	0.02206031
C	2.49224848	2.32120956	-0.97631314
C	3.76524417	1.83498662	-0.94085179
O	4.22901137	0.84281746	-1.77925307
C	5.58407376	0.57164506	-1.40288897
C	5.60364016	1.00325931	0.06296909
O	4.66419056	2.08367657	0.0651782
O	1.95595965	-0.19127083	0.45577693
C	1.84833282	-1.2903887	-0.45322963
C	1.031529	-0.71246212	-1.61484888
O	0.28064295	0.32202259	-0.98103978
H	-1.13225828	3.04461872	1.51881405
H	-0.05317212	3.4141098	0.16427658
H	-1.33825404	2.21516409	-0.03477643
H	1.08773724	1.89563834	1.84660098
H	-0.22575388	0.73129687	1.68716718
H	1.95056252	2.27072453	-1.91361267
H	2.27183423	3.15886244	-0.32544342
H	5.78449833	-0.49031182	-1.56205573
H	6.26986802	1.1724563	-2.01523697
H	5.25117463	0.20372374	0.72742422
H	6.5685233	1.37961793	0.40884767
H	2.84828715	-1.61368999	-0.7574175
H	1.33266492	-2.1232165	0.04305305
H	1.68642421	-0.2918246	-2.38855399
H	0.33106517	-1.41908773	-2.06617744

reaction (xi)

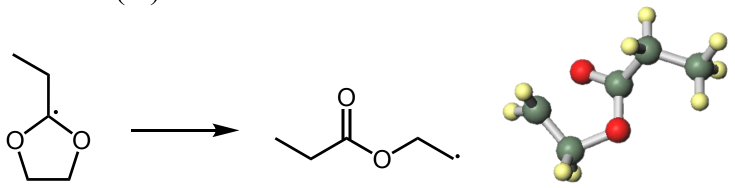

	x	y	z
C	3.60540349	-0.92338675	-0.59613373
C	2.35585677	-0.15912987	-0.14192363
C	1.06620044	-0.79018548	-0.60328943
O	0.90478385	-2.1039004	-0.29337575
C	-0.51011175	-2.34800476	0.0367323
C	-1.14922266	-1.03172477	0.33640853
O	-0.02751645	-0.13887523	-0.76339714
H	4.51213625	-0.43836746	-0.21948593
H	3.58769872	-1.95265099	-0.22518494
H	3.66702235	-0.96074937	-1.68922687
H	2.34324616	-0.09966874	0.9614472
H	2.36923064	0.86990096	-0.51452575
H	-0.97567141	-2.84095967	-0.82245165
H	-0.48713687	-3.03532764	0.88569125
H	-2.11314369	-0.80782049	-0.10516053
H	-0.98989069	-0.60690604	1.3245427

reaction (xii)

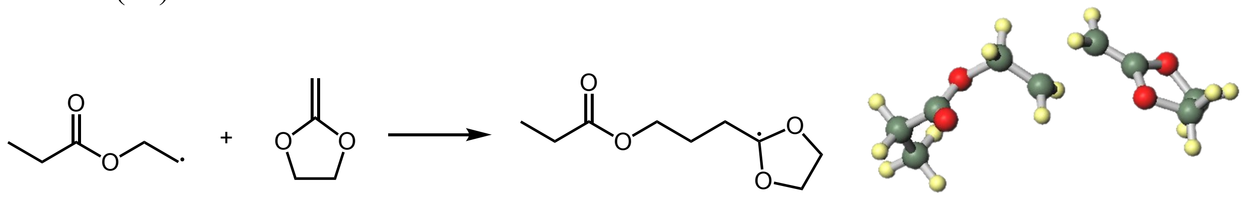

	x	y	z
C	7.02988051	5.26806013	-0.04987228
C	5.92433728	6.20764782	-0.55767943
C	4.56559374	5.53116506	-0.62592994
O	4.15658421	5.14693536	0.60127756
C	2.86092327	4.43851913	0.66695294
C	2.98244026	2.98721352	0.37322179
C	0.81350593	2.18089943	0.64945195
C	0.78298203	0.85492601	0.35925545
O	0.99376109	-0.13906734	1.27285175
C	1.27285683	-1.34377746	0.54442605
C	0.59772607	-1.0738219	-0.80021663
O	0.69087055	0.35301363	-0.90738868
O	3.93034741	5.35417892	-1.6454416
H	7.99335538	5.78826496	-0.02753239
H	6.80533555	4.9130787	0.95973798
H	7.13406535	4.39461054	-0.70316125
H	5.84505255	7.07417261	0.11061627
H	6.15090935	6.57351131	-1.56254809
H	2.53641533	4.6258757	1.69381085
H	2.1841935	4.93805384	-0.03039681
H	3.1329554	2.7043655	-0.66428299
H	3.49511094	2.36910961	1.10630178
H	0.78420253	2.47172192	1.69116847
H	0.42455403	2.8710882	-0.08726329
H	2.35825789	-1.47004561	0.44341441
H	0.85318001	-2.18724359	1.0960471
H	1.10856061	-1.51881394	-1.65620115
H	-0.45728928	-1.37623411	-0.80011627

reaction (xiii)

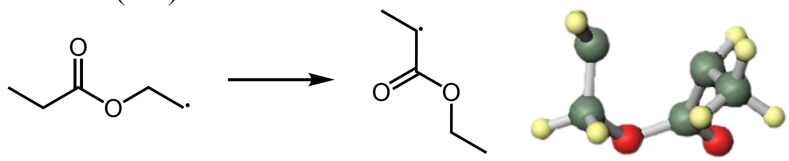

	x	y	z
C	0.97249719	0.0336054	-1.31302382
C	0.81714358	0.96686861	-0.12238794
C	-0.33353993	1.93809514	-0.19565836
O	-1.5452606	1.51341246	0.26538094
C	-1.7075459	0.18551084	0.81791461
C	-0.56821258	-0.20386918	1.72732473
O	-0.22944409	3.06484834	-0.62256102
H	1.8089542	-0.65590342	-1.15946339
H	0.06981927	-0.56121342	-1.49332656
H	1.17339011	0.60892163	-2.22649796
H	0.45131312	0.23008442	0.92667897
H	1.73597404	1.49269245	0.14341412
H	-2.66864089	0.23233128	1.34100004
H	-1.81194393	-0.53125893	-0.0063385
H	-0.44061853	-1.27393443	1.89097213
H	-0.4739496	0.39186513	2.63656618

reaction (xiv)

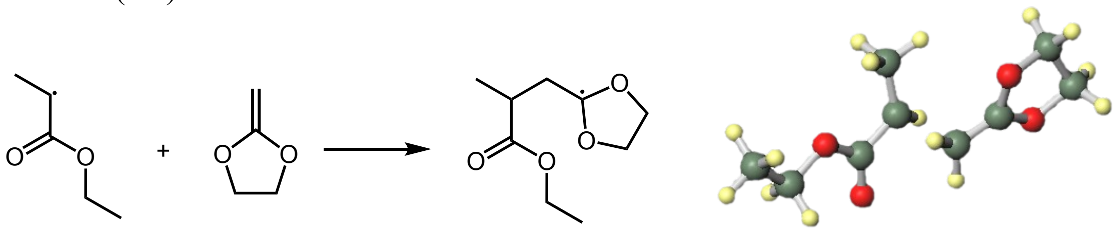

	x	y	z
C	3.5343622	-6.85836164	0.55643665
C	2.97621271	-6.17169312	-0.67736724
O	1.7504296	-5.52373993	-0.29621122
C	1.11553291	-4.83015907	-1.29348416
C	-0.08114034	-4.14602947	-0.82674562
C	0.82279381	-2.15186648	-0.48095124
C	-0.14050793	-1.23063606	-0.17949216
O	-0.55614241	-0.9371891	1.08670115
C	-1.80956361	-0.24280241	0.98355964
C	-1.74709261	0.33528588	-0.43050832
O	-0.9206931	-0.61813743	-1.11474641
C	-0.75574814	-4.50664698	0.46594075
O	1.57128799	-4.75379564	-2.42833537
H	4.47246719	-7.36840458	0.3105056
H	3.73546901	-6.13205233	1.35096058
H	2.82900333	-7.60146455	0.9428245
H	3.6703756	-5.42469531	-1.07792571
H	2.7711529	-6.88575464	-1.48256771
H	-0.71338793	-3.81384409	-1.64466371
H	1.4601733	-2.48220649	0.33025275
H	1.24831663	-2.12893899	-1.47651402
H	-2.63370342	-0.95705627	1.10328674
H	-1.85460185	0.50852994	1.77402816
H	-2.71179696	0.38344918	-0.93889425
H	-1.26568048	1.32091445	-0.45378233
H	-1.46775922	-3.72952441	0.76683398
H	-1.3194363	-5.44822071	0.37745652
H	-0.03039942	-4.64010793	1.27360986
